# Boron Complexes with Propiolamidinato Ligands: Synthesis, Structure, and Photophysical Properties

**DOI:** 10.1021/acs.inorgchem.4c01241

**Published:** 2024-06-14

**Authors:** Blanca Parra-Cadenas, Iván Bravo, M. Consuelo Ripoll Lorente, Carlos Ginés, David Elorriaga, Fernando Carrillo-Hermosilla

**Affiliations:** † Departamento de Química Inorgánica, Orgánica y Bioquímica-Centro de Innovación en Química Avanzada (ORFEO-CINQA), Facultad de Ciencias y Tecnologías Químicas, Universidad de Castilla-La Mancha, 13071 Ciudad Real, Spain; ‡ Grupo FOTOAIR, Unidad nanoDrug, Departamento de Química-Física, Facultad de Farmacia de Albacete, Universidad de Castilla-La Mancha, 02008 Albacete, Spain; § Departamento de Química Orgánica e Inorgánica, Universidad de Oviedo, Julián Clavería 8, 33006 Oviedo, Spain

## Abstract

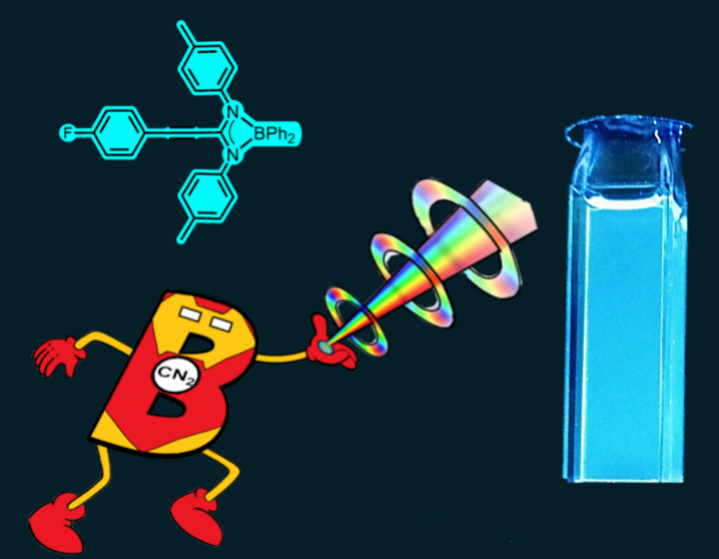

Two series of boron derivatives with propiolamidinato ligands, [BPh_2_{C(C≡CAr)(NR)_2_}] (Ar = Ph, *p*-MeOPh, *p*-FPh, *p*-Me_2_NPh, or phen; R = iPr or *p*-tolyl), were synthesized and structurally characterized. The corresponding propiolamidine (or propargylamidine) proligands have been obtained through sustainable methods. One is the catalytic hydroalkynylation of diisopropylcarbodiimide with different terminal alkynes, using simple ZnEt_2_ as a precatalyst. Alternatively, to obtain propiolamidines with aromatic groups on the nitrogen atoms, the formation of lithiated derivatives of terminal alkynes by reaction with *n*-BuLi in air and at room temperature, and subsequent addition to the di-*p*-tolylcarbodiimide, under the same conditions and using 2-MeTHF as a sustainable solvent, has been used for the first time. After reaction with BPh_3_, the corresponding boron amidinates were obtained, which are emissive in the solution state. The influence of the different substituents introduced into the ligands on the photophysical properties of the boron compounds has been studied. One of the obtained compounds can be used as a ratiometric fluorescent pH sensor in the acidic range.

## Abstract 2

Two series of boron derivatives with propiolamidinato ligands have been synthesized and structurally characterized. A distinct influence of the substituents of the ligands on specific photophysical properties of the compounds was observed. One of the obtained boron amidinates exhibits potential for application as a ratiometric fluorescent pH sensor in the acidic range.

## Introduction

**Scheme 1:**
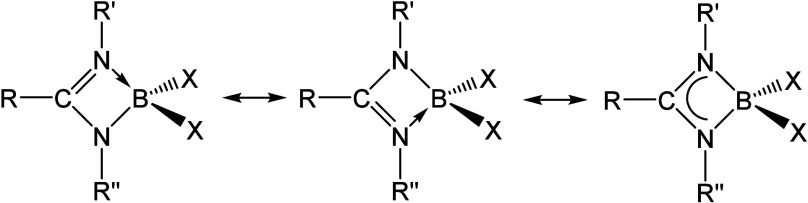
Resonant Forms of Boron Amidinato Derivatives

The continuing demand for new ligand systems that stabilize multiple types of metal or main group elements is a significant challenge in inorganic chemistry. The focus of this search is on chemistries that can induce electronic or steric properties in the resulting metal complexes, and thus, it results in a continued pressure in developing increasingly sophisticated compounds that can fulfill this mission. However, with the principle of “simpler is better” in mind, several simple organic molecules are gaining prominence in this field. Amidine derivatives, compounds with the general formula [RC(NR′R″)(NR′″)], are entities with the potential to overcome these requirements as they have demonstrated to be easily tunable to afford electronic and steric features as demanded by controlled variation of the substituents of the “CN_2_” core.^1^ Such is the effectiveness of these amidine compounds that they have been extensively studied acting as ligands with transition metals as they present great performance in order of coordination and stabilization of either low or high oxidation states.^2^ It is worth noting that aluminum amidinato complexes have been of great interest when it comes to main group elements, due to the discovery of useful applications in key technological areas such as catalysis or the development of new materials.^3^ On the contrary, boron derivatives did not have the same luck, and although there are some earlier precedents, described by the Dorokhov and Dehnicke groups,^4^ there was no equivalent development of boron amidinato chemistry and remains comparatively unexplored. Evidence of this disparity is that until the first decade of the 2000s the only boron compounds synthesized, in addition to those mentioned above, were limited to those obtained by the Cowly^5^ and Chivers groups.^6^ Structural studies have shown that the chelate-type coordination of the amidinato ligand forms a four-membered heterocycle, which can be described as an equal contribution from two diaza-allyl resonance forms, leading to the delocalization in the “CN_2_” core of the amidine (Scheme 1), something characteristic of this type of compound when coordinated to other elements of the periodic table, from metals to non-metals.^1^

More recently, Stephan’s group has also described the creation of boron amidinates by employing HB(C_6_F_5_)_2_ as the starting material and carrying out the insertion reactions of carbodiimides, showing fascinating frustrated Lewis pairs (FLPs).^7^ Additionally to this finding, Rojas et al. described the synthesis of a monodentate boron amidinato compound using HB(C_6_F_5_)_2_ and the corresponding amidine, which evolves to a bidentate coordination mode by dehydrogenation.^8^

In this vein and during the past several years, our laboratory has been actively engaged in developing the catalytic addition of amines^9^ and terminal alkynes^10^ to carbodiimides, employing the commercially available ZnEt_2_ as a catalyst, which offers a straightforward, atom-economical route for obtaining N,N′,N″-trisubstituted guanidines and N,N′-disubstituted propiolamidines, respectively. Moreover, we succeed in the study of these guanidine compounds as ligands with early^11^ and late^12^ transition metals. Recently, we turned our attention to the relatively less explored chemistry of boron guanidinates and amidinates,^13^ reporting the formation of a new monodentate boron amidinato compound and its capability to reduce CO_2_ into methanol, through a catalytic reaction, taking advantage of its FLP behavior.

On the contrary, many tetracoordinated boron compounds reported in the literature exhibit luminescent features. Usually, this type of compounds involves ligands containing a neutral, donor, nitrogen atom and a negatively charged nitrogen or oxygen atom,^14^ among which are the successful BODIPY derivatives. Several studies have revealed that the nature of ligands, π-electron-rich, and the substituent groups, both in the ligands and in the boron center, which possesses a vacant p orbital, have a strong influence on the photophysical properties of these compounds. These properties can be attributed to the electronic π → π* transitions within the chelates themselves or on the charge transfer transitions from the substituent groups to the chelate during the excitation process. Moreover, two key factors can enhance the fluorescence of these compounds: (i) the structural rigidity in the chelated compounds, which can restrict the molecular rotations, avoiding the quenching of the fluorescence, and (ii) the flat π-conjugated skeletons that can facilitate the charge transport due to the intermolecular delocalization of the π-electrons.^15^

On the contrary, the photophysical properties of boron amidinato compounds have been scarcely studied. Würthwein et al. described the use of tridentate ligands, which can be considered as amidinates with an additional donor group, for obtaining boron derivatives characterized by high thermal and chemical stability. Some of these compounds were used as dyes to obtain cell images.^16^

In the very recent past and during the course of this work, Chandrasekhar et al. have described a series of new luminescent boron compounds with amidinato ligands, of the type [Ar-C(tBuN)_2_BF_2_], in which the effect of increasing the level of π-conjugation for different polycyclic aromatic substituents has been studied. In all cases, the compounds were photoemissive only in the blue region.^17^

Given the novelty and potential interest of these types of compounds, our intention was to develop procedures that allow for greater modification of the amidinato ligands and to study the effect on their luminescent properties. Here we report the use of either catalytically or stoichiometrically synthesized propiolamidines, as sustainable methods, to prepare and structurally characterize a certain number of new boron amidinato complexes, paying special attention to how amidinato ligands coordinate to the boron center depending on their nature, and finally, we explore their photophysical properties.

## Discussion

### Synthesis and Characterization

**Scheme 2:**
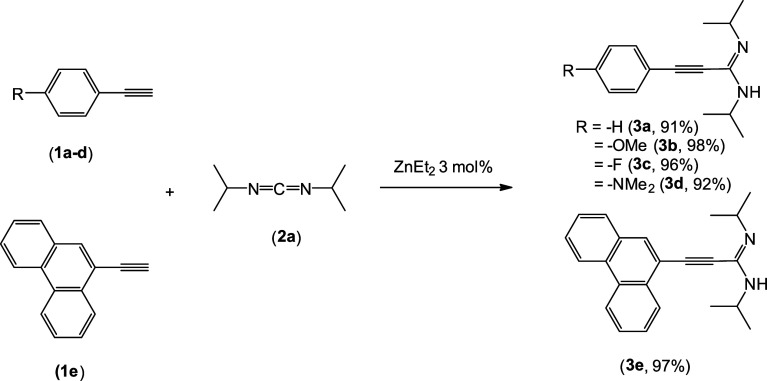
Catalytic Synthesis of Propiolamidines

Since the first amidine was reported in 1858,^18^ many different routes have been described for their synthesis. The most widely used strategy is Pinner’s reaction,^19^ although it presents several limitations like nitriles bearing electron-withdrawing groups at the α-position or the synthesis of sterically hindered and aromatic amidines. More recently, with the aim of overcoming those limitations, powerful catalytic methods were developed, where many compounds of transition metals, lanthanoids, and main group elements have been described as catalysts.^20^ As previously mentioned, we have made several contributions to this area. One such contribution was the selective and efficient synthesis of amidines. This was achieved by reacting a lithium amide formed in situ with an aromatic nitrile in sustainable solvents, under bench-type conditions, at room temperature.^21^ Furthermore, as previously described, we also reported a very successful catalytic addition of terminal alkynes to *N*,*N*′-diisopropylcarbodiimide employing commercially available ZnEt_2_ as a catalyst.^10^ Via the slightly modification of this catalytic procedure, specifically with an increase in the reaction temperature, propiolamidine compounds **3a**–**e** have been prepared in nearly quantitative yields (Scheme 2). Several commercially available phenylacetylenes substituted at the *para* position (**1a**–**d**), along with a more extended π-conjugated system, phenantrylacetylene (**1e**), were chosen as precursors for their synthesis to investigate the potential influence of electron-withdrawing and -donating groups, as well as a condensed aromatic system, on the properties of the final boron compound.

The reaction of **2a** with the different alkynes **1a**–**e** was carried out in toluene at 120 °C for 4 h (6 h for **3e** due to its lack of solubility), giving rise to the different proligands in the form of crystalline solids and excellent yields (91–98%). As we indicated in our previous report,^10^ there is not an explicit influence of the substituents in this catalytic reaction. It is worth noting at this point that some of the amidine compounds prepared in this study have been previously reported,^22,10^ although their reactivity was almost unexplored. Specifically, compounds **3d** and **3e** have been synthesized for the first time, and all amidines have been characterized using nuclear magnetic resonance (NMR) (see the Supporting Information). The ^1^H NMR spectra of these ligands show very similar patterns. In the downfield region, there are signals of the aromatic rings of the different alkynes. Moreover, the most representative set of signals of these compounds corresponds to the isopropyl groups being identified as two doublets, or broad multiplets (1.5–0.8 ppm) and a septuplet (∼4.3 ppm). With regard to their ^13^C NMR spectra, the most characteristic features are two signals of the triple-bond carbon atoms that can be found in the range of 80–90 ppm.

**Scheme 3:**
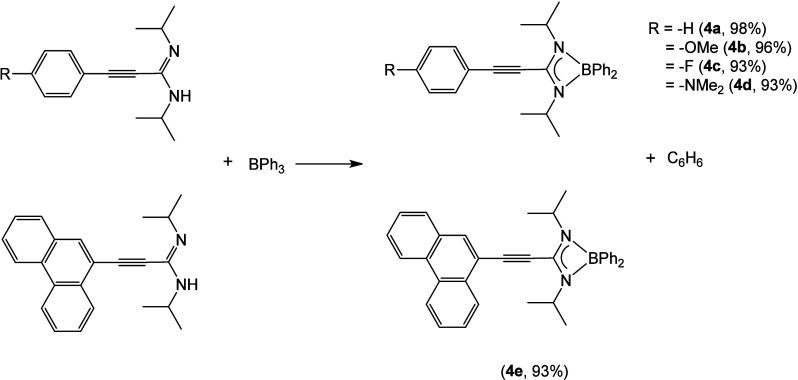
Synthesis of Boron Amidinates **4a**–**e**

After synthesizing the amidines, we proceeded to prepare subsequent boron complexes. Among the established methods for creating new amidinato compounds, the direct reaction between the ligand and a metal (or nonmetal) precursor, which contains ligands susceptible to cleavage by protonolysis, has been extensively utilized.^23^ Hence, to obtain the boron derivatives described in this work, we decided to follow a procedure similar to the one proposed in the literature for formazanate or iminopyrrolyl ligands,^24^ which is based in these protonolysis reactions. To achieve that aim, BPh_3_ was chosen as the boron precursor, because the reaction with the amidine ligands would give rise to the formation of the boron amidinato complex, with the concomitant elimination of benzene (Scheme 3). In fact, the reactions of BPh_3_ with 1 equiv of amidine proceeded smoothly at 120 °C in toluene for 4 h to provide nearly quantitative yields of compounds **4a**–**e** (93–98%), obtained as crystalline solids.

The excellent selectivity of the process toward the monosubstituted compounds should be noted. These compounds remain stable in solution for days under an inert atmosphere, for several hours when exposed to air in a solid state, and even for several minutes in an aqueous solution.

**Figure 1:**
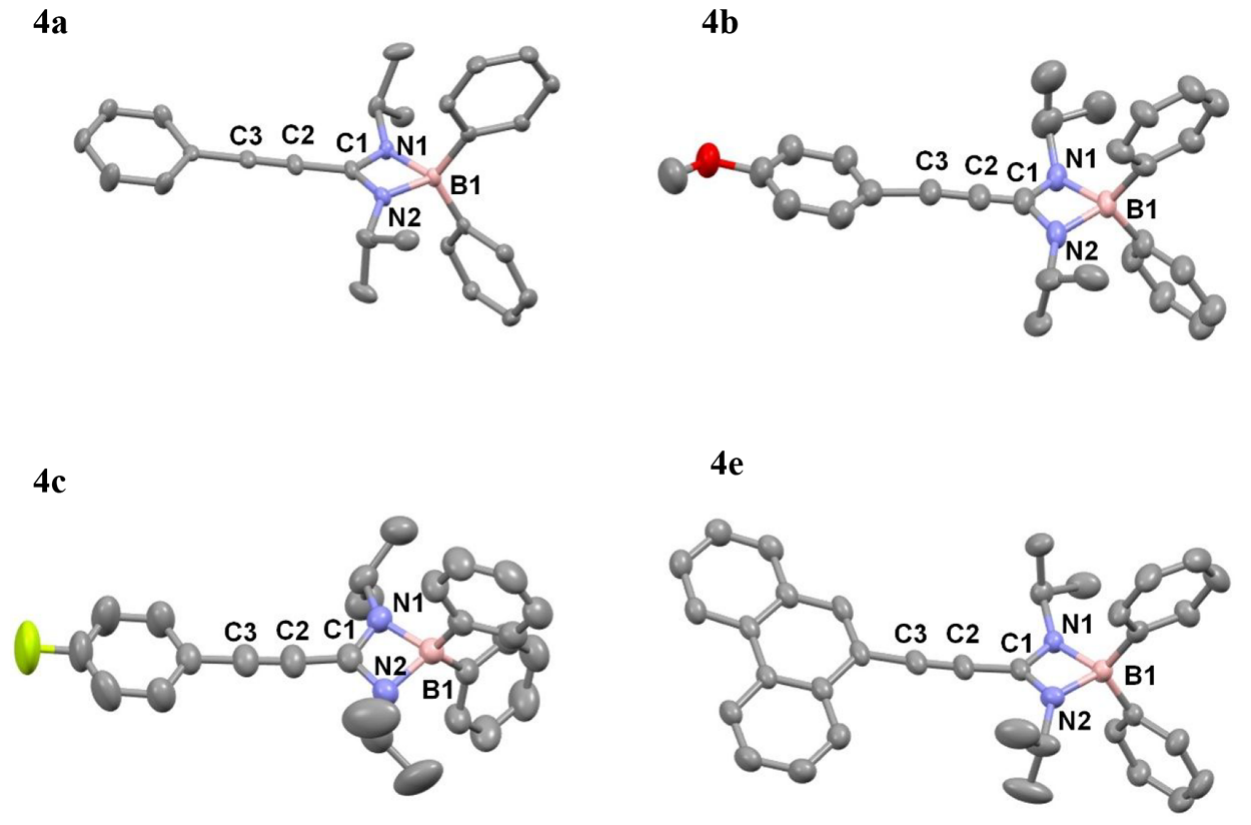
Molecular structures of **4a**–**c** and **4e**. H atoms and a pentane molecule in the **4e** structure have been omitted for the sake of clarity.

The new amidinato complexes (**4a**–**e**) were characterized by multinuclear NMR spectroscopy in solution and X-ray diffraction studies (details in the Supporting Information). The ^1^H NMR spectra of these compounds are quite similar. In the region of the aromatic signals, it can be found that it corresponds to the phenyl rings of the amidinato ligands and BPh_2_ fragments. Additionally, the two isopropyl groups of the amidinato ligand seem to be equivalent. The methyl and methine protons of the isopropyl groups appear as a doublet and a septuplet near δ 1.1 and 3.9 ppm, respectively. In the ^13^C NMR spectra, the same pattern as in the ^1^H NMR can be observed with only one set of signals for both isopropyl groups. This observation suggests that the molecules were highly symmetric. Furthermore, two signals, corresponding to the C≡C bond, can be found in the range of approximately 80–90 ppm. Additionally, a single ^11^B NMR resonance was observed at ∼10 ppm for **4a**–**e**. These data agree with a four-coordinate boron center with a *C*_2*v*_-symmetric coordination that consists of a pseudotetrahedral geometry. Fortunately, suitable X-ray-quality crystals of **4a–c** and **4e** were obtained from a saturated solution in pentane. The molecular structures and atomic numbering schemes are shown in Figure 1. Diffraction studies revealed mononuclear compounds in all cases and confirmed the coordination of the amidinato ligands in a chelate κ^2^ fashion, which produces distorted pseudotetrahedral coordination around the boron atoms (as predicted by NMR studies). The bite angles of the chelate ligands oscillate between 102° and 103°, and the N1–B1–N2 angle oscillates in the range of 78–80°, agreeing with those of the analogous reported compounds.^5,6,17^ In complexes **4a**–**c**, the methyl groups of the isopropyl point toward the boron sphere, whereas in complex **4e**, one of the isopropyl groups points to the boron atom and the other is focused backward. The C–N bond distances are intermediate between those anticipated for single and double bonds, which is indicative of delocalization at the amidinate core (C–N, ∼1.3 Å; the sum of the bond angles around C1 is ∼360°). The B–N bonds (∼1.60 Å) are similar in length and again fall in the range observed for B–N bonds in four-coordinate boron centers.^5,6^ The chelate ring and the aromatic ring of the amidinato ligand are almost coplanar, with dihedral angles for **4a**–**c** around 11.69–12.97°. The angle is slightly larger for **4e** (18.87°) due to the greater bulk demand by the condensed system. However, in all cases, the measurements fall within a range that suggests a certain degree of electronic delocalization throughout the entire system via the triple bond. The length of this bond barely fluctuates at ∼1.20 Å.

**Figure 2:**
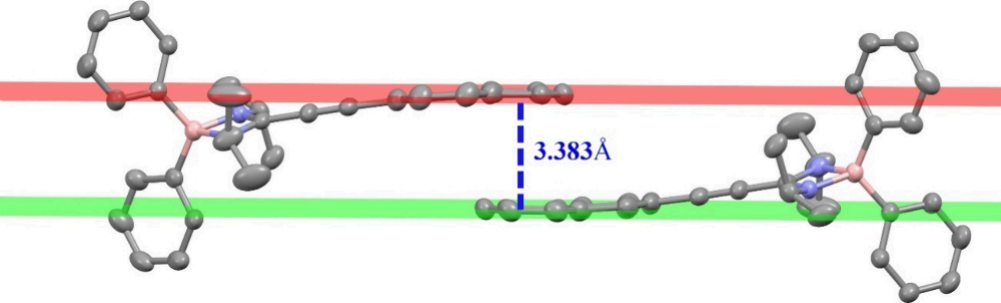
π-Stacking interaction scheme for complex **4e** in the solid state. Hydrogen atoms have been omitted for the sake of clarity.

In terms of crystal packing, compound **4e** positions one of the aromatic rings of the phenanthroline moiety almost overlapping with the same segment of a neighboring molecule. This arrangement is known as a parallel displacement or parallel offset geometry. The distance between the planes formed by the rings of the phenanthroline groups is 3.383 Å, which falls within the range described in the literature for π-stacking interactions.^25^ This contact leads to the formation of “dimeric” aggregates in the solid state (see Figure 2).

With all of these results in mind, it can be asserted that the nature of the substituents of the aromatic ring of the arylacetylene fragments does not have a clear influence on the coordination of the amidines to the boron center and their structural behavior. Therefore, we decided to change the substituents of the carbodiimide with the aim of examining the influence of those fragments on the formation and structural disposition of the boron amidinato complexes. As such, we chose the more rigid and bulky carbodiimide, *N,N*′-di-*p*-tolylcarbodiimide.

Once again, we began with the synthesis of the new amidines. However, all of our attempts to prepare them using our catalytic procedure were unsuccessful. As a result, we decided to introduce an innovative perspective by expanding our previous sustainable one-pot, two-step methodology, which operates with polar organometallics under air and uses sustainable solvents,^21^ for the preparation of the ligands. First, we optimized the reaction as it was never applied to the synthesis of propiolamidines (Table 1). To start this study, we took as a model the direct reaction of phenylacetylene **1a** with 1 equiv of *n*-BuLi in 2-MeTHF. The solvent was used as received from commercially available sources, with air and moisture. The addition of 1 equiv of *n*-BuLi for 5 s to a solution of **1a** in 2-MeTHF (under air and at room temperature) and then addition of 1 equiv of *N,N*′-di-*p*-tolylcarbodiimide **2b**, leaving stirring for 30 s before quenching with a saturated solution of NH_4_Cl, gave amidine **5a** in a good yield of 78% (entry 1, Table 1). This result demonstrates, contrary to the established idea, the possibility of obtaining this interesting type of ligands by means of a simple procedure that does not require special inert atmosphere conditions. When the time of the addition of *n*-BuLi was increased to 10 and 30 s (entries 2 and 3, Table 1) the yield decreased from 70% to 66%. On the contrary, keeping 5 s for the addition of the *n*-BuLi and increasing the reaction time before quenching to 60 s increase the yield to an excellent 98% (entry 4, Table 1). Moreover, under those conditions, switching the solvent to another sustainable and commercial one, CPME (cyclopentyl methyl ether), resulted in a decrease in yield (92%), as shown in entry 5 of Table 1. The use of 2-MeTHF, to which water was intentionally added, to obtain 1 wt %, results in a decrease in yield to 55% (entry 6, Table 1).

**Table 1:**
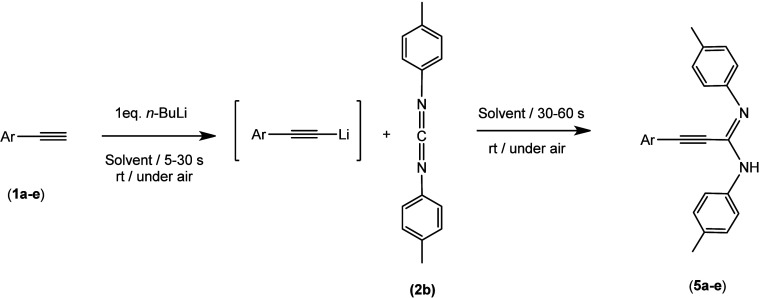
One-Pot, Two-Step Protocol for the Optimization of the Synthesis of Amidines **5a**–**e** at Room Temperature in the Presence of Air and Sustainable Solvents

entry	Ar–C≡CH	solvent	addition time (s)	reaction time (s)	yield (%)
1	Ph- (**1a**)	2-MeTHF	5	30	78 (**5a**)
2	Ph- (**1a**)	2-MeTHF	10	30	70 (**5a**)
3	Ph- (**1a**)	2-MeTHF	30	30	66 (**5a**)
4	Ph- (**1a**)	2-MeTHF	5	60	98 (**5a**)
5	Ph- (**1a**)	CPME	5	60	92 (**5a**)
6	Ph- (**1a**)	2-MeTHF (1 wt % water)	5	30	55 (**5a**)
7	*p*-MeO-(C_6_H_4_)- (**1b**)	2-MeTHF	5	60	84 (**5b**)
8	*p*-F-(C_6_H_4_)- (**1c**)	2-MeTHF	5	60	90 (**5c**)
9	*p*-NMe_2_-(C_6_H_4_)- (**1d**)	2-MeTHF	5	60	86 (**5d**)
10	phenanthryl (**1e**)	2-MeTHF	5	60	99 (**5e**)

In summary, the best reaction condition for the synthesis of these amidines can be set up as (i) addition of *n*-BuLi to phenylacetylene **1a** for 5 s in commercial 2-MeTHF, at room temperature under air, and (ii) subsequent addition of 1 equiv of carbodiimide **2b** and stirring for 60 s before quenching the reaction with a saturated solution of NH_4_Cl. With the reaction conditions optimized, we expanded the study to include the formation of analogous amidines **5a**–**e**, as shown in Table 1. With regard to the effect of the substituents, an electron-withdrawing group (**5c**) does not afford an improvement in the performance of the reaction. However, electron-donating groups (**5b** and **5d**) weakly influence the decrease of the yields, probably due to the less nucleophilic character of the intermediate lithium arylacetylide.

At this point, it is worth noting that only amidine **5a** has been previously described,^26^ with compounds **5b**–**e** being synthesized exclusively for this work (details of their characterization are given in the Supporting Information). The NMR spectra of these compounds are very simple and identical. In the ^1^H NMR spectra, in addition to the aromatic signals from the carbodiimide and the arylacetylene and its substituents, a singlet at ∼2.1 ppm can be identified, which corresponds to the methyl group of the *p*-tolyl moieties. With regard to the ^13^C NMR spectrum, as determined for the analogous amidines **3a**–**e**, the most representative signals correspond to the triple bond, and two peaks can be distinguished in the range of 80–95 ppm.

**Scheme 4:**
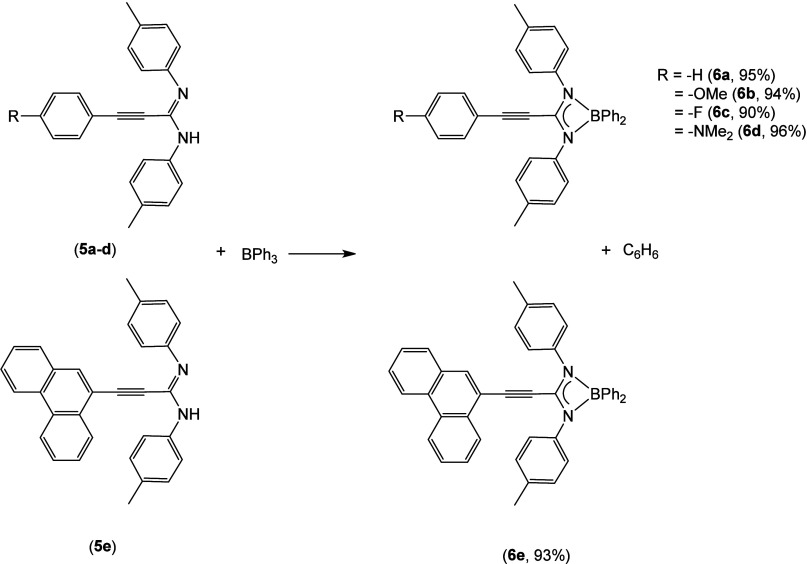
Synthesis of Boron Amidinates **6a**–**e**

As we did earlier in this study, once the propiolamidines were synthesized, we decided to investigate their coordination to a boron center. Thereby, the same protonolysis methodology for compounds **4a**–**e** was followed, using BPh_3_ as the boron source (Scheme 4).

**Figure 3:**
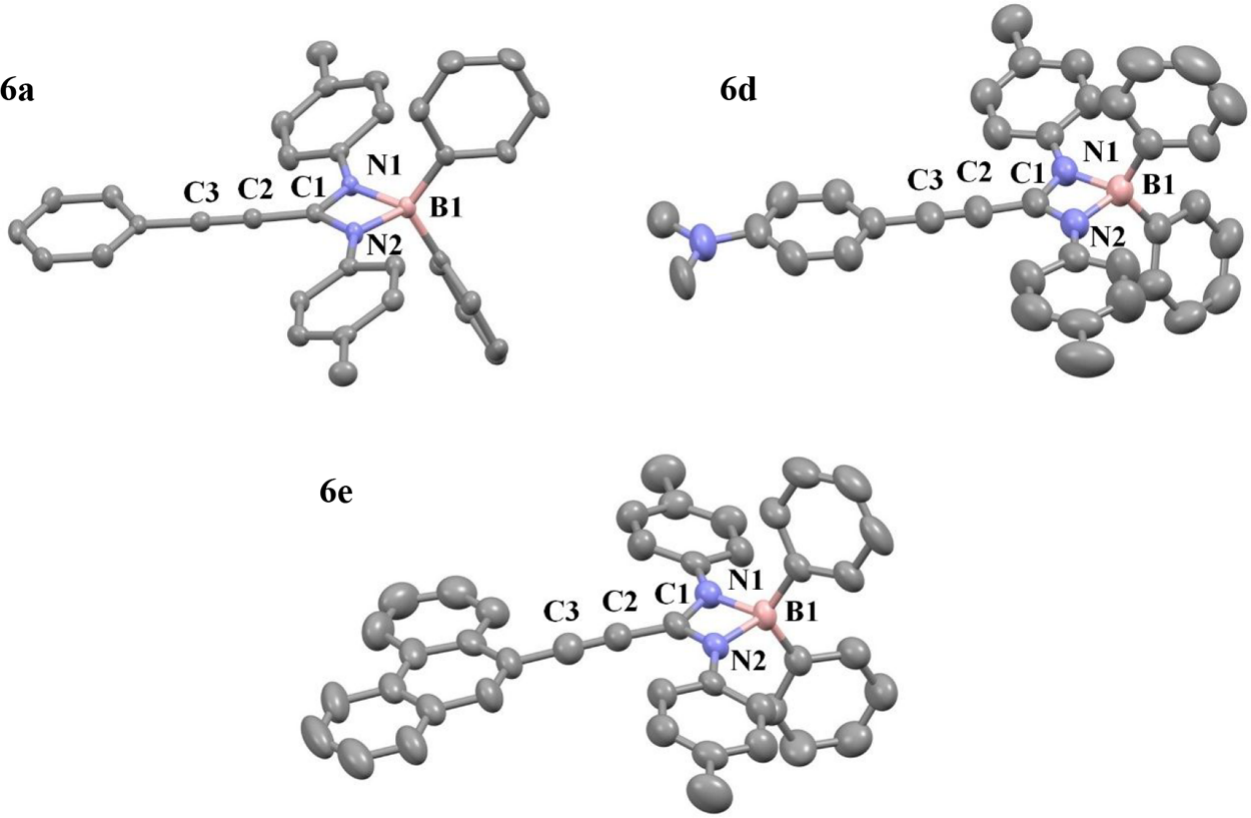
Molecular structures of **6a**, **6d**, and **6e**. Hydrogen atoms and a DCM molecule in the structure of **6e** have been omitted for the sake of clarity.

In this manner, the reaction of BPh_3_ with 1 equiv of the corresponding amidine in toluene at 120 °C for 5 h afforded the new boron complexes, along with benzene, in almost quantitative yields (90–96%) as crystalline solids. Again, the reaction demonstrates excellent selectivity toward the monosubstituted compounds. However, in contrast with analogous compounds **4a**–**e**, these new boron complexes are more sensitive to air, needing to be treated and stored under an inert atmosphere, even in the solid state. Hydrolysis results in the formation of insoluble solids and free amidine. These amidinato complexes **6a**–**e** were fully characterized by multinuclear NMR and X-ray diffraction. In the ^1^H NMR spectra, in addition to the signals of the aromatic rings and the substituents of the arylacetylene fragments, the most notable feature is a singlet signal, which falls around 2 ppm. This is slightly more shielded than in the free ligand and corresponds to the methyl group of the tolyl moieties, indicating a highly symmetric coordination of the amidinato ligands. Furthermore, the same conclusion can be drawn from the ^13^C NMR spectra, where again only one signal for the same group is observed at ∼20 ppm. Additionally, the resonances of the triple bonds are found within the range of 80–100 ppm, which is slightly broader than that observed in the free amidines. Additionally, ^11^B NMR spectroscopy shows a broad single resonance at ∼10 ppm and reveals a four-coordinate boron center. All of these results, as in compounds **3a**–**e**, confirm a *C*_2*v*_-symmetric coordination in a pseudotetrahedral geometry around the boron atom. Opportunely, single crystals of **6a**, **6d**, and **6e** suitable for X-ray diffraction were obtained from saturated solutions of a DCM/pentane/benzene mixture. The molecular structures and the atomic numbering schemes are shown in Figure 3.

**Figure 4:**
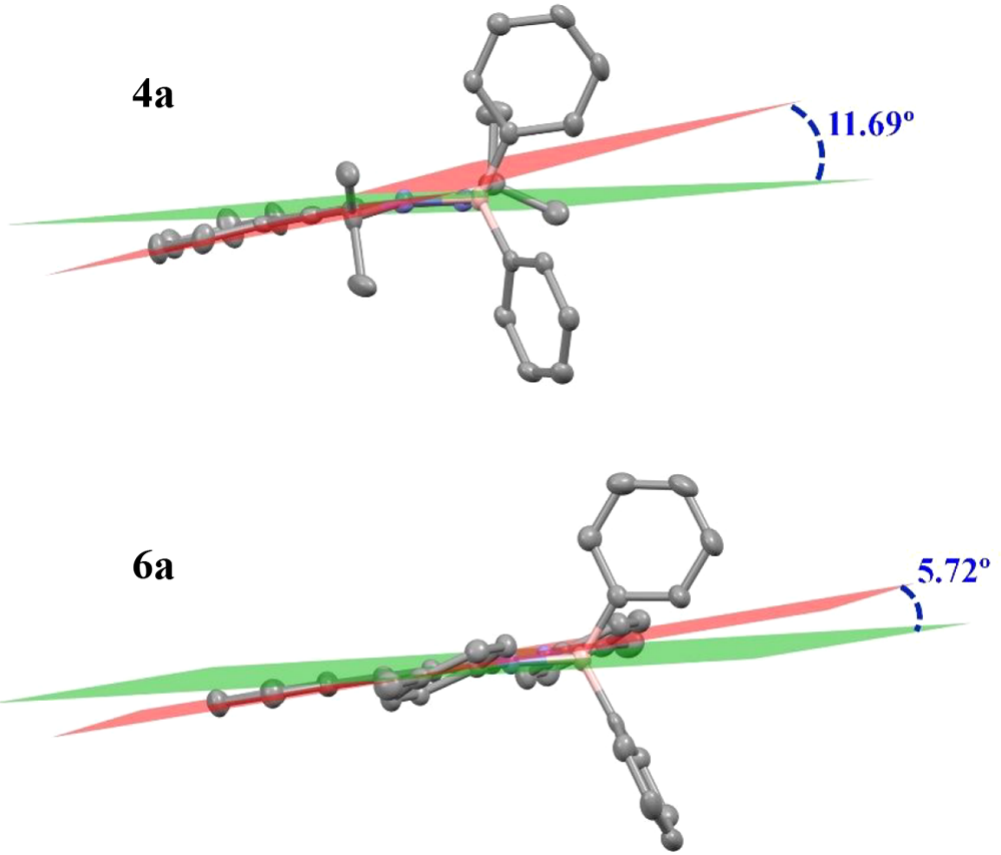
Dihedral angles between planes (red for the phenyl ring plane and green for the chelate ring plane) in compounds **4a** and **6a**.

Structural XRD studies showed the amidinato ligands in a κ^2^-coordination mode, giving rise to a pseudotetrahedral geometry around the boron atom, which had been predicted by NMR studies. The N1–B1–N2 angles are in the same range as those of complexes **4a**–**c** and **4e**. Moreover, the bite angles for these complexes are ∼101°, being slightly smaller than those of the analogues with isopropyl groups on the nitrogen atoms. On the contrary, the C–N distances fall within the range of 1.33 Å, which is between a single and double bond. Additionally, the sum of the angles around C1 is nearly close to 360°. This suggests electronic delocalization in the CN_2_ core. The B–N distances maintain a length of ∼1.6 Å. In contrast to the compounds with the isopropyl groups in the nitrogen atoms, the dihedral angle between the planes formed by the chelate rings and the aromatic fragment from the arylacetylene is smaller (∼5°), which indicates a major electronic delocalization over the whole π-conjugated system through the C≡C bond (Figure 4 shows planes and dihedral angles in compounds **4a** and **6a** as an example). These bond lengths are very close to 1.19 Å, being slight shorter than those of the analogues with isopropyl groups. This observation aligns with the concept of a system with greater electronic delocalization.

**Figure 5:**
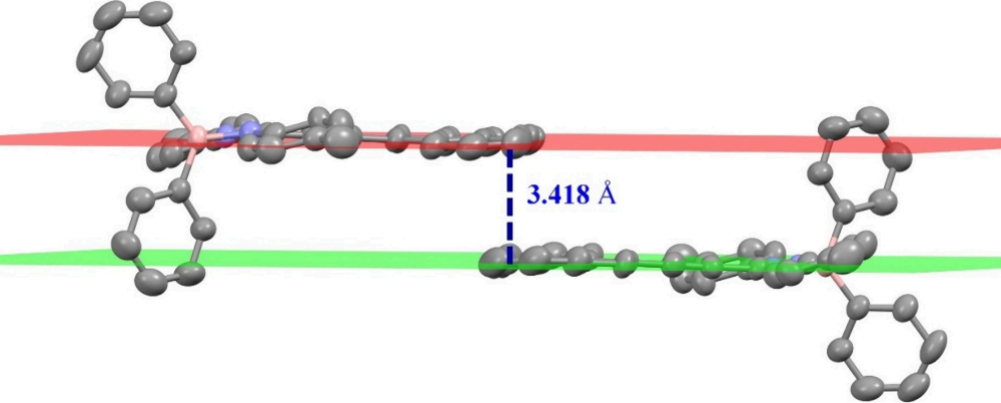
π-Stacking interaction scheme for complex **6e** in the solid state. Hydrogen atoms have been omitted for the sake of clarity.

With regard to the packing in the crystals, for compound **6e**, similar to **4e**, a π-stacking interaction can be observed between one aromatic ring of the phenanthrene group of one molecule and the same part of the neighboring molecule. The distance between planes is 3.418 Å (see Figure 5), forming “dimeric” aggregates in the solid state.

**Figure 6:**
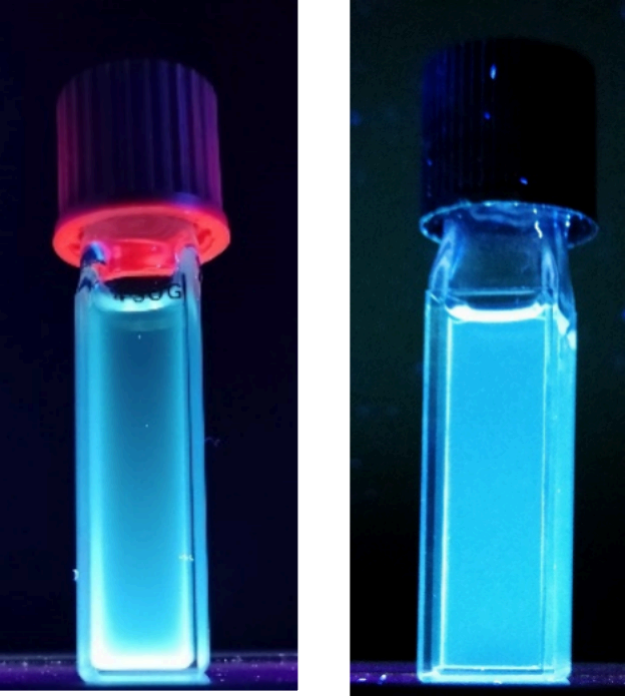
Samples of compounds **4b** and **6c** (10 μM in CH_2_Cl_2_) under UV light (hand-held UV lamp, 365 nm).

Surprisingly, when all boron compounds were exposed to ultraviolet (UV) light, a characteristic fluorescence was observed, showing emissions in the blue region (Figure 6). On the contrary, when the amidine compounds were exposed to the same UV light, no fluorescence or only very weak fluorescence was observed (Figure S2). Thus, the luminescence of the boron complexes is probably related to the electronic delocalization through the whole molecule, favoring charge transfer transitions from the ligand to the boron atom. Moreover, this transition is supported by the formation of the chelate with the boron atom and the subsequent increase in the rigidity of the complexes. To shed light on such a phenomenon, we explored the photophysical behavior of these boron amidinato complexes.

### Photophysical Properties of Boron Amidinato Complexes

**Figure 7:**
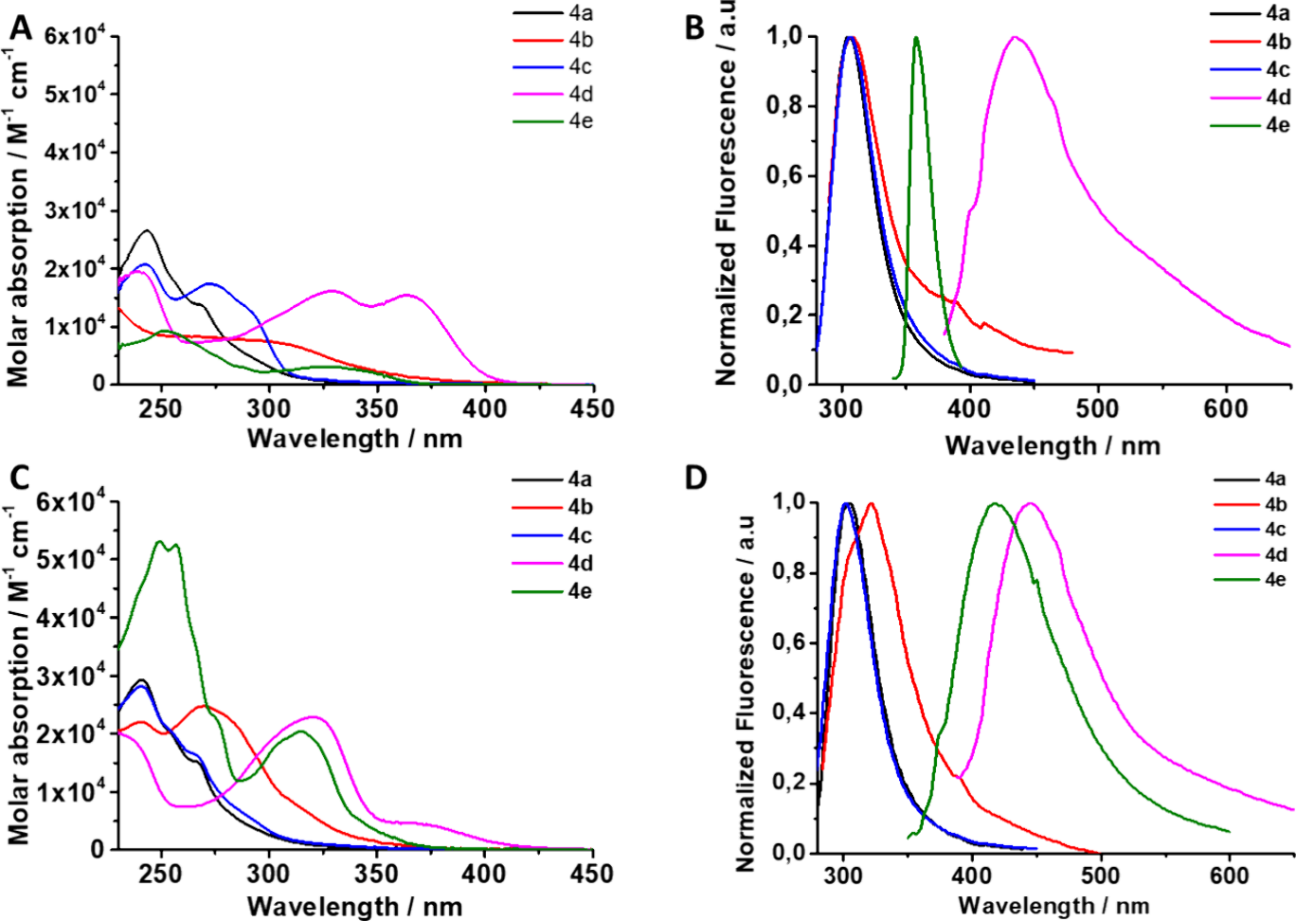
(A) Absorption and (B) emission spectra of compounds **4a**–**e** in CH_2_Cl_2_ (10 μM) and (C) absorption and (D) emission spectra in CH_3_CN of compounds **4a**–**e** (10 μM).

First, we decided to focus on the absorption and emission features of series **4** compounds. Thus, the absorption and emission spectra of compounds **4a**–**e** in acetonitrile and dichloromethane were recorded. As shown in Figure 7, with regard to absorption spectra, we can point out that all complexes reveal mains peaks within the ranges of 220–300 and 300–400 nm, which can be associated with electronic π → π* transitions in the chelates themselves and the charge transfer transitions from the substituent groups to the chelate during the excitation process, respectively. Interestingly, the substituents of the aromatic fragment of the amidinato ligands seem to show a clear influence on the absorption properties. Thereby, in compounds **4a** and **4c**, with a neutral (H) or an electron-withdrawing substituent (F), the absorption in the range of 300–400 nm was very low compared to that of compounds **4b** and **4d**, with electron-donating groups (OMe and NMe_2_, respectively). This finding can be explained by the fact that electron-donating groups might increase the extent of charge transfer and therefore the absorption in such a range. In addition, it appears that solvent polarity does not significantly affect absorption spectra, although for **4e** it is favored in acetonitrile. For **4d**, an additional absorption band appears at ∼360 nm in acetonitrile and dichloromethane. This is likely related to the protonated species, a consequence of the presence of small traces of water in the solvents.^27^ We will discuss such phenomena along with the pH behavior of compound **4d** in the following sections.

**Figure 8:**
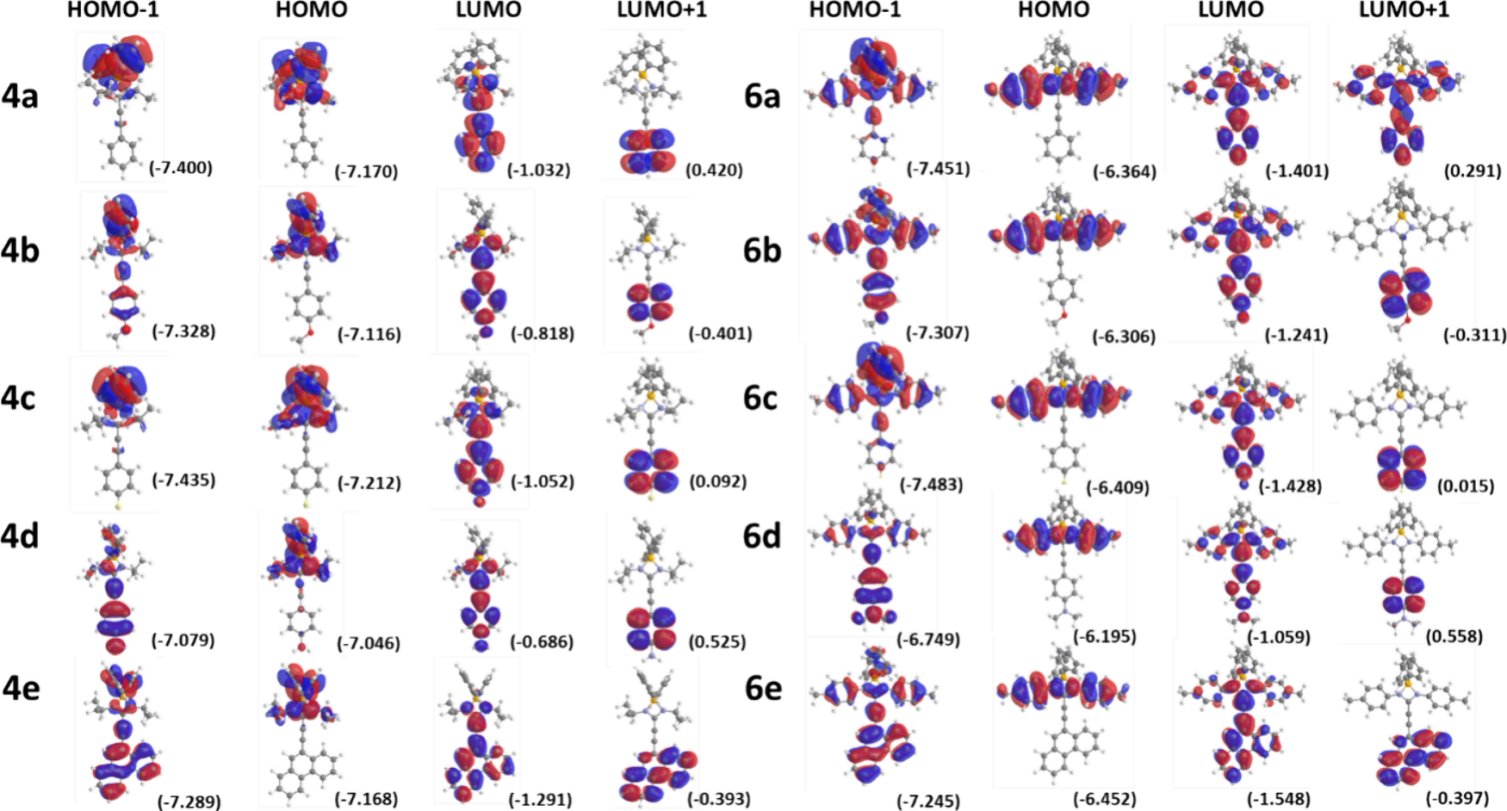
Molecular orbitals of compounds **4a**–**e** and **6a**–**e** calculated at the m062*x*/6-31g* level of theory in the gas phase (eigenvalues in parentheses).

**Figure 9:**
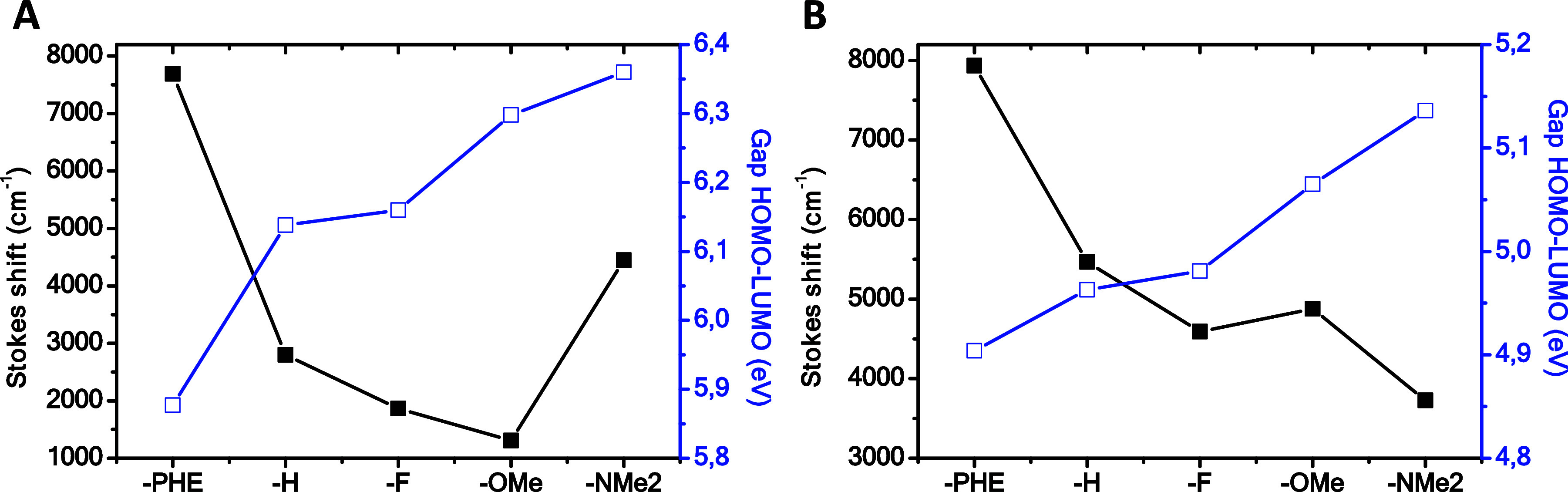
Relationship between the Stokes shift (acetonitrile) and HOMO–LUMO gap vs the substituent in the alkynyl group in (A) **4a**–**e** and (B) **6a**–**e**.

Aiming to shed light on the nature of such electronic transitions, we decided to explore them by means of density functional theory calculations. As shown in Table 2, the vertical transition data fit well with the absorption spectra with the transitions centered around 350 and 270 nm. In addition, these transitions are complex, involving several HOMOs and LUMOs (Figure 8), where the charge is transferred from the phenyl groups of the boron atom through the chelate to the alkynyl substituent. Consequently, substituents such as OMe, NMe_2_, and phenanthrene help to stabilize the charge, probably due to the most pronounced conjugative effect of these motifs, as represented by the charge location in the HOMO–1 orbitals for **4b**, **4d**, and **4e**. On the contrary, a substituent such as H or F in **4a** and **4c** destabilizes the excited state, a finding that is in agreement with experimental absorption and emission data. A more graphical view of this effect is shown in Figure 9, which represents the influence of the alkynyl substituent on the Stokes shift and the HOMO–LUMO gap. We observed that a decrease in the gap leads to an increase in the Stokes shift. This trend can be observed for F and OMe substituents in compounds **4b** and **4c** in comparison to **4a** with the H substituent, where the greater electronegativity for F and the inclusion of a donor group such as OMe considerably increase the HOMO–LUMO gap and, consequently, decrease the Stokes shift. An unusual behavior was observed for **4d**, where surprisingly an increase in the HOMO–LUMO gap was associated with a larger Stokes shift. This can be explained by the greater charge delocalization provided by the NMe_2_ group, which stabilizes the excited state. At this point, it is worth noting that this is related to the previously discussed HOMO–1 orbital behavior. Conversely, the presence of a polyaromatic system, a phenanthrene ring (**4e**), dramatically decreases the HOMO–LUMO gap and increases the level of charge transfer with the largest Stokes shift in the series of ∼100 nm (7690 cm^–1^). Therefore, in response to our original hypothesis, we found that the emission spectrum can be tuned by the electronic features of the alkynyl substituent in the amidinato ligand of the **4a**–**e** series. Related to the solvent effect, as polar solvents stabilize the excited state, larger Stoke shifts were observed for acetonitrile than for dichloromethane. The variety and complexity of electronic transitions in the **4a**–**e** series, coupled with ineffective stabilization of the excited state, imply that fluorescence deactivation is not the predominant process. This results in low quantum yields across the entire **4a**–**e** series, and only with the introduction of the phenanthrene group in **4e** is it possible to achieve a quantum yield of 3.6% in dichloromethane (Table 3).

**Table 2:** Vertical Transition Wavelengths (λ_vert_^calc^) Calculated for Compounds **4a**–**e** and **6a**–**e** at the m062x/6-31G* Level of Theory in the Gas Phase Along with the Oscillator strengths (*f*) and Main Components of the Transition

compound	λ_vert_^calc^ (nm)	*f*	main component of the transition (>15% contribution)
**4a**	356.60	0.000	H-5 → L (60%), H-4 → L (17%)
	293.57	0.079	H → L (58%), H-4 → L (20%)
	265.35	0.380	H → L+1 (41%), H-4 → L (30%), H-8 → L (20%)
**4b**	371.99	0.000	H-2 → L (53%), H-1 → L (27%)
	286.50	0.086	H → L (63%), H-5 → L (22%), H → L+3 (15%)
	273.66	1.038	H-2 → L (47%), H-1 → L (38%), H-3 → L (15%)
**4c**	359.19	0.000	H-5 → L (63%), H-4 → L (15%)
	292.80	0.081	H → L (59%), H-4 → L (20%)
	265.25	0.522	H → L+1 (41%), H-4 → L (30%), H-8 → L (20%)
**4d**	390.38	0.000	H-1 → L (74%), H-1 → L+4 (15%)
	283.79	0.109	H → L (65%), H-5 → L (21%), H → L+3 (15%)
	281.73	1.140	H-1 → L (100%)
**4e**	323.35	0.000	H-5 → L (46%), H-5 → L+1 (27%)
	301.00	0.718	H-1 → L (58%), H-2 → L (20%)
	297.01	0.065	H → L (58%), H → L+2 (23%), H-6 → L (19%)
**6a**	385.91	0.455	H → L (87%), H → L+1 (13%)
	292.69	0.464	H-1 → L (35%), H-2 → L (27%), H-4 → L (25%)
**6b**	377.27	0.486	H → L (100%)
	300.88	0.739	H-1 → L (69%), H-4 → L (16%), H-2 → L (15%)
**6c**	384.74	0.461	H → L (100%)
	293.31	0.491	H-1 → L (35%), H-2 → L (28%), H-3 → L (25%)
**6d**	370.59	0.510	H → L (100%)
	323.89	1.018	H-1 → L (100%)
**6e**	382.85	0.343	H → L (79%), H → L+1 (21%)
	321.86	0.728	H-1 → L (100%)

**Table 3:** Summary of Different Photophysical Parameters Such as Emission Peak Maximum Wavelengths, Stokes Shifts, HOMO–LUMO Energy Gaps, Fluorescence Lifetimes, and Quantum Yields for the **4a**–**e** and **6a**–**e** Series

	emission peak maximum wavelength (nm)		Stokes shift (nm) (cm^–1^)			average lifetime (ns)		quantum yield (%)	
	CH_3_CN	CH_2_Cl_2_	CH_3_CN	CH_2_Cl_2_	*E*_HOMO–LUMO_ (eV)	CH_3_CN	CH_2_Cl_2_	CH_3_CN	CH_2_Cl_2_
**4 a**	305	304	24 (2800.3)	23 (2692.5)	6.138	<1	<1	<1	<1
**4 b**	322	307	13 (1306.8)	15 (1673.3)	6.298	<1	<1	<1	<1
**4 c**	301	307	16 (1865.1)	19 (2148.9)	6.160	<1	<1	<1	<1
**4 d**	445	435	75 (4446.0)	71 (4446.3)	6.360	2.03	4.21	<1	<1
**4 e**	415	357	100 (7690.0)	35 (3044.7)	5.877	1.38	4.57	<1	3.59
**6 a**	382	382	66 (5467.6)	61 (4974.6)	4.963	24.2	26.9	<1	<1
**6 b**	382	376	60 (4877.9)	63 (5353.1)	5.065	17.3	22.4	<1	<1
**6 c**	382	382	57 (4591.2)	62 (5072.0)	4.981	4.15	11.8	<1	<1
**6 d**	375	376	46 (3728.5)	50 (4079.1)	5.136	8.72	17.6	<1	<1
**6 e**	375	376	86 (7935.4)	88 (8126.5)	4.904	15.63	19.3	<1	<1

**Figure 10:**
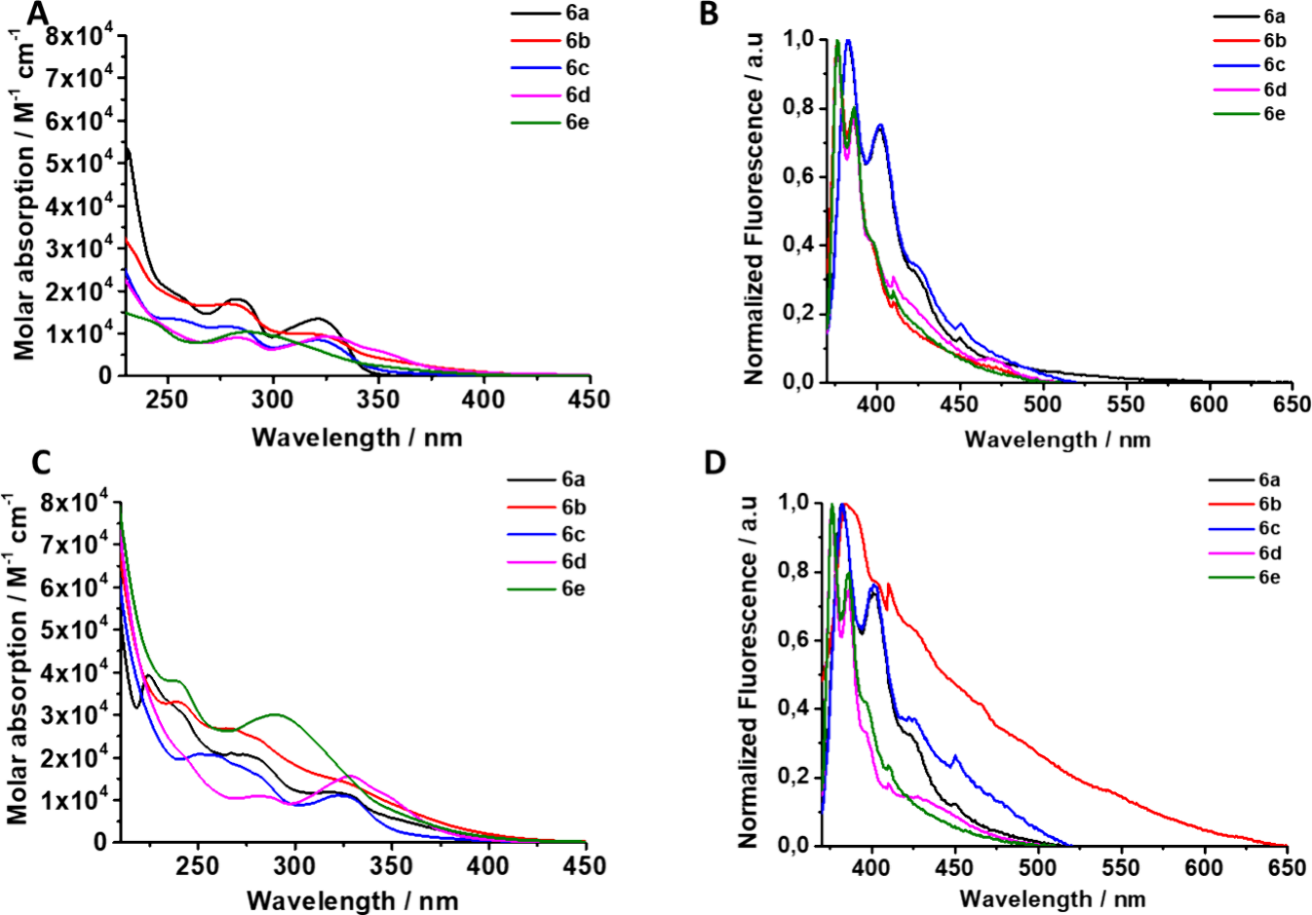
(A) Absorption and (B) emission spectra of compounds **6a**–**e** in CH_2_Cl_2_ (10 μM) and (C) absorption and (D) emission spectra in CH_3_CN of compounds **6a**–**e** (10 μM).

Keeping in mind the effect of the substituents in the aromatic ring of the alkynyl motifs on the photophysical properties of compounds **4a**–**e**, we find that the versatility of the proposed synthetic methods enables us to investigate the impact of swapping the isopropyl groups for the *p*-tolyl ones in compounds **6a**–**e**. This modification was expected to significantly modify the photophysical behavior of boron derivatives **6a**–**e** compared to that of **4a**–**e**, promoting charge transfer and stabilization in the excited state. Thus, the absorption and emission spectra of compounds **6a**–**e** in acetonitrile and dichloromethane were recorded as usual. Again, compounds **6a**–**e** exhibited primary peaks within the ranges of 220–300 and 300–400 nm. The substituent of the alkynyl fragment of the amidinato ligands appears to primarily influence the absorption properties within the range of 350–400 nm, in a manner consistent with the **4a**–**e** series. Focusing on series **6**, we can see that the absorptions of compounds **6a** and **6c** are significantly lower than those of compounds **6b**, **6d**, and **6e** (Figure 10A,C). Additionally, the vertical transition data fit well with the absorption spectra with the transitions centered around 380 and 290 nm (Table 2). It is worth noting that, contrary to those of the **4a**–**e** series, these transitions are well-defined, involving almost exclusively the HOMO–1–LUMO and HOMO–LUMO transitions. This aligns with the highest values of the oscillator strengths. The HOMO–1–LUMO transition involves charge transfer from the phenyl groups of the boron to the alkynyl substituent on the amidinato ligand. Unlike the previous series, in this case, the HOMO–LUMO transition implies charge transfer from the tolyl groups to the alkynyl substituent within the same chelate ligand. Therefore, the incorporation of the tolyl groups changes the photophysical features of the system, indicating that the nature of the substituent in the alkynyl group does not have as much influence on the stabilization of the excited state as in the **4a**–**e** series with isopropyl moieties. As a result, the emission spectra for all compounds in the **6a**–**e** series were very similar to those of a marked vibronic structure related to the well-defined electronic transitions (Figure 10B,D). The Stokes shifts are significantly larger for **6a**–**d** than for the **4** series (see Table 3 and Figure 9), within the range of 46–66 nm (3728–5468 cm^–1^). As in compound **4e**, the incorporation of a phenanthrene group in **6e** considerably increases the shift to 88 nm (8127 cm^–1^). On the contrary, for this series, the decrease in the HOMO–LUMO gap increases the Stokes shift for the entire series (Figure 9). With regard to the solvent effect, in this case, the influence of the polarity was less pronounced, and the Stokes shift values were comparable in acetonitrile and dichloromethane. Hence, changing the isopropyl substituents to tolyl substituents in the boron chelate compounds results in a transformation of the photophysical dynamics of the system by aiding in the stabilization of the excited state. Thus, the fluorescence lifetime of the **6a**–**e** series was dramatically increased with values between 4 and 27 ns (Table 3). In the case of compounds **4a**–**c**, measurement of the fluorescence lifetime was not feasible. However, when dissolved in dichloromethane, compounds **4d** and **4e** exhibited fluorescence lifetimes of 4.21 and 4.57 ns, respectively. A relevant increment in lifetime was noted for compounds **6a**–**e**, highlighting compound **6a**, which showed a fluorescence lifetime of 26.9 ns in dichloromethane. Unfortunately, the stability of the excited state, which results in an increase in the fluorescence lifetimes, does not correspond to a substantial enhancement in deactivation through the fluorescence mechanism, leading to quantum yields of ∼1% for the **6a**–**e** series. This observation implies the involvement of alternative deactivation mechanisms, such as internal conversion or phosphorescence.

**Figure 11:**
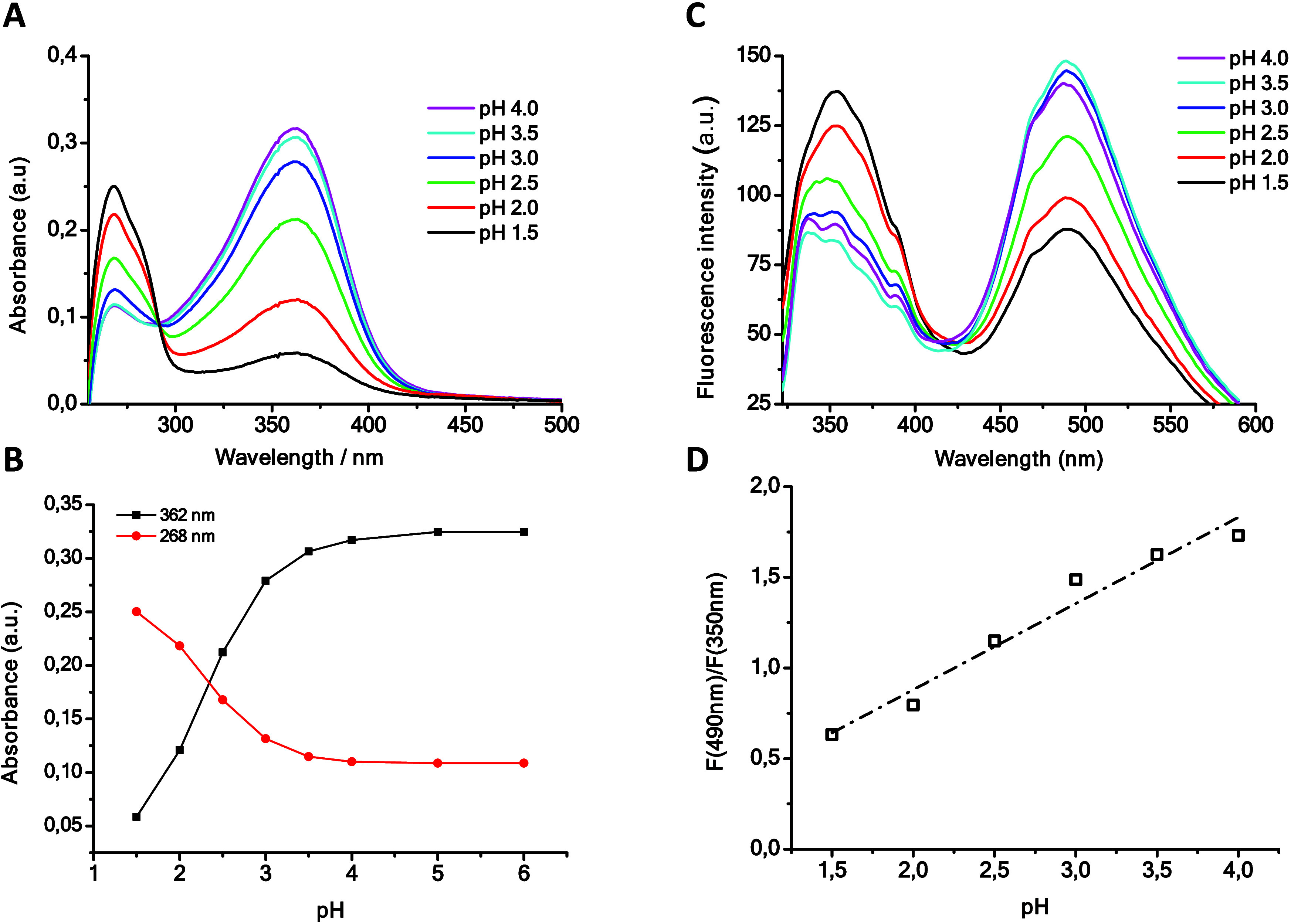
(A) Absorbance spectra of compound **4d** in water (10 μM) at different pH values. (B) Change in the intensity of the absorption maximum for both bands with pH. (C) Fluorescence emission intensity spectra of compound **4d** at different pH values. (D) Relationship of the ratio between the fluorescence intensity of its maxima and pH.

The measurement of the pH of a solution is a critical parameter in the field of chemistry. Traditional measurement methods, unfortunately, cannot be applied in certain contexts. Many of these challenges can be addressed by optically measuring the pH values. This approach has been utilized in the development of diagnostic tools, including luminescent molecular sensors, which have broad applications in various scientific and medical fields.^28^ In this sense, the NMe_2_ group and its potential for protonation could induce a pH effect on its photophysical behavior; i.e., the protonation of this group might influence its possible transfer of electron density toward the rest of the molecule. As previously discussed, the stability in water of both series is somewhat compromised. However, it was observed that **4d** exhibits relatively good stability and was therefore an excellent candidate for studying the possible effect of pH on its properties. Thus, when the pH effect was measured, it was observed that an increase in pH produced a decrease in the band at 265 nm and an increase in the band at 360 nm, with an isosbestic point close to 290 nm, in the absorption spectra of **4d** in water (Figure 11A). This was indicative of an equilibrium between the protonated and unprotonated forms of the molecule, depending on the applied pH. In a similar manner, their fluorescence spectra (Figure 11C) undergo significant changes with pH, and an increase in pH results in an enhanced signal at 490 nm and a diminished signal at 350 nm. These changes again can be readily associated with the deprotonated and protonated species, respectively. In fact, as a model, the reaction between 1 equiv of **4d** and 1 equiv of triflic acid in CD_3_CN results in a shift and a broadening of the NMe_2_ moiety signal, in the ^1^H NMR spectrum, compared to that of the original compound (see Figure S57). Moreover, Figure 11B shows the change in intensity of the absorption maximum for both bands with pH, allowing us to determine a p*K*_a_ of 2.34. On the contrary, when experiments were run at higher pH values, the compound decomposes, probably due to the attack of the OH^–^ anions of the basic medium on the boron center. Finally, we found an excellent linear relationship of the ratio between the fluorescence intensity of its maxima and pH value,  (*R* = 0.98), which suggests its possible use as a ratiometric fluorescent pH sensor in the acidic range (Figure 11D).

## Conclusions

In summary, a catalytic procedure for the hydroalkynylation of carbodiimides, using commercial ZnEt_2_ as the precatalyst, has been improved and extended to the synthesis of new propiolamidines. Alternatively, we have demonstrated that polar organolithium reagents, i.e., alkynyllithiums, generated in situ, can be used in a bench-type process, under air, in a sustainable and nondried solvent at room temperature, to obtain these amidines, which paves the way for a simpler and more sustainable synthesis of these kinds of compounds. Using these proligands and through a simple reaction with BPh_3_, with benzene as a byproduct that is easy to eliminate, access to a representative series of boron amidinato derivatives, which have been widely characterized, can be gained. Both the substituent on the carbon atom of the amidine core and that on the nitrogen atoms can be modified, allowing for a fine-tuning to study the effect on their properties. Specifically, the obtained compounds are luminescent after being excited with UV irradiation, emitting in the blue region, and their photophysical properties are dependent on the type of ligand studied. Although stability to air or water is somewhat limited, one of the synthesized compounds exhibits a change in its light absorption and emission behavior depending on the pH of the medium, which could allow its use as a ratiometric fluorescent pH sensor in the acidic range. Further studies of other boron derivatives that exhibit interesting photophysical properties by direct modification of substituents on amidines are underway in our laboratory.

## Supporting Information Available

Experimental procedure and characterization details (NMR spectra) for all compounds and X-ray crystallographic data for complexes for **4a–c**, **4e**, **6a**, **6d**, and **6e** (PDF)
