# Supplementary material for: Boron Complexes with Propiolamidinato Ligands: Synthesis, Structure, and Photophysical Properties
Source: Inorg Chem. 2024 Jun 14;63(26):12120–32. doi: 10.1021/acs.inorgchem.4c01241 (PMC11220760; doi:10.1021/acs.inorgchem.4c01241)
Supplement: Supplementary file 1 — ic4c01241_si_001.pdf [file ic4c01241_si_001.pdf]

Supporting Information

Boron complexes with propiolamidinato ligands: synthesis, structure and photophysical properties

Blanca Parra-Cadenas,[a] Iván Bravo,[b] M. Consuelo Ripoll Lorente,[b] Carlos Ginés,[a] David Elorriaga,[,c] Fernando Carrillo-Hermosilla.[*,a]*

[a] Departamento de Química Inorgánica, Orgánica y Bioquímica-Centro de Innovación en Química Avanzada (ORFEO−CINQA), Facultad de Ciencias y Tecnologías Químicas, Universidad de Castilla-La Mancha, 13071 Ciudad Real, Spain

[b] Grupo FOTOAIR, Unidad nanoDrug, Departamento de Química-Física, Facultad de Farmacia de Albacete, Universidad de Castilla-La Mancha, 02008 Albacete, Spain

[c] Departamento de Química Orgánica e Inorgánica, Universidad de Oviedo, Julián Clavería 8, 33006 Oviedo, Spain.

Corresponding author: Fernando.Carrillo@uclm.es

1.- General Methods and Materials

All manipulations were performed under a nitrogen atmosphere using standard Schlenk and glove-box techniques. The solvents used for the syntheses and for the NMR experiments were all anhydrous. All reagents were obtained from commercial suppliers (Sigma Aldrich) and used without further purification, with the exception of N,N′-di-*p*-tolylcarbodiimide, which was synthesized as described in the literature. [1]

NMR spectra were recorded on Bruker Avance Neo 400 and 500 spectrometers at 298 K, using standard TOPSPIN 4.0 software. Chemical shifts (δ) are given in ppm and coupling constants (*J*) in Hz. The chemical shifts were assigned based on homo- and heteronuclear 2D NMR experiments (COSY, HSQC, HMBC). Characterisation details, including ^{1}H, $^{13}C\{^{1}H\}$, $^{19}F\{^{1}H\}$ (when necessary) and $^{11}B\{^{1}H\}$ (when necessary) NMR spectra for compounds **3a-e, 5a-e, 4a-e** and **6a-e** are included in the following sections of this Supporting Information. Melting points were measured with a Büchi M-56 apparatus and are uncorrected. Elemental analysis data were recorded on a Foss-Heraeus CHNO-Rapid analyzer. Infrared spectra were recorded on a Bruker Tensor 27 spectrometer, using an ATR accessory. X-Ray diffraction studies were performanced on a Bruker APEX II CCD-based diffractometer equipped with a graphite monochromated MoKα radiation source (λ=0.71073 Å).

1.1.- Photophysical properties.

UV−visible absorption spectra were acquired on a Cary 100 (Varian) spectrophotometer at room temperature, using a slit width of 0.4 nm and a scan rate of 600 nm min−1. Steady state fluorescence (SSF) and time resolved fluorescence (TRF) spectra were recorded on an FLS920 spectrofluorometer (Edinburgh Instruments) equipped with a Microchannel plate-photomultiplier tube (MCP-PMT) detector (R3809) and a time correlated single photon counting (TCSPC) data acquisition card (TCC900model). Ten-millimetre quartz hermetic cuvettes (Hellma Analytics) were employed for all spectroscopic measurements. For SSF spectra, a 450 W Xe lamp was

used, and the light source and the excitation and emission slits were fixed at different size to 1-10 nm. For TRF experiments, an EPLED 291 and 368 sub-nanosecond pulsed light emitting diode (Edinburgh Photonics) was employed as light source.

The fluorescence intensity decays, I(t), were fitted by using an iterative least-squares fit method with the following multiexponential function (Eq. S1):

Equation S1 $$I(t) = \sum_{i=1}^{n} \alpha_i \exp\left(-{}^{t}/_{\tau_i}\right)$$

where α_i and τ_i are the amplitude and lifetime for each ith term. The average decay lifetime (τ_m) was then calculated using Equation S2:

Equation S2 $$\tau_m = \frac{\sum_{i=1}^{n} \alpha_i \tau_i^2}{\sum_{i=1}^{n} \alpha_i \tau_i}$$

A sample concentration of 10 μM was generally employed for UV−vis absorption and fluorescence spectroscopy experiments.

Quantum yields were measured in a FS5 spectrometer (Edinburgh Instruments) equipped with an integrating sphere, a 150W Xe lamp as the light source and a photomultiplier tube (PMT) detector (R928P model). Quantum calculations were carried out using the F980 Software of Edinburgh Instruments.

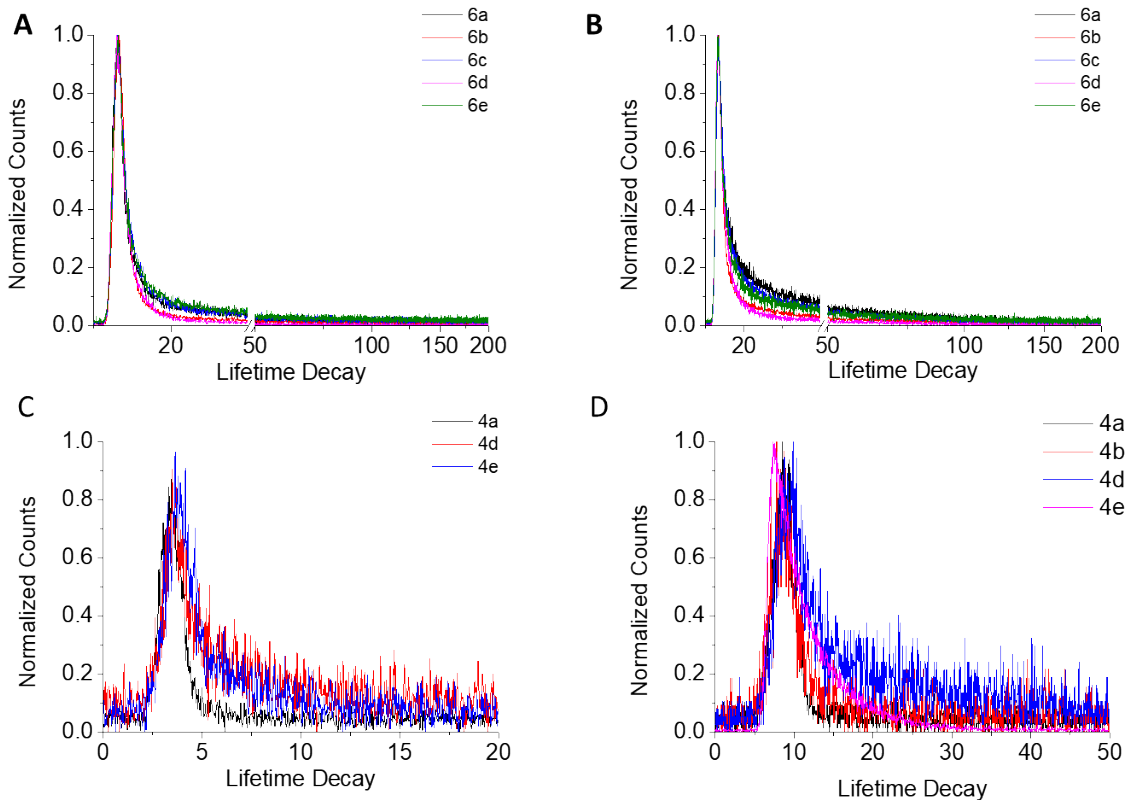

Figure S1. Lifetime decay profiles of compounds **6a-e** in acetonitrile (A) and Dichlorometane (B). Lifetime decay profiles of compounds **4a-d-e** in acetonitrile (C) and compounds **4a-b-d-e** in dichlorometane (D).

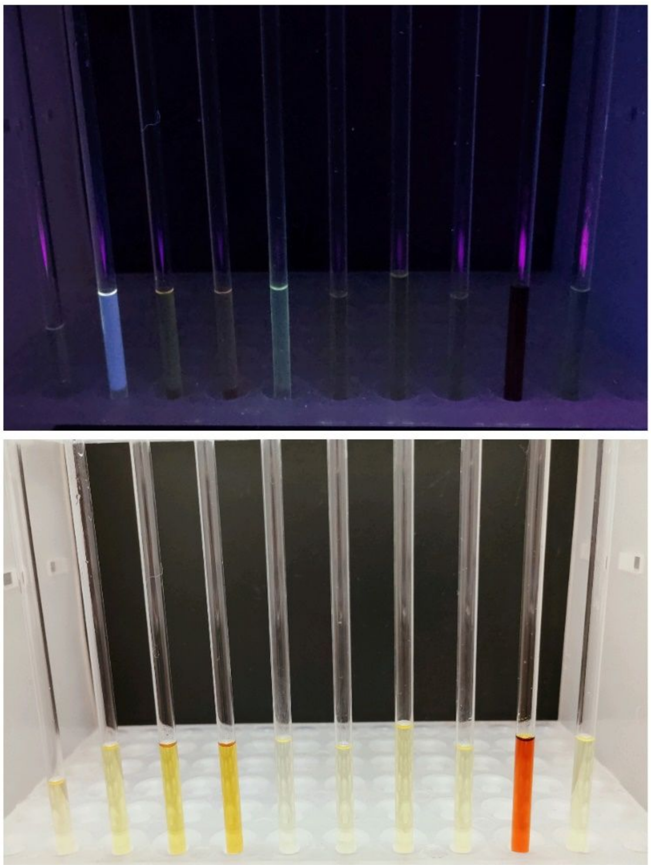

(A)

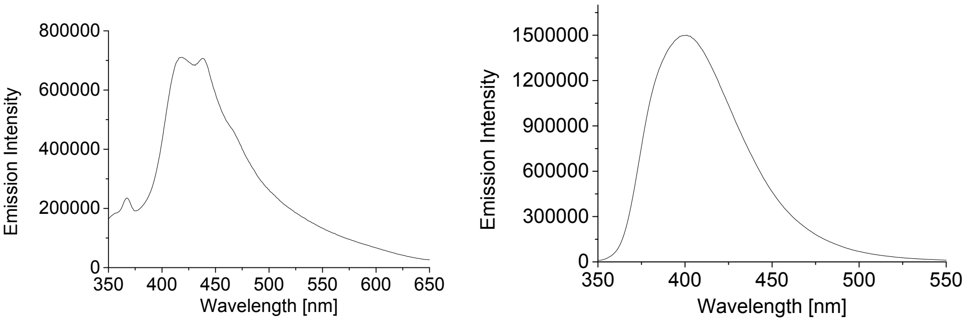

(B)

Figure S2. (A) Samples of the compounds **3a**, **3d**, **3b**, **3c**, **3e**, **5a**, **5d**, **5b**, **5c**, **5e** (from left to right) in CH_2Cl_2, under UV light (top, handheld UV lamp, 365 nm) and under natural light (bottom). (B) Emission spectra of compounds **3d** (left) and **3e** (right) in CH_2Cl_2 (10 μM).

1.2.- DFT calculations

Density functional theory (DFT) calculations were carried out to optimize the molecular structure of compounds. m062x as implemented in Gaussian16 (revision C.01), [2] along with the 6-31G* basis sets were used for the molecular structure optimization.

The electronic vertical transitions were calculated at the time-dependent (TD)-m062x/6-31g*.

2.- Experimental procedure and characterisation details

2.1.- General procedure for the obtaining of propiolamidines by catalytic methods, compounds 3a-e.

Syntheses of compounds **3a-e** were performed under N_2 atmosphere. In a schlenk, 1 equivalent of the appropriate alkyne (**1a-e**, $3\cdot10^{-3}$ mol) was dissolved in 5 mL of toluene, followed by the addition of 3mol% $ZnEt_2$ (1 M in hexanes) ($9\cdot10^{-5}$ mol). Subsequently, 1 equivalent of N,N'-diisopropylcarbodiimide (**2a**, $3\cdot10^{-3}$ mol) was added and the solution was stirred for 4h at 120ºC (6h in the case of **3e** due to its lack of solubility). The solvent was eliminated in vacuo. Then, 5 mL of pentane were added, and the mixture was placed in a refrigerator at −20 °C for 16 h, affording colorless powder which was filtrated and dried under reduced pressure. Compounds **3a-e** were obtained as yellowish microcrystalline solids with excellent yields (91–98%).

2.2.- General procedure for the obtaining of propiolamidines by stoichiometric methods, compounds 5a-e.

Syntheses of compounds **5a-e** were performed under air and at room temperature. In a Schlenk flask, 1 equivalent of the appropriate alkyne (**1a-e**, $3\cdot10^{-3}$ mol) was dissolved in 5 mL of 2-MeTHF, followed by the addition (under stirring) of 1 equivalent of nBuLi (1.6 M in hexanes) ($3\cdot10^{-3}$ mol) (addition time = 5 seconds) which gave rise to the lithium amidinate intermediate. Subsequently, 1 equivalent of N,N'-di-*p*-tolylcarbodiimide (**2b**, $3\cdot10^{-3}$ mol) was added and the solution was stirred for 60 seconds before quenching with 5 mL of a saturated solution of NH_4Cl. The mixture was extracted with 2-MeTHF (3 x 5 mL). The combined organic phases were dried over anhydrous $MgSO_4$ and the solvent was concentrated in vacuo. Compounds **5a-e** were obtained as yellow or orangish microcrystalline solids with good to excellent yields (84–99%).

2.3.- General procedure for the obtaining of boron complexes, compounds 4a-e and 6a-e.

Syntheses of compounds **4a-e** and **6a-e** were performed under N_2 atmosphere. In a Schlenk flask, 1 equivalent of the appropriate amidine (**3a-e** or **5a-e**, $1\cdot10^{-3}$ mol) was

dissolved in 5 mL of toluene, followed by the addition of 1 equivalent of BPh_3 ($1\cdot10^{-3}$ mol). The solution was stirred for 4h at 120ºC (series **4a-e**) and for 5h at 120ºC (series **6a-e**). The solvent was eliminated in vacuo. Then, 5 mL of pentane were added, and the mixture was placed in a refrigerator at −20 °C for 16 h, affording crystalline solid which was filtrated and dried under reduced pressure. Compounds **4a-e** were obtained as yellowish microcrystalline solids with fantastic yields (93–98%) while compounds **6a-e** were obtained as orange to reddish microcrystalline solids with excellent yields (90–96%).

2.4.- Characterisation details

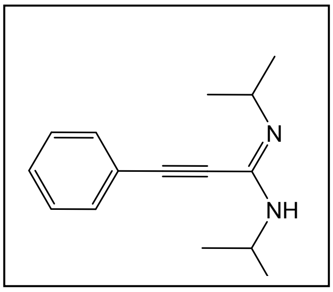

3a: FT-IR (cm^{-1}) = NH 3382, C≡C 2227, C=N 1568. ^{1}H NMR (C_6D_6) δ (ppm) = 0.99 (d, J = 6.3 Hz, 6H, CH_3), 1.39 (d, J = 5.3 Hz, 6H, CH_3), 3.81 (bs, 1H, NH), 4.21-4.27 (m, 2H, CH), 6.96-6.98 (m, 3Harom), 7.38-7.40 (m, 2Harom). $^{13}C\{^{1}H\}$ NMR (C_6D_6) δ (ppm) = 22.7 (2C), 25.5 (2C), 42.7, 53.2, 80.9, 90.4, 122.2, 128.8 (2C), 129.4, 132.2 (2C), 140.1. Elem. anal. calc for [$C_{15}H_{20}N_2$]: C, 78.90; H, 8.83; N, 12.27. Found: C, 78.83; H, 8.92; N, 12.35.

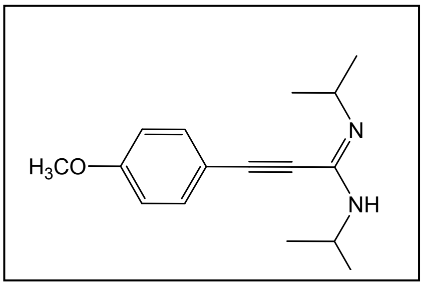

3b: FT-IR (cm^{-1}) = NH 3390, C≡C 2223, C=N 1565. ^{1}H NMR (C_6D_6) δ (ppm) = 1.04 (bs, 6H, CH_3), 1.41 (bs, 6H, CH_3), 3.17 (s, 3H, CH_3O), 3.81 (bs, 1H, NH), 4.26-4.34 (m, 2H, CH), 6.57-6.59 (m, 2Harom), 7.36-7.38 (m, 2Harom). $^{13}C\{^{1}H\}$ NMR (C_6D_6) δ (ppm) = 24.0 (2C), 25.3 (2C), 49.2, 53.1, 54.9, 79.9, 90.8, 114.1, 114.6 (2C), 133.8 (2C), 140.6, 160.8. Elem. anal. calc for [$C_{16}H_{22}N_2O$]: C, 74.38; H, 8.58; N, 10.84. Found: C, 74.44; H, 8.68; N, 10.92.

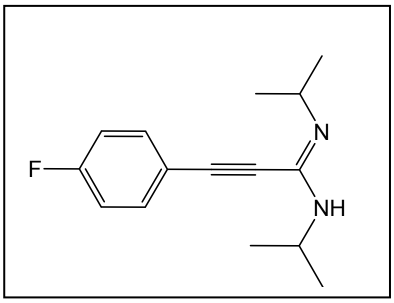

3c: FT-IR (cm^{-1}) = NH 3388, C≡C 2220, C=N 1562. ^{1}H NMR (C_6D_6) δ (ppm) = 0.99 (d, J = 6.4 Hz, 6H, CH_3), 1.39 (d, J = 6.2 Hz, 6H, CH_3), 3.77 (bs, 1H, NH), 4.17-4.26 (m, 2H, CH), 6.56-6.60 (m, 2Harom), 7.10-7.15 (m, 2Harom). $^{13}C\{^{1}H\}$ NMR (C_6D_6) δ (ppm) = 22.7 (2C), 25.5 (2C), 42.7, 53.2, 80.6, 89.2, 115.9, 116.2, 118.1,

134.2, 139.9, 162.1, 164.6. $^{19}F\{^{1}H\}$ NMR (C_6D_6) δ (ppm) = -109.14. Elem. anal. calc for [$C_{15}H_{19}FN_2$]: C, 73.14; H, 7.77; N, 11.37. Found: C, 73.24; H, 7.90; N, 11.42.

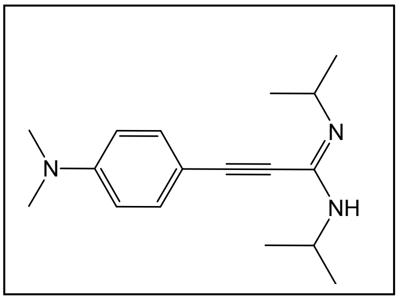

3d: FT-IR (cm^{-1}) = NH 3391, C≡C 2223, C=N 1563. ^{1}H NMR (C_6D_6) δ (ppm) = 1.02 (d, *J* = 6.4 Hz, 6H, CH_3), 1.46 (d, *J* = 6.2 Hz, 6H, CH_3), 2.34 (s, 6H, $N(CH_3)_2$), 3.83 (bs, 1H, NH), 4.28-4.45 (m, 2H, CH), 6.32-6.35 (m, 2Harom), 7.49-7.52 (m, 2Harom). $^{13}C\{^{1}H\}$ NMR (C_6D_6) δ (ppm) = 22.8 (2C), 25.6 (2C), 39.6 (2C), 42.6, 53.1, 79.5, 92.3, 108.7, 112.1 (2C), 133.5 (2C), 141.1, 151.0. Elem. anal. calc for [$C_{17}H_{25}N_3$]: C, 75.23; H, 9.28; N, 15.48. Found: C, 75.35; H, 9.33; N, 15.52.

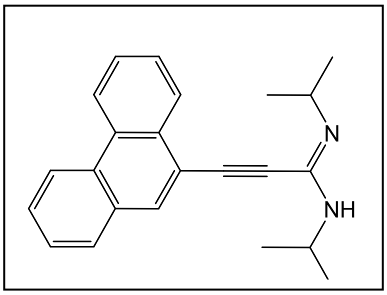

3e: FT-IR (cm^{-1}) = NH 3378, C≡C 2215, C=N 1565. ^{1}H NMR (C_6D_6) δ (ppm) = 1.06 (d, *J* = 6.5 Hz, 6H, CH_3), 1.49 (d, *J* = 6.1 Hz, 6H, CH_3), 3.91 (bs, 1H, NH), 4.33-4.37 (m, 1H, CH), 4.44-4.49 (m, 21H, CH), 7.27-7.30 (m, 1Harom), 7.34-7.41 (m, 2Harom), 7.44-7.51 (m, 2Harom), 7.96 (s, 1Harom), 8.33 (d, *J* = 8.3 Hz, 1Harom), 8.39 (d, *J* = 8.2 Hz, 1Harom), 8.65 (d, *J* = 8.1 Hz, 1Harom). $^{13}C\{^{1}H\}$ NMR (C_6D_6) δ (ppm) = 24.2 (4C), 48.2 (2C), 85.1, 88.9, 118.6, 123.0, 123.4, 127.1, 127.3, 127.6, 127.7, 128.2, 129.1, 130.6, 131.1, 131.3, 131.4, 133.6, 140.4. Elem. anal. calc for [$C_{23}H_{24}N_2$]: C, 84.11; H, 7.37; N, 8.53. Found: C, 84.01; H, 7.25; N, 8.61.

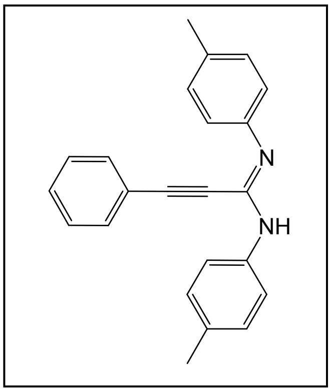

5a: FT-IR (cm^{-1}) = NH 3378, C≡C 2220, C=N 1570. ^{1}H NMR (C_6D_6) δ (ppm) = 2.13 (s, 6H, CH_3), 6.85-6.89 (m, 3Harom), 7.01-7.03 (m, 4Harom), 7.20-7.22 (m, 2Harom), 7.37-7.39 (m, 4Harom). $^{13}C\{^{1}H\}$ NMR (C_6D_6) δ (ppm) = 20.9 (2C), 81.7, 92.2, 121.5, 121.7 (4C), 128.6 (2C), 129.5 (4C), 129.7 (2C), 132.3 (2C), 132.5 (2C), 139.5, 143.5. Elem. anal. calc for [$C_{23}H_{20}N_2$]: C, 85.15; H, 6.21; N, 8.63. Found: C, 85.10; H, 6.17; N, 8.72.

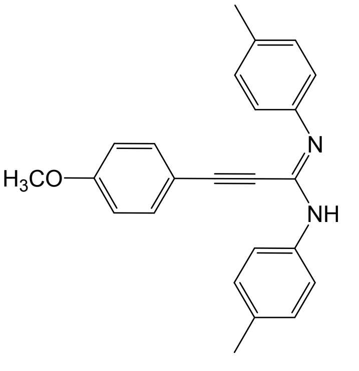

5b: FT-IR (cm^{-1}) = NH 3401, C≡C 2215, C=N 1560. ^{1}H NMR (C_6D_6) δ (ppm) = 2.15 (s, 6H, CH_3), 3.11 (s, 3H, CH_3O), 6.46 (d, *J* = 8.9 Hz, 2Harom), 7.04 (d, *J* = 8.2 Hz, 4Harom), 7.18 (d, *J* = 8.9 Hz, 2Harom), 7.42 (m, *J* = 8.1 Hz, 4Harom). ^{13}C{^{1}H} NMR (C_6D_6) δ (ppm) = 20.9 (2C), 54.7, 80.9, 92.9, 113.4, 114.5 (2C), 121.8 (4C), 129.5 (4C), 132.4 (2C), 134.1 (2C), 139.9, 161.1. Elem. anal. calc for [$C_{24}H_{22}N_2O$]: C, 81.33; H, 6.26; N, 7.90. Found: C, 81.44; H, 6.32; N, 7.96.

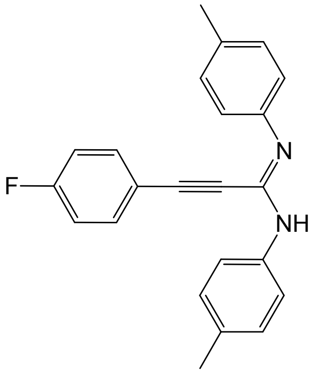

5c: FT-IR (cm^{-1}) = NH 3399, C≡C 2223, C=N 1566. ^{1}H NMR (C_6D_6) δ (ppm) = 2.14 (s, 6H, CH_3), 6.44-6.48 (m, 2Harom), 6.93-6.97 (m, 2Harom), 7.02 (d, *J* = 8.1 Hz, 4Harom), 7.36 (d, *J* = 8.3 Hz, 4Harom). ^{13}C{^{1}H} NMR (C_6D_6) δ (ppm) = 20.9 (2C), 81.4, 91.4, 115.8 (2C), 116.1 (2C), 117.4, 121.8 (2C), 129.5 (4C), 132.7 (2C), 134.5 (2), 139.6, 143.3, 162.2, 164.7. ^{19}F{^{1}H} NMR (C_6D_6) δ (ppm) = -108.24. Elem. anal. calc for [$C_{23}H_{19}FN_2$]: C, 80.68; H, 5.59; N, 8.18. Found: C, 80.73; H, 5.68; N, 8.29.

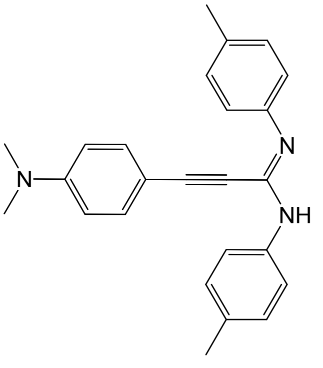

5d: FT-IR (cm^{-1}) = NH 3378, C≡C 2220, C=N 1570. ^{1}H NMR (C_6D_6) δ (ppm) = 2.16 (s, 6H, CH_3), 2.29 (s, 6H, $N(CH_3)_2$), 6.20 (d, *J* = 8.9 Hz, 2Harom), 7.05 (d, *J* = 8.1 Hz, 4Harom), 7.29 (d, *J* = 8.7 Hz, 2Harom), 7.45 (d, *J* = 8.1 Hz, 4Harom). ^{13}C{^{1}H} NMR (C_6D_6) δ (ppm) = 20.9 (2C), 39.4 (2C), 80.7, 94.9, 107.7 (2C), 111.9 (2C), 121.8 (4C), 129.4 (4C), 132.1 (2C), 133.8 (2C), 140.4, 143.9, 151.0. Elem. anal. calc for [$C_{25}H_{25}N_3$]: C, 81.71; H, 6.86; N, 11.43. Found: C, 81.79; H, 6.93; N, 11.50.

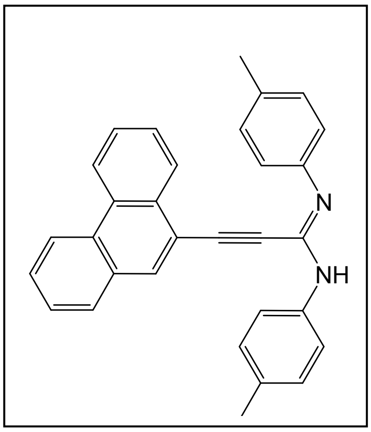

5e: FT-IR (cm^{-1}) = NH 3384, C≡C 2218, C=N 1571. ^1H NMR (C_6D_6) δ (ppm) = 2.18 (s, 6H, CH_3), 7.07 (d, J = 8.0 Hz, 4Harom), 7.23-7.26 (m, 1Harom), 7.31-7.34 (m, 1Harom), 7.39-7.46 (m, 7Harom), 7.78 (s, 1Harom), 7.87-7.88 (m, 1Harom), 8.28-8.29 (m, 1Harom), 8.31-8.33 (m, 1Harom). $^{13}C\{^1H\}$ NMR (C_6D_6) δ (ppm) = 20.9 (2C), 85.8, 90.8, 118.2, 121.6 (2C), 123.0 (2C), 127.2, 127.4, 127.5, 127.6, 127.9, 128.2, 129.1, 129.8 (4C), 130.4, 131.2, 131.3, 132.6, 133.9, 139.8. Elem. anal. calc for [$C_{31}H_{24}N_2$]: C, 87.70; H, 5.70; N, 6.60. Found: C, 87.73; H, 5.74; N, 6.69.

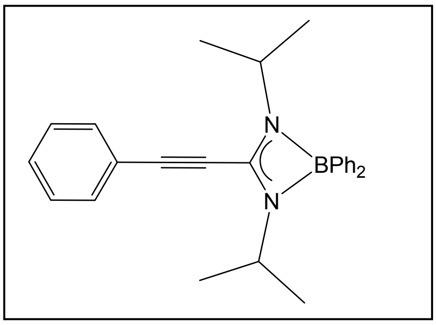

4a: FT-IR (cm^{-1}) = C≡C 2222, C=N 1638. ^1H NMR (C_6D_6) δ (ppm) = 1.09 (d, J = 6.6 Hz, 12H, CH_3), 3.85-3.94 (m, 2H, CH), 6.87-6.98 (m, 3Harom), 7.27-7.41 (m, 8Harom), 7.85-7.87 (m, 4Harom). $^{13}C\{^1H\}$ NMR (C_6D_6) δ (ppm) = 23.5 (4C), 47.9 (2C), 76.1, 97.6, 120.4, 127.0 (2C), 127.7 (4C), 128.9 (2C), 130.6, 132.4 (2C), 134.4 (4C), 149.9. $^{11}B\{^1H\}$ NMR (C_6D_6) δ (ppm) = 9.73. Elem. anal. calc for [$C_{27}H_{29}BN_2$]: C, 82.65; H, 7.45; N, 7.14. Found: C, 82.71; H, 7.49; N, 7.25. m.p. 66–69 °C.

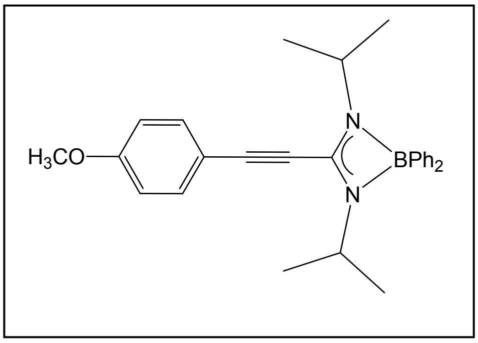

4b: FT-IR (cm^{-1}) = C≡C 2219, C=N 1637. ^1H NMR (C_6D_6) δ (ppm) = 1.11 (d, J = 6.6 Hz, 12H, CH_3), 3.13 (s, 3H, CH_3O), 3.91-3.98 (m, 2H, CH), 6.52-6.54 (m, 2Harom), 7.27-7.34 (m, 4Harom), 7.38-7.42 (m, 4Harom), 7.87-7.90 (m, 4Harom). $^{13}C\{^1H\}$ NMR (C_6D_6) δ (ppm) = 23.5 (4C), 47.8 (2C), 54.9, 75.5, 98.4, 112.2, 114.7 (2C), 127.0 (2C), 127.7 (4C), 134.3 (2C), 134.4 (4C), 138.9, 150.4, 161.8. $^{11}B\{^1H\}$ NMR (C_6D_6) δ (ppm) = 9.49. Elem. anal. calc for [$C_{28}H_{31}BN_2O$]: C, 79.62; H, 7.40; N, 6.63. Found: C, 79.71; H, 7.49; N, 6.74. m.p. 68–71 °C.

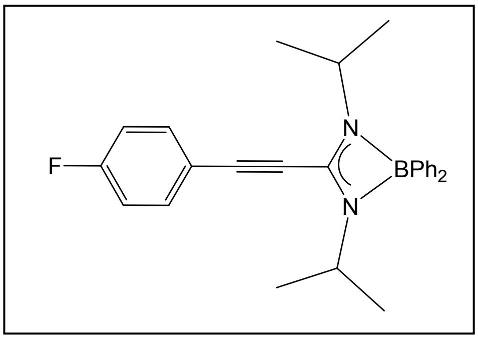

4c: FT-IR (cm^{-1}) = C≡C 2220, C=N 1637. ^{1}H NMR (C_6D_6) δ (ppm) = 1.09 (d, J = 6.7 Hz, 12H, CH_3), 3.83-3.93 (m, 2H, CH), 6.50-6.54 (m, 2Harom), 6.99-7.03 (m, 2Harom), 7.29-7.42 (m, 6Harom), 7.84-7.87 (m, 4Harom). $^{13}C\{^{1}H\}$ NMR (C_6D_6) δ (ppm) = 23.5 (4C), 47.9 (2C), 75.8, 96.5, 116.2, 116.4, 127.1 (2C), 134.4 (4C), 134.6, 134.7, 149.7, 162.7, 165.3. $^{19}F\{^{1}H\}$ NMR (C_6D_6) δ (ppm) = -106.53. $^{11}B\{^{1}H\}$ NMR (C_6D_6) δ (ppm) = 9.88. Elem. anal. calc for [$C_{27}H_{28}BFN_2$]: C, 79.03; H, 6.88; N, 6.83. Found: C, 79.11; H, 6.91; N, 6.90. m.p. 70–73 °C.

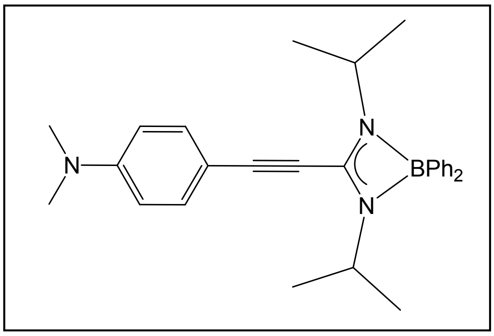

4d: FT-IR (cm^{-1}) = C≡C 2221, C=N 1638. ^{1}H NMR (C_6D_6) δ (ppm) = 1.14 (d, J = 6.6 Hz, 12H, CH_3), 2.30 (s, 6H, $N(CH_3)_2$), 3.96-4.03 (m, 2H, CH), 6.25-6.28 (m, 2Harom), 7.29-7.44 (m, 9Harom), 7.90-7.92 (m, 4Harom). $^{13}C\{^{1}H\}$ NMR (C_6D_6) δ (ppm) = 23.6 (4C), 39.3 (2C), 47.8 (2C), 75.5, 100.8, 106.4, 111.9 (2C), 126.9 (2C), 127.7 (4C), 134.1 (2C), 134.5 (4C), 151.2, 151.6. $^{11}B\{^{1}H\}$ NMR (C_6D_6) δ (ppm) = 9.39. Elem. anal. calc for [$C_{29}H_{34}BN_3$]: C, 80.00; H, 7.87; N, 9.65. Found: C, 80.08; H, 7.81; N, 9.75. m.p. 65–70 °C.

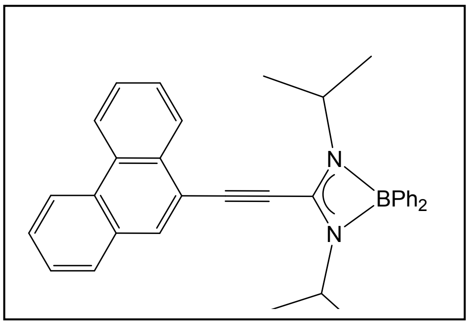

4e: FT-IR (cm^{-1}) = C≡C 2219, C=N 1637. ^{1}H NMR (C_6D_6) δ (ppm) = 1.18 (d, J = 6.6 Hz, 12H, CH_3), 4.03-4.11 (m, 2H, CH), 7.26-7.29 (m, 2Harom), 7.32-7.39 (m, 4Harom), 7.41-7.46 (m, 6Harom), 7.93 (d, J = 8.1 Hz, 4Harom), 8.29 (d, J = 8.2 Hz, 1Harom), 8.35 (d, J = 8.1 Hz, 1Harom), 8.51-8.53 (m, 1Harom). $^{13}C\{^{1}H\}$ NMR (C_6D_6) δ (ppm) = 23.7 (4C), 48.1 (2C), 80.0, 96.5, 116.9, 123.0, 123.1, 123.5, 126.5, 127.1, 127.5, 127.7, 127.9, 128.9, 129.3, 130.6, 130.8, 130.9, 131.5, 131.6, 132.4, 132.5, 134.5, 135.1, 136.2, 146.0, 150.0. $^{11}B\{^{1}H\}$ NMR (C_6D_6) δ (ppm) = 10.01. Elem. anal. calc for

[$C_{35}H_{33}BN_2.C_5H_{12}$]: C, 85.09; H, 8.03; N, 4.96. Found: C, 85.00; H, 7.96; N, 5.01. m.p. 72–75 °C.

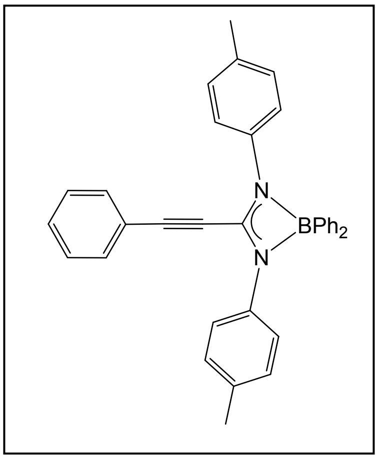

6a: FT-IR (cm^{-1}) = C≡C 2220, C=N 1639. ^{1}H NMR (C_6D_6) δ (ppm) = 2.00 (s, 6H, CH_3), 6.81-6.83 (m, 4Harom), 6.86- 6.95 (m, 3Harom), 7.22-7.26 (m, 2Harom), 7.29-7.35 (m, 6Harom), 7.52-7.54 (m, 4Harom), 7.94-7.96 (m, 4Harom). ^{13}C{^{1}H} NMR (C_6D_6) δ (ppm) = 20.9 (2C), 78.6, 99.8, 119.6 (4C), 119.9, 127.6 (2C), 128.2 (4C), 128.9 (2C), 130.1 (4C), 131.1 (2C), 132.7 (2C), 133.8 (2C), 134.2 (4C), 136.3, 137.9 (2C), 141.2. ^{11}B{^{1}H} NMR (C_6D_6) δ (ppm) = 9.60. Elem. anal. calc for [$C_{35}H_{29}BN_2$]: C, 86.07; H, 5.98; N, 5.74. Found: C, 86.14; H, 6.05; N, 5.82. m.p. 65–68 °C.

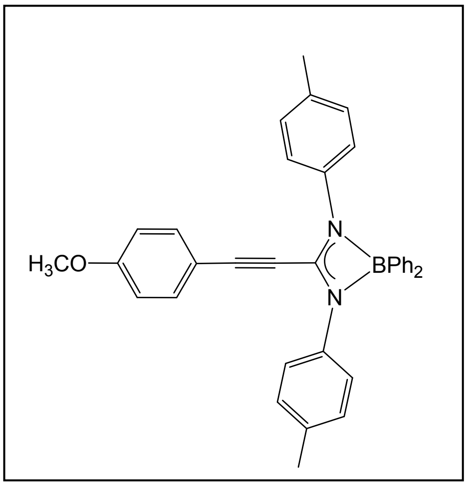

6b: FT-IR (cm^{-1}) = C≡C 2221, C=N 1639. ^{1}H NMR (C_6D_6) δ (ppm) = 2.02 (s, 6H, CH_3), 3.11 (s, 3H, CH_3O), 6.50-6.52 (m, 2Harom), 6.84- 6.86 (m, 4Harom), 7.23-7.27 (m, 2Harom), 7.29-7.35 (m, 6Harom), 7.55-7.58 (m, 4Harom), 7.96-7.98 (m, 4Harom). ^{13}C{^{1}H} NMR (C_6D_6) δ (ppm) = 20.9 (2C), 54.9, 78.2, 100.9, 111.7, 114.8 (2C), 119.6 (4C), 127.6 (2C), 127.9 (4C), 128.2 (2C), 130.0 (4C), 133.6 (2C), 134.2 (4C), 134.7 (2C), 138.1 (2C), 141.7, 162.2. ^{11}B{^{1}H} NMR (C_6D_6) δ (ppm) = 9.88. Elem. anal. calc for [$C_{36}H_{31}BN_2O$]: C, 83.40; H, 6.03; N, 5.40. Found: C, 83.52; H, 6.11; N, 5.45. m.p. 69–73 °C.

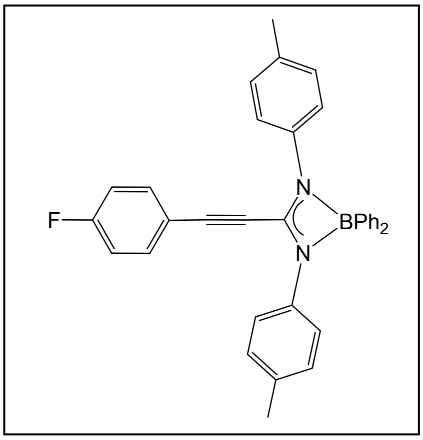

6c: FT-IR (cm^{-1}) = C≡C 2219, C=N 1640. ^{1}H NMR (C_6D_6) δ (ppm) = 2.02 (s, 6H, CH_3), 6.49-6.53 (m, 2Harom), 6.83- 6.85 (m, 4Harom), 7.02-7.05 (m, 2Harom), 7.24-7.26 (m, 2Harom), 7.32-7.36 (m, 4Harom), 7.48-7.50 (m, 4Harom), 7.94-7.96 (m, 4Harom). $^{13}C\{^{1}H\}$ NMR (C_6D_6) δ (ppm) = 20.9 (2C), 78.3, 98.6, 116.3 (2C), 116.5 (2C), 119.6 (4C), 127.7 (4C), 129.3 (2C), 130.1 (4C), 133.9 (2C), 134.1 (4C), 134.9 (2C), 137.8 (2C), 141.1, 163.3, 165.3. $^{19}F\{^{1}H\}$ NMR (C_6D_6) δ (ppm) = -105.36. $^{11}B\{^{1}H\}$ NMR (C_6D_6) δ (ppm) = 9.96. Elem. anal. calc for [$C_{35}H_{28}BFN_2$]: C, 83.01; H, 5.57; N, 5.53. Found: C, 82.91; H, 5.45; N, 5.59. m.p. 69–73 °C.

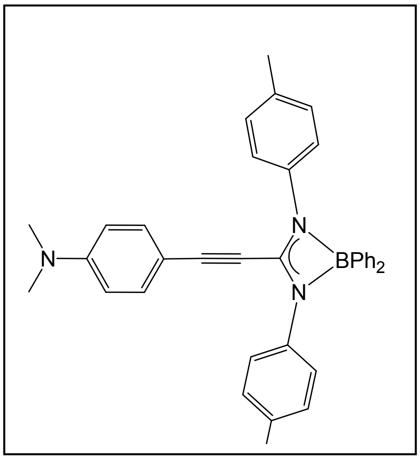

6d: FT-IR (cm^{-1}) = C≡C 2223, C=N 1641. ^{1}H NMR (C_6D_6) δ (ppm) = 2.03 (s, 6H, CH_3), 2.27 (s, 6H, $N(CH_3)_2$), 6.21-6.24 (m, 2Harom), 6.86- 6.88 (m, 4Harom), 7.23-7.27 (m, 2Harom), 7.32-7.35 (m, 4Harom), 7.45-7.47 (m, 2Harom), 7.63-7.65 (m, 4Harom), 7.98-8.01 (m, 4Harom). $^{13}C\{^{1}H\}$ NMR (C_6D_6) δ (ppm) = 20.9 (2C), 39.2 (2C), 78.8, 86.2, 103.9, 105.8 (2C), 111.9 (2C), 119.5 (4C), 127.4 (2C), 128.6 (4C), 129.9 (4C), 133.2 (2C), 134.3 (4C), 134.5 (2C), 138.4 (2C), 142.3, 151.9. $^{11}B\{^{1}H\}$ NMR (C_6D_6) δ (ppm) = 10.49. Elem. anal. calc for [$C_{37}H_{34}N_3.CH_2Cl_2$]: C, 74.04; H, 5.89; N, 6.82. Found: C, 73.90; H, 5.79; N, 6.77. m.p. 68–70 °C.

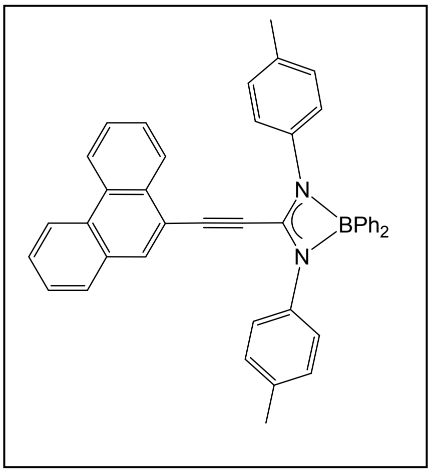

6e: FT-IR (cm^{-1}) = C≡C 2221, C=N 1641. ^{1}H NMR (C_6D_6) δ (ppm) = 2.03 (s, 6H, CH_3), 6.87-6.89 (m, 5Harom), 7.26-7.28 (m, 3Harom), 7.35-7.45 (m, 7Harom), 7.66-7.67 (m, 4Harom), 7.90 (s, 1Harom), 8.01-8.03 (m, 4Harom), 8.27-8.29 (m, 1Harom), 8.33-8.34 (m, 1Harom), 8.47-8.48 (m, 1Harom). ^{13}C{^{1}H} NMR (C_6D_6) δ (ppm) = 20.9 (2C), 82.3, 98.9, 116.7, 119.9 (4C), 123.1, 123.4, 126.9, 127.4, 127.5, 127.7 (2C), 127.9 (4C), 128.2 (2C), 129.2, 129.5, 129.8, 130.1 (4C), 130.5, 130.8, 130.9, 131.8, 133.9 (2C), 134.2 (4C), 134.4, 136.1, 138.0, 141.4. ^{11}B{^{1}H} NMR (C_6D_6) δ (ppm) = 9.05. Elem. anal. calc for [$C_{43}H_{33}BN_2$]: C, 87.75; H, 5.65; N, 4.76. Found: C, 87.81; H, 5.67; N, 4.80. m.p. 70–72 °C.

3.- ^{1}H and ^{13}C NMR spectra

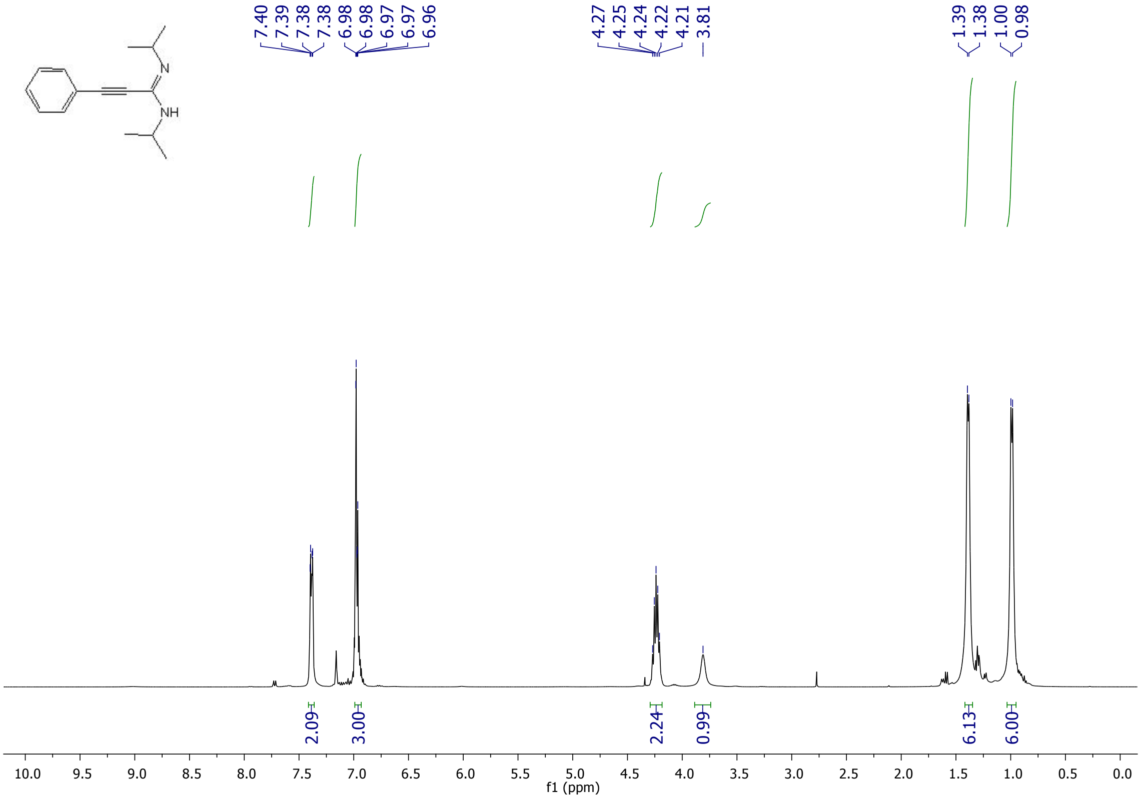

Figure S3. ^{1}H-NMR full chart for **3a** in C_6D_6.

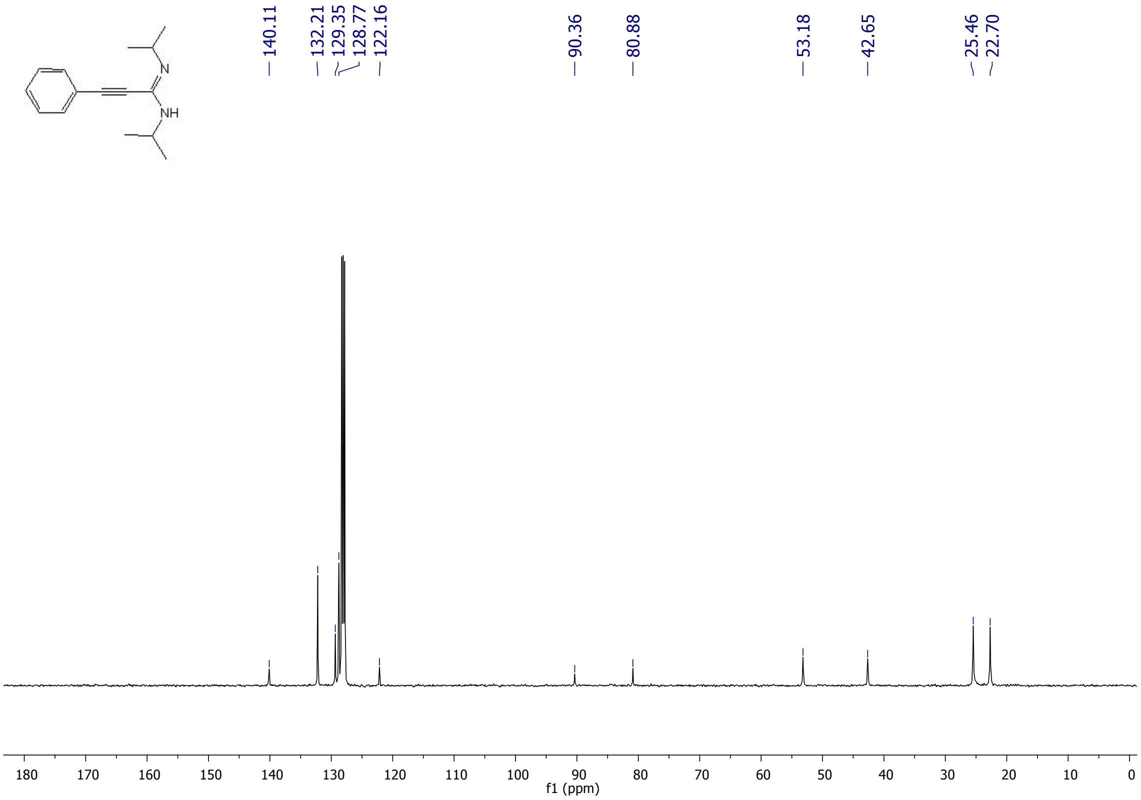

Figure S4. $^{13}C\{^{1}H\}$-NMR full chart for **3a** in C_6D_6.

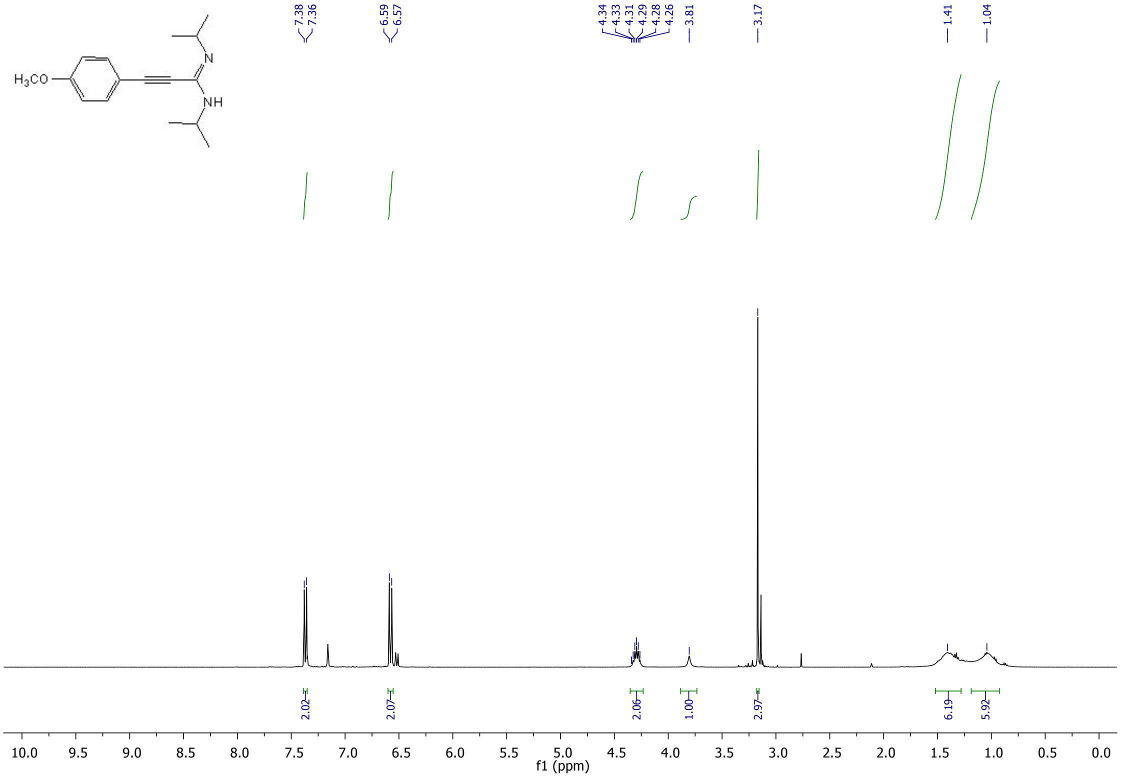

Figure S5. ^{1}H-NMR full chart for **3b** in C_6D_6.

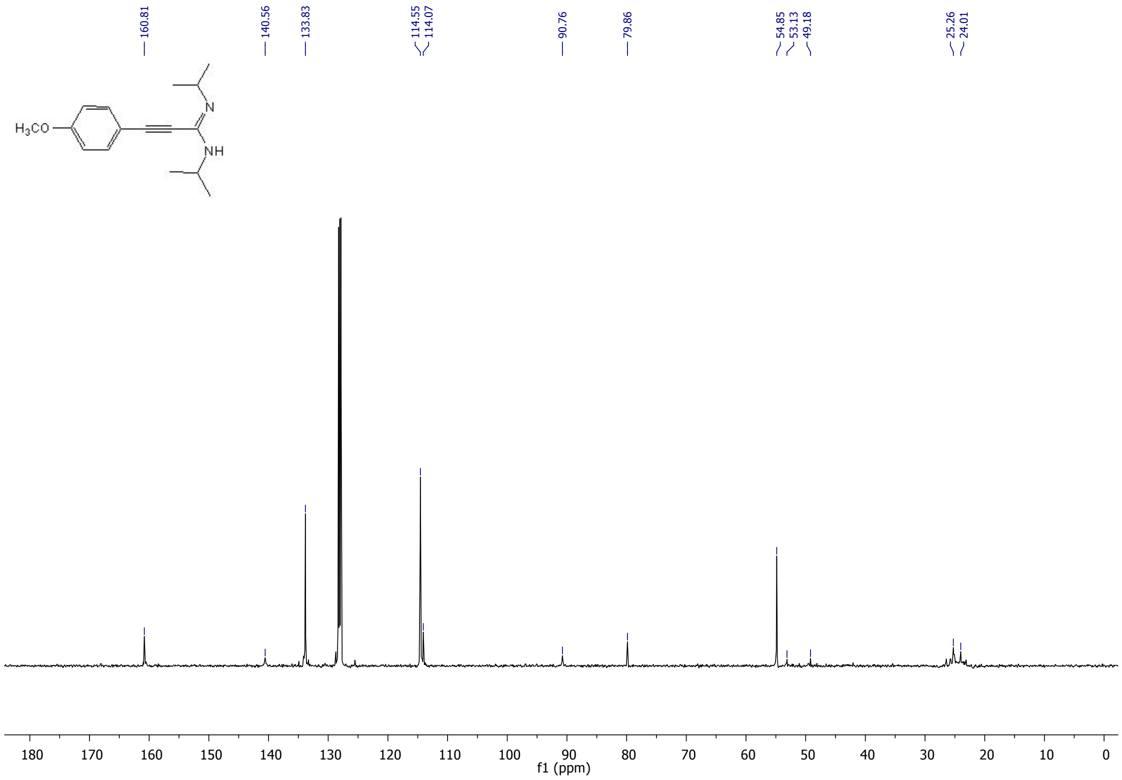

Figure S6. $^{13}C\{^{1}H\}$-NMR full chart for **3b** in C_6D_6.

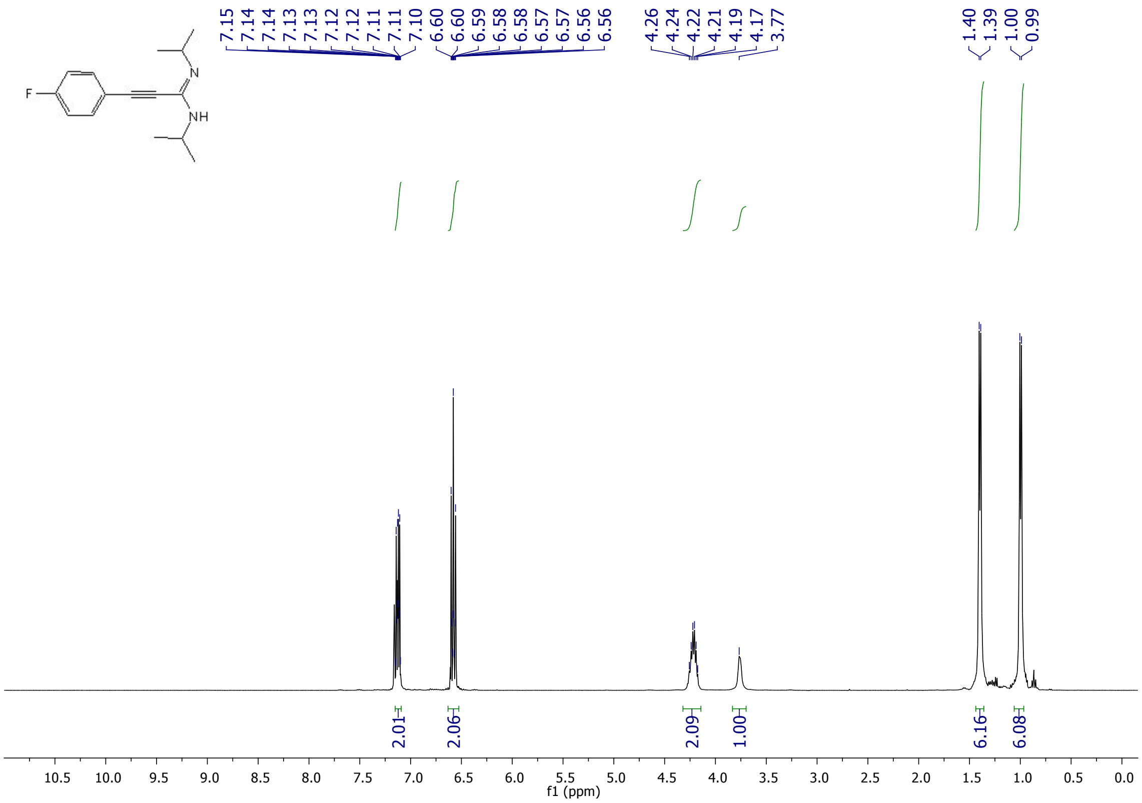

Figure S7. ^{1}H-NMR full chart for **3c** in C_6D_6.

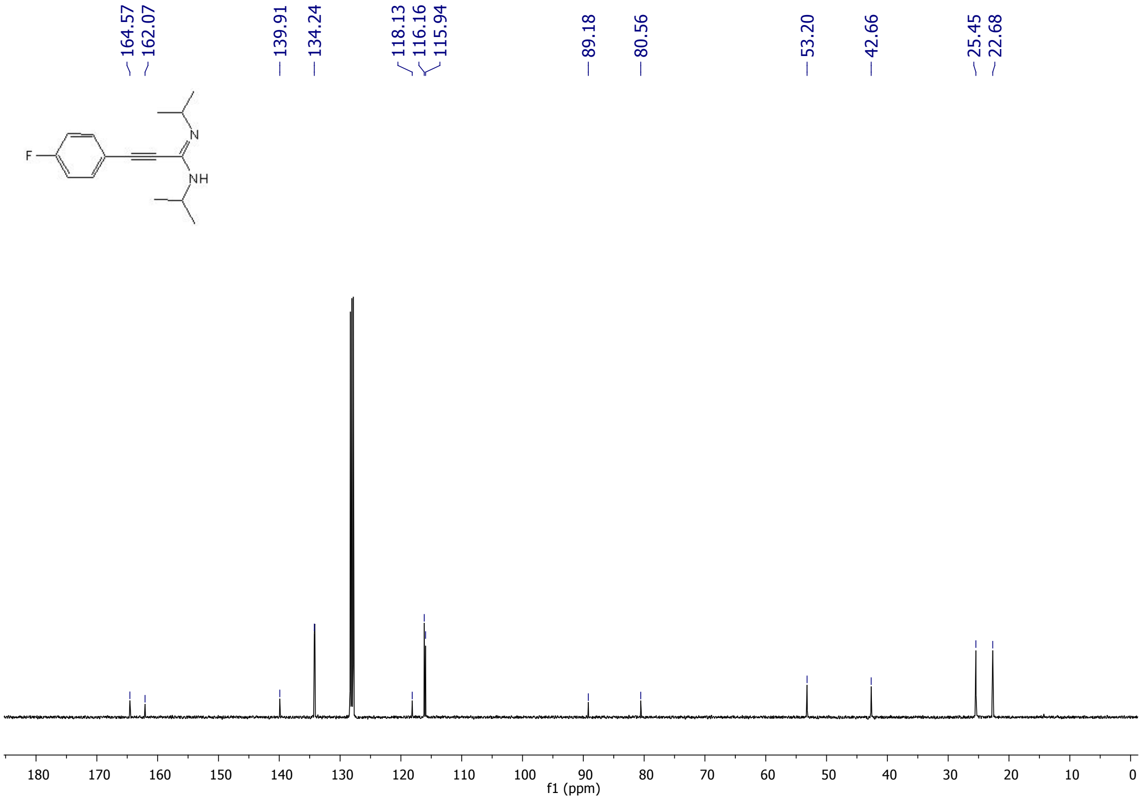

Figure S8. $^{13}C\{^1H\}$-NMR full chart for **3c** in C_6D_6.

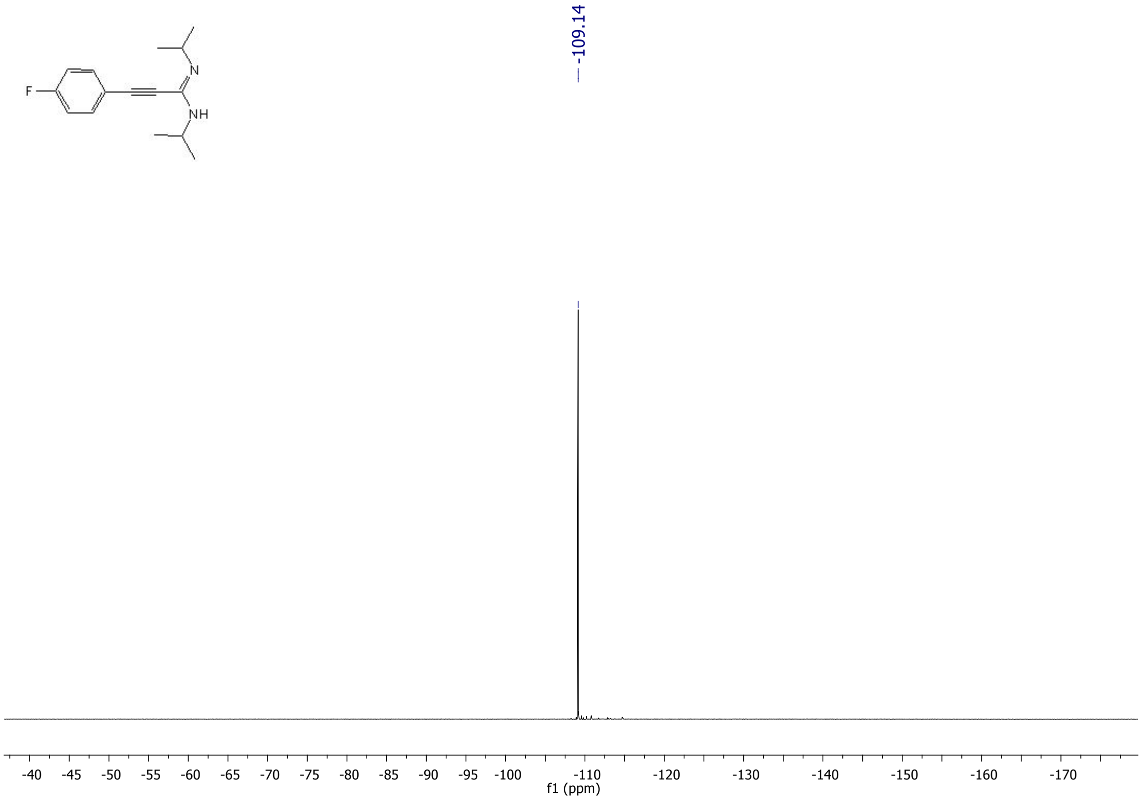

Figure S9. $^{19}F\{^1H\}$-NMR full chart for **3c** in C_6D_6.

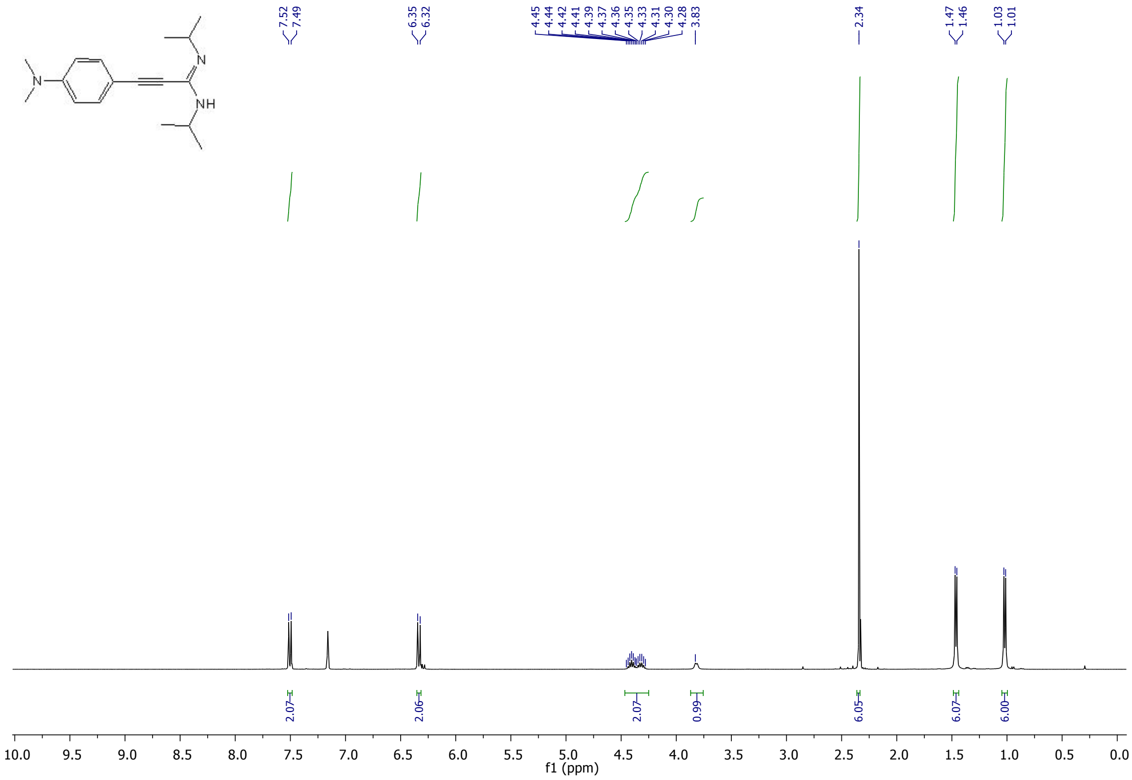

Figure S10. ^{1}H-NMR full chart for **3d** in C_6D_6.

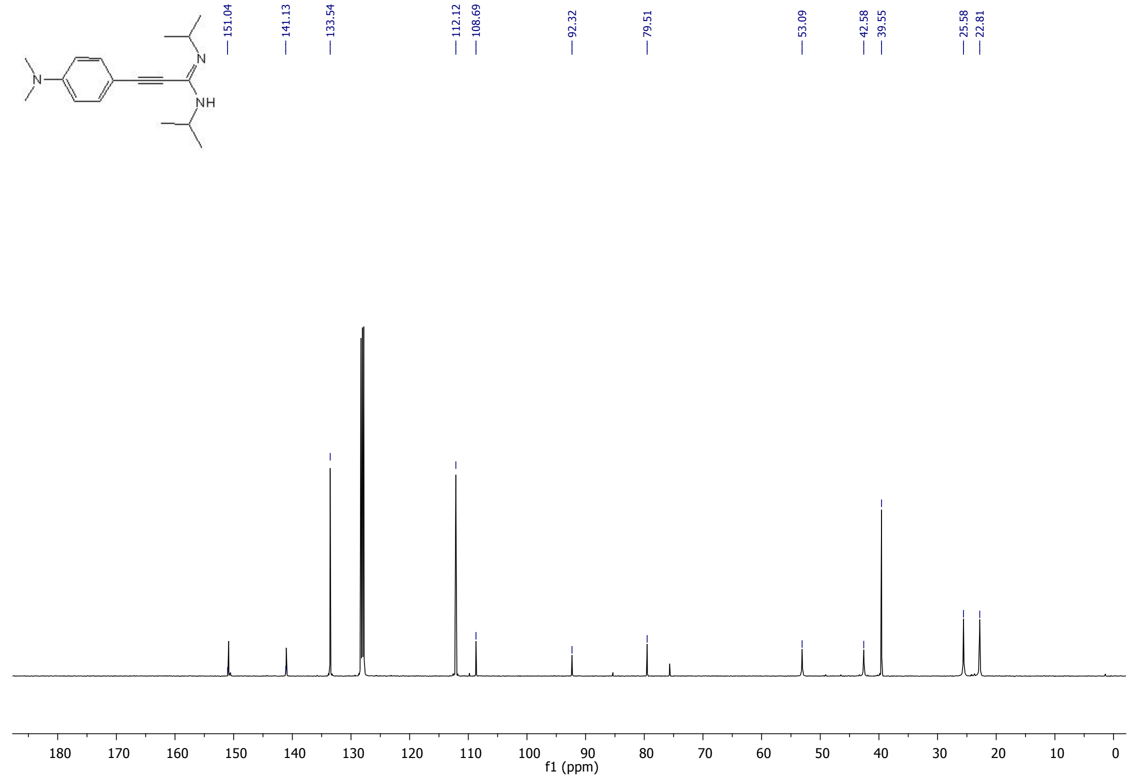

Figure S11. ^{13}C{^{1}H}-NMR full chart for **3d** in C_6D_6.

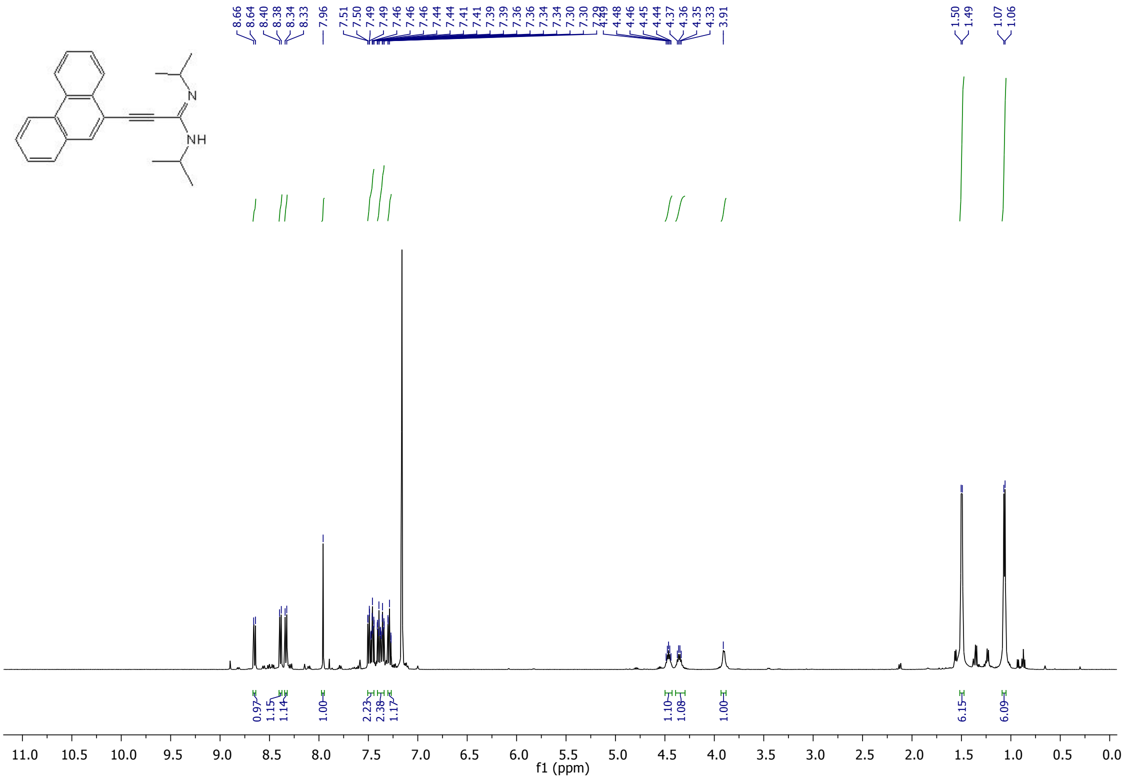

Figure S12. ^{1}H-NMR full chart for **3e** in C_6D_6.

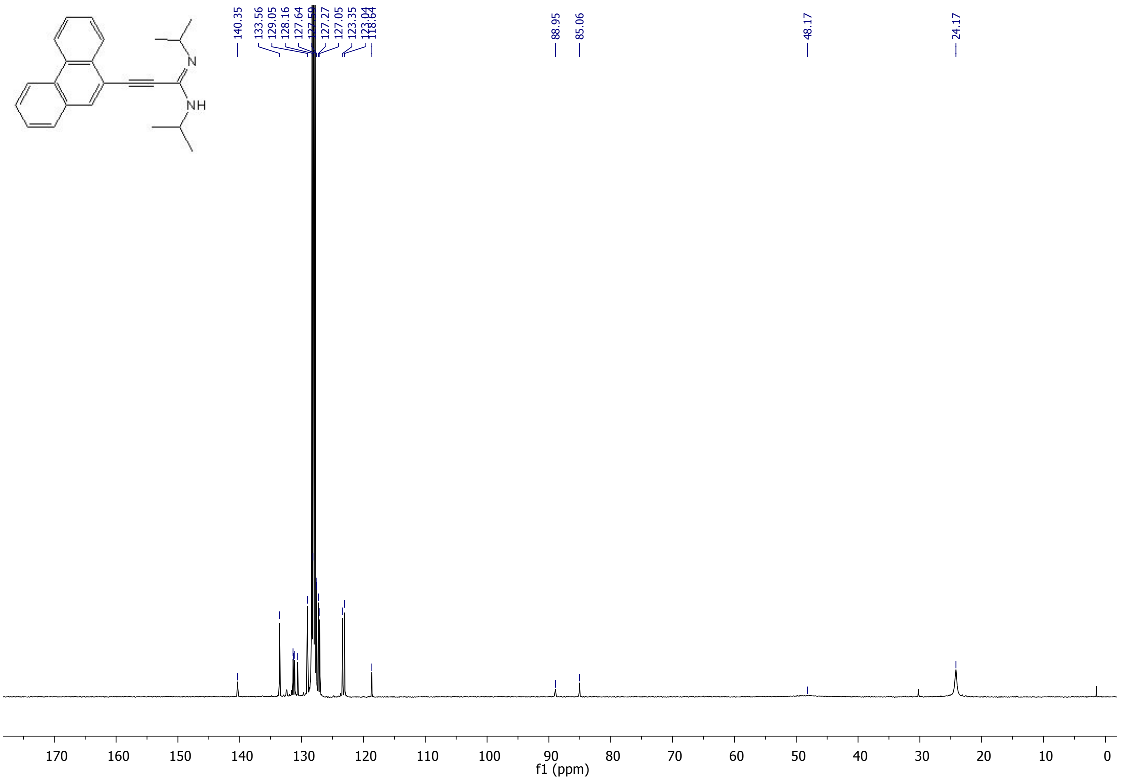

Figure S13. $^{13}C\{^{1}H\}$-NMR full chart for **3e** in C_6D_6.

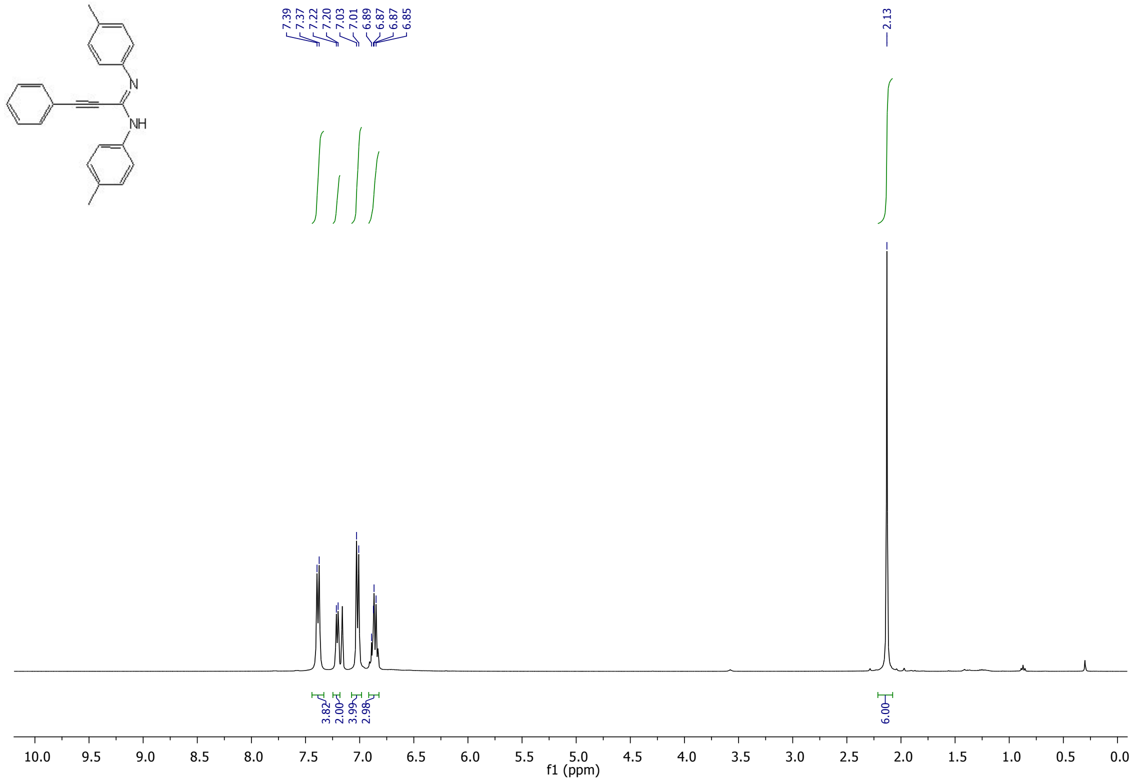

Figure S14. ^{1}H-NMR full chart for **5a** in C_6D_6.

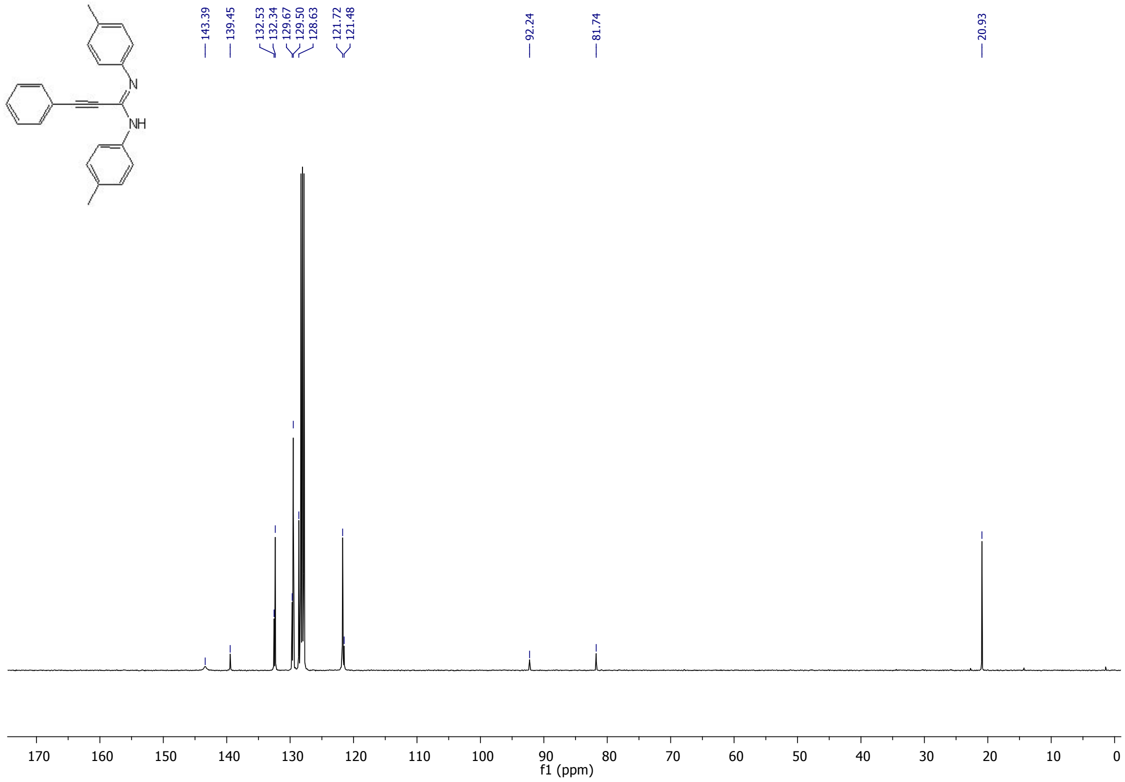

Figure S15. $^{13}C\{^{1}H\}$-NMR full chart for **5a** in C_6D_6.

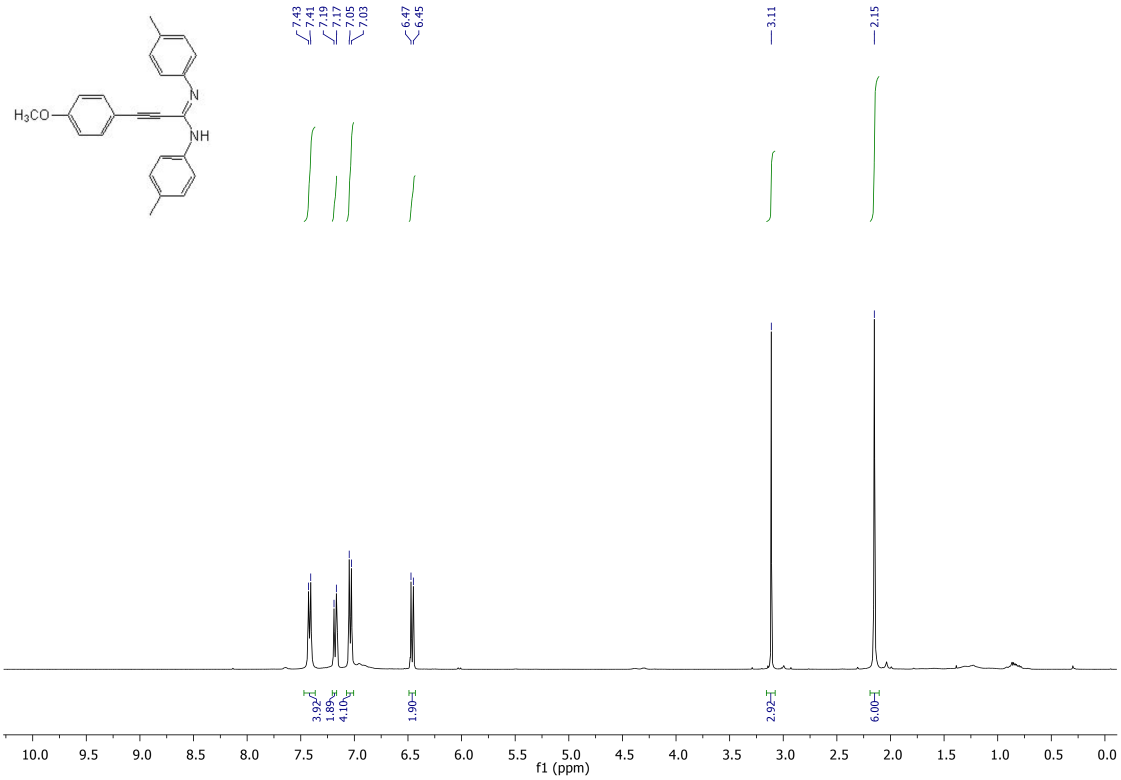

Figure S16. ^{1}H-NMR full chart for **5b** in C_6D_6.

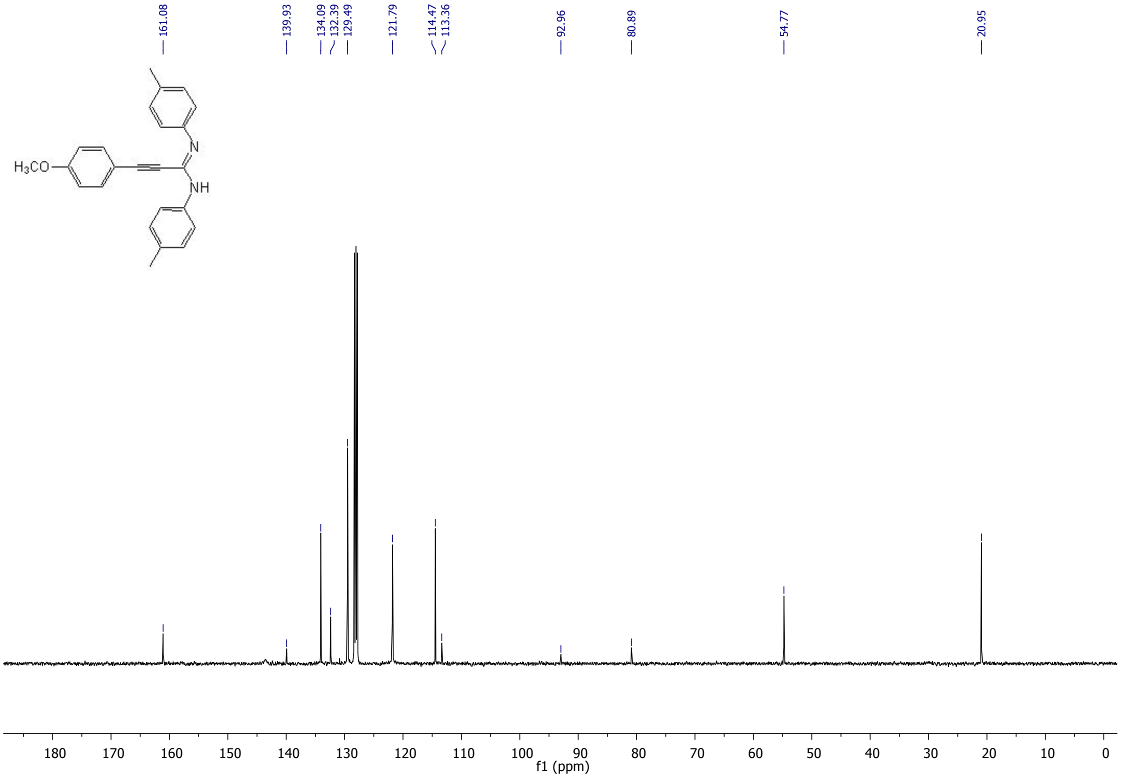

Figure S17. ^{13}C{^{1}H}-NMR full chart for **5b** in C_6D_6.

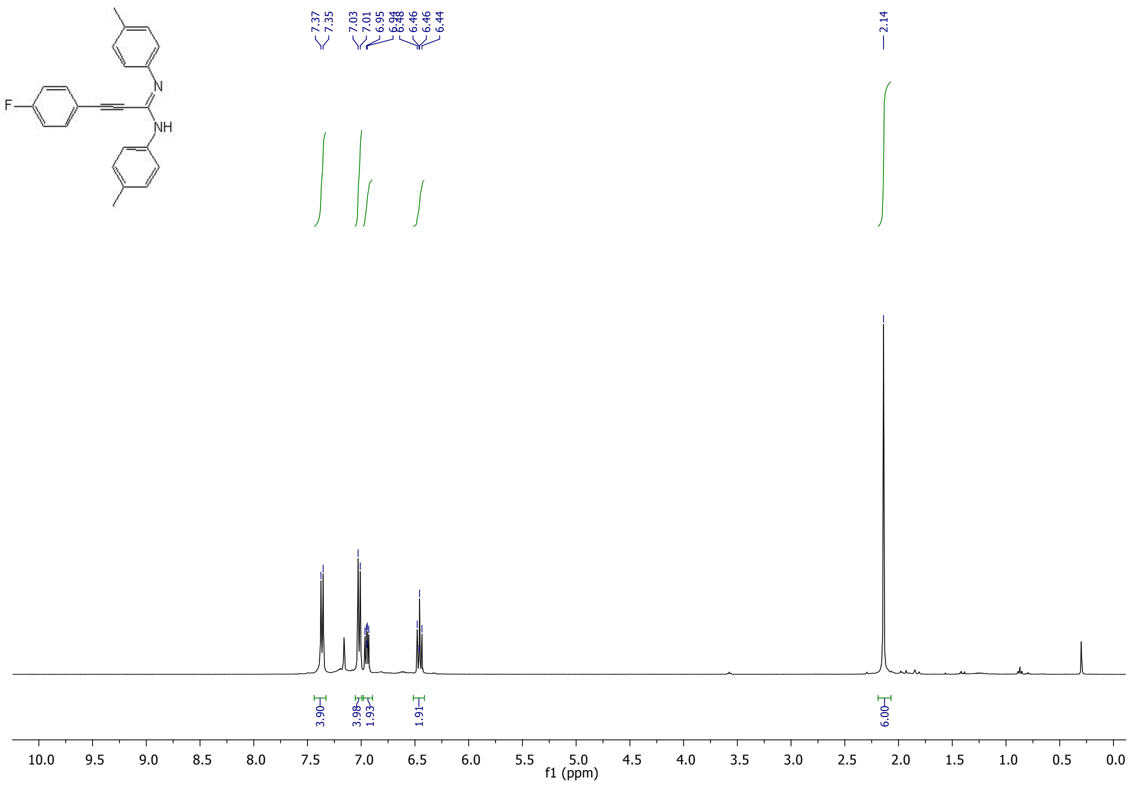

Figure S18. ^{1}H-NMR full chart for **5c** in C_6D_6.

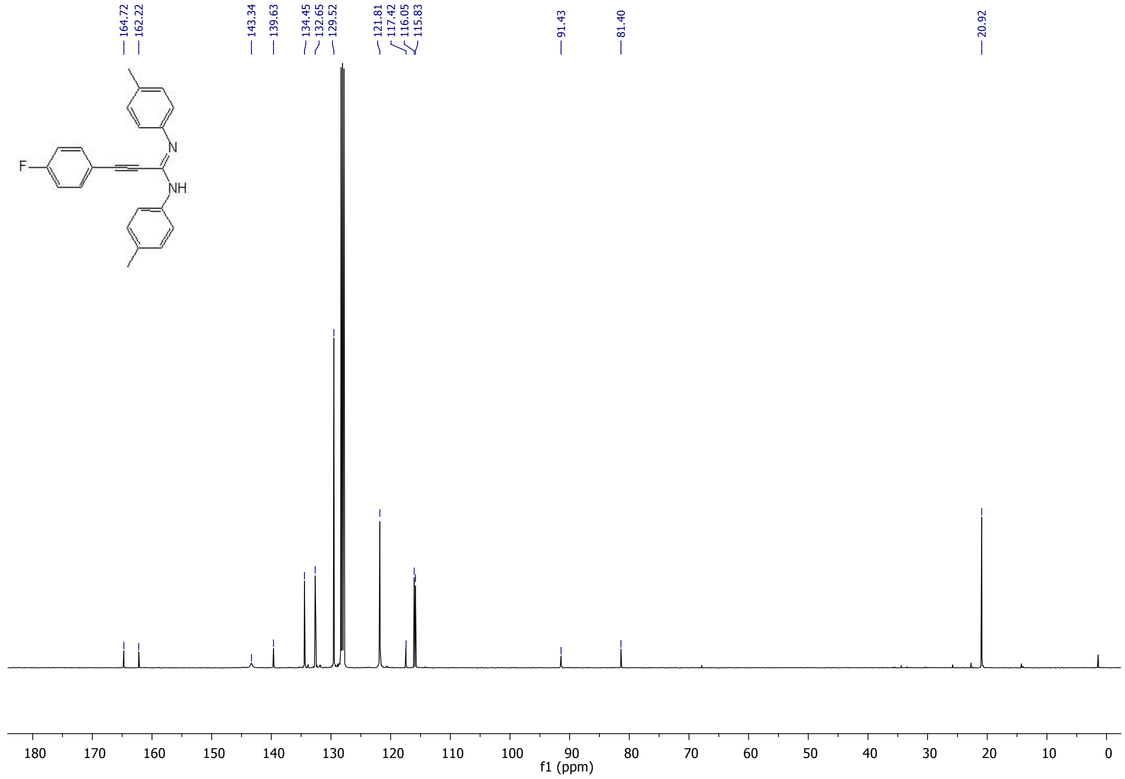

Figure S19. $^{13}C\{^{1}H\}$-NMR full chart for **5c** in C_6D_6.

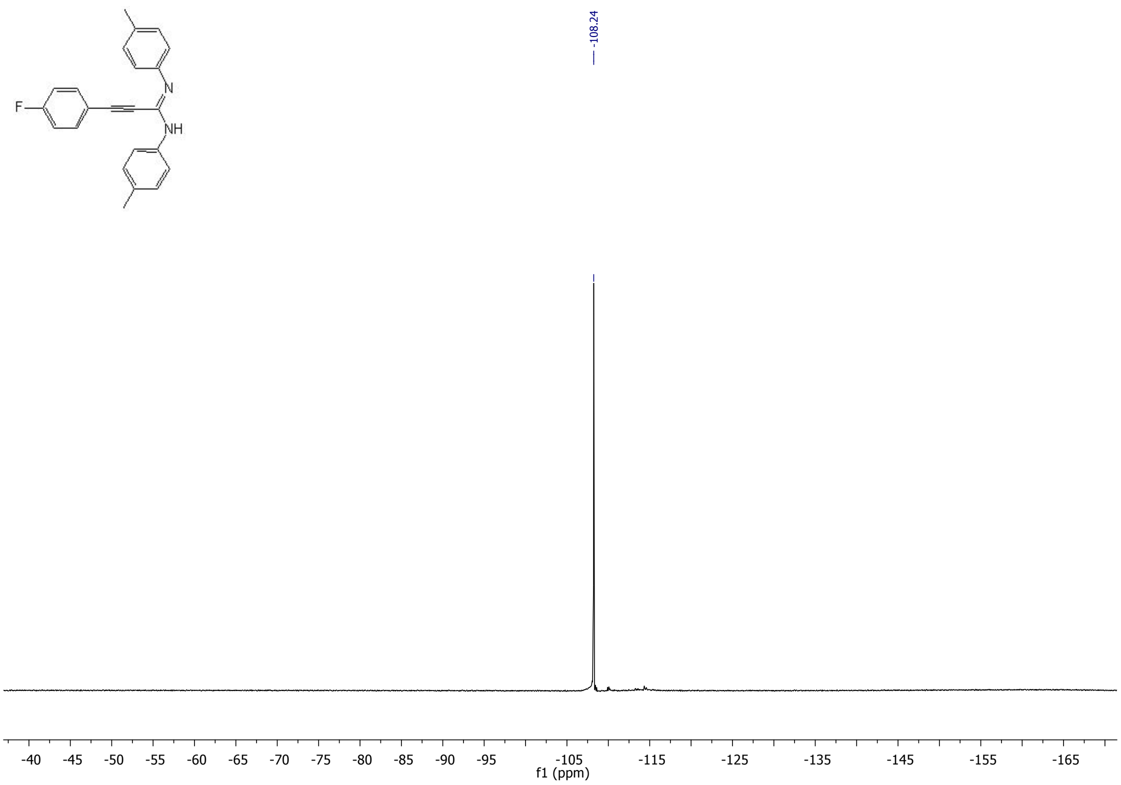

Figure S20. $^{19}F\{^{1}H\}$-NMR full chart for 5c in C_6D_6.

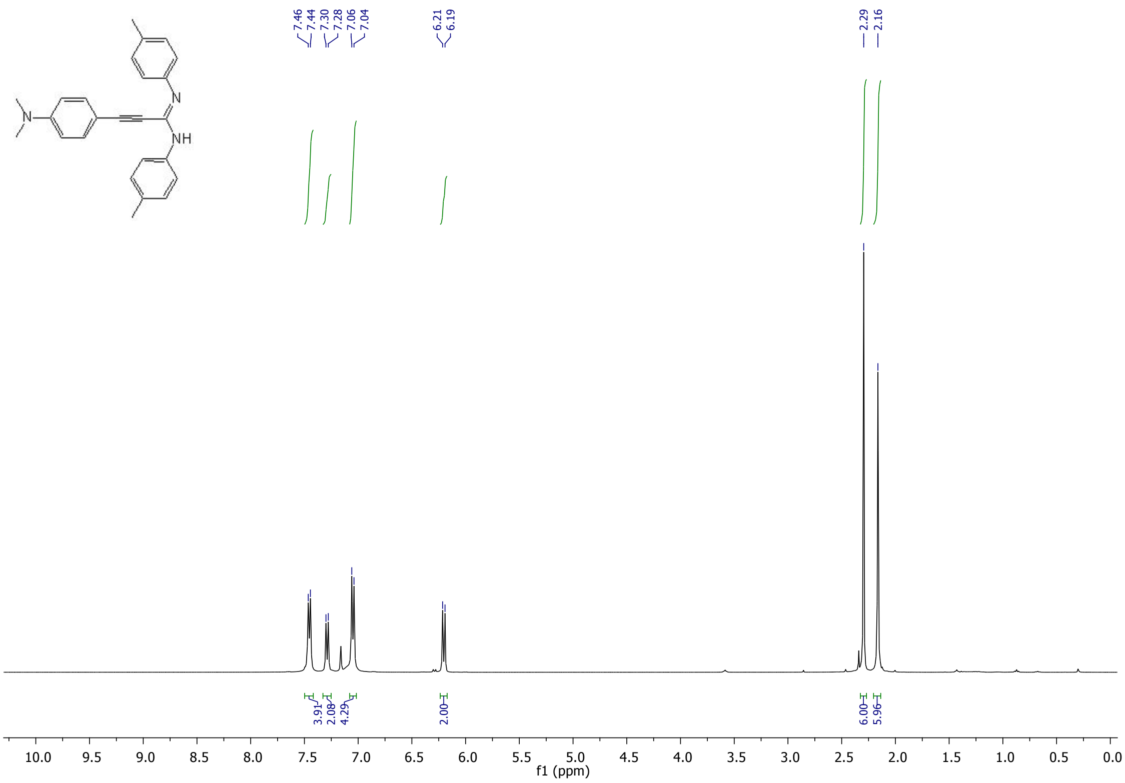

Figure S21. ^{1}H-NMR full chart for **5d** in C_6D_6.

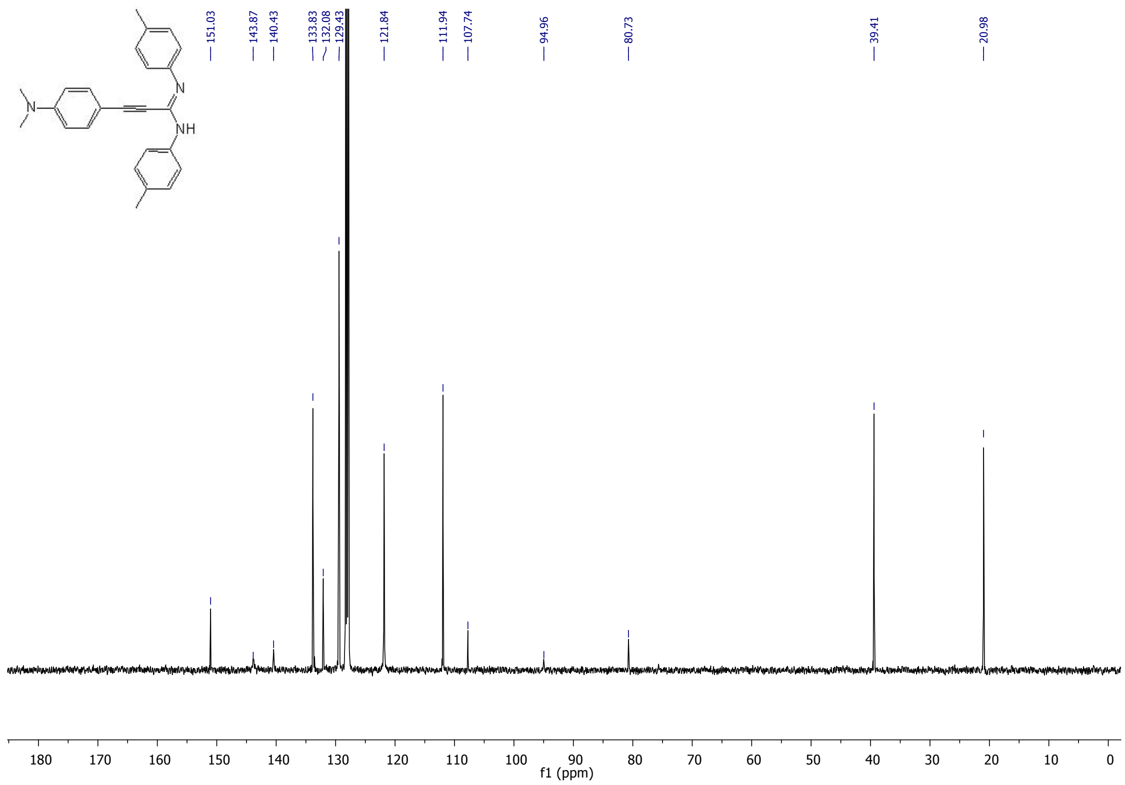

Figure S22. $^{13}C\{^{1}H\}$-NMR full chart for **5d** in C_6D_6.

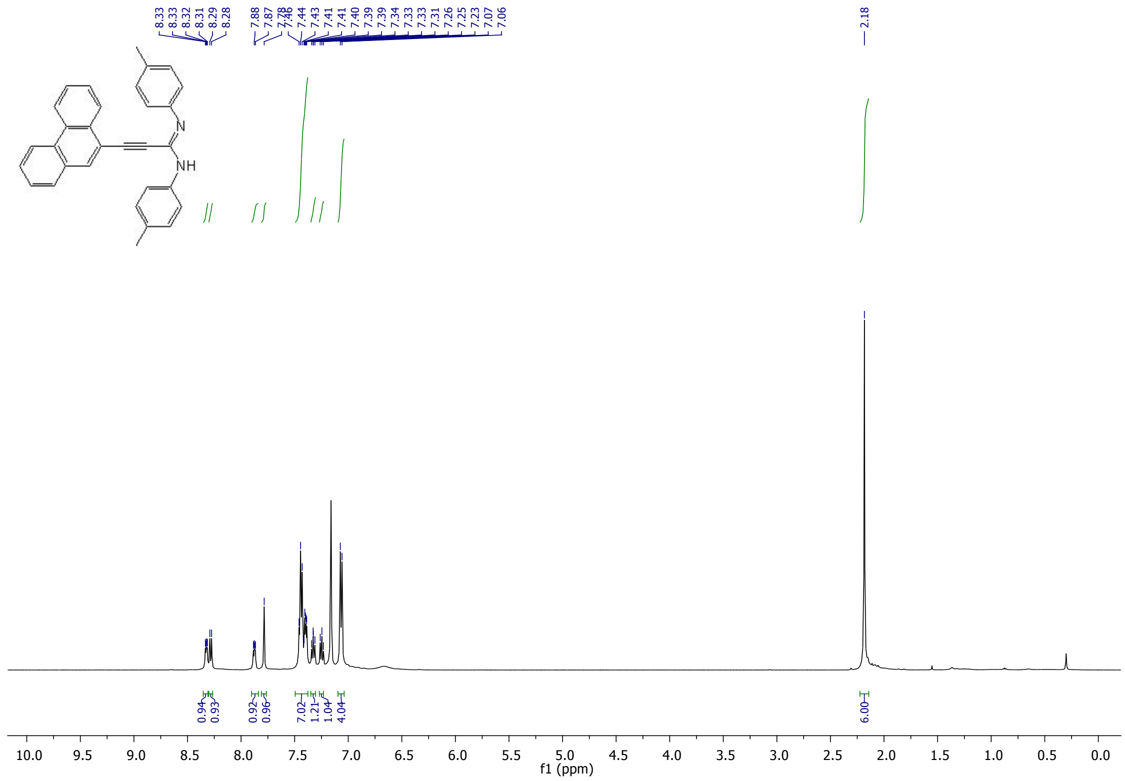

Figure S23. ^{1}H-NMR full chart for **5e** in C_6D_6.

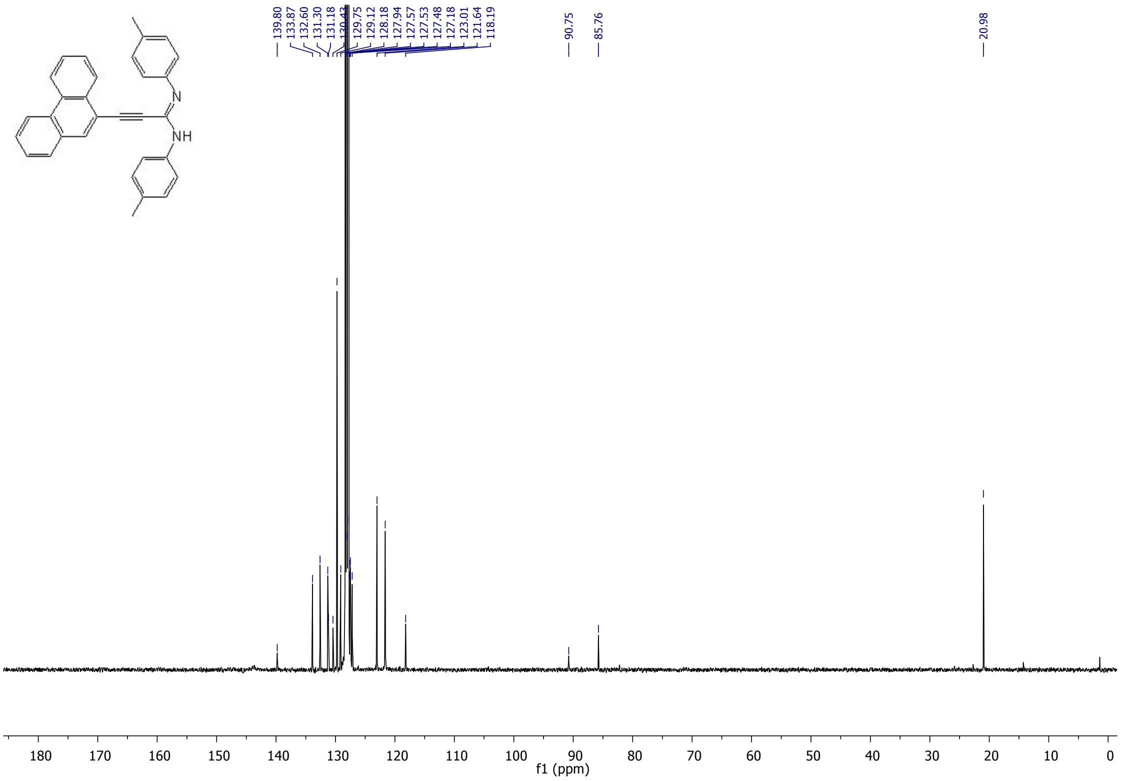

Figure S24. $^{13}C\{^1H\}$-NMR full chart for **5e** in C_6D_6.

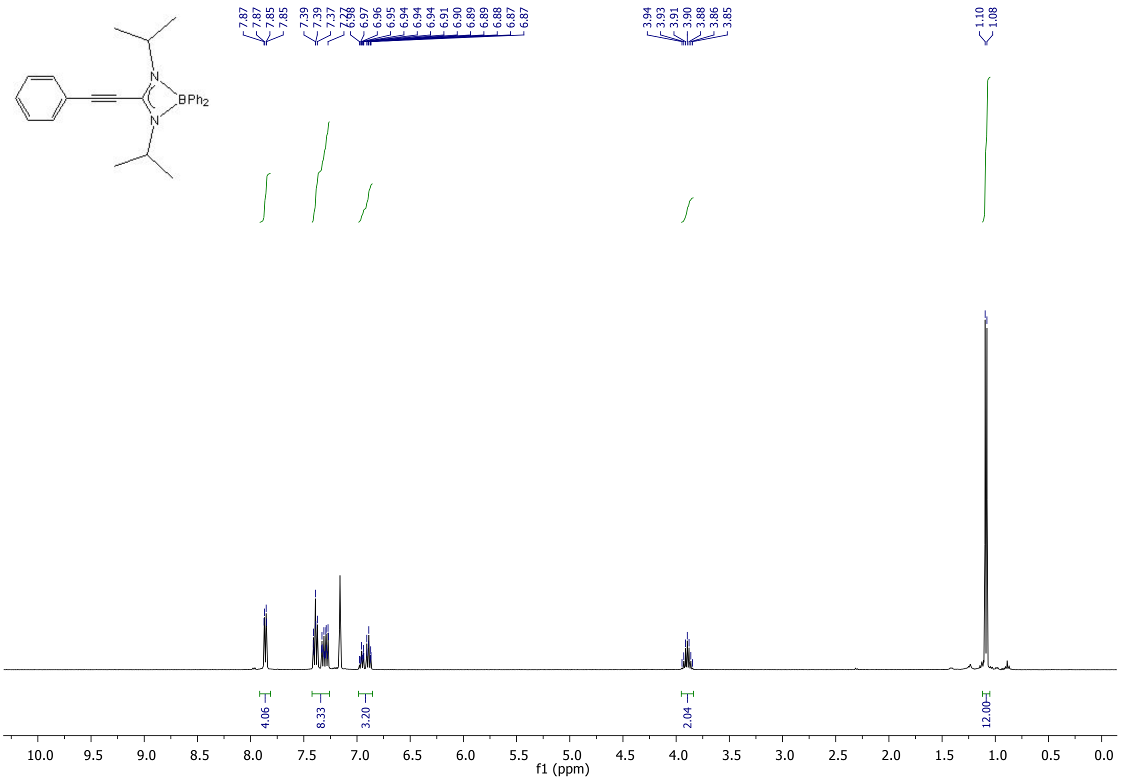

Figure S25. ^1H-NMR full chart for **4a** in C_6D_6.

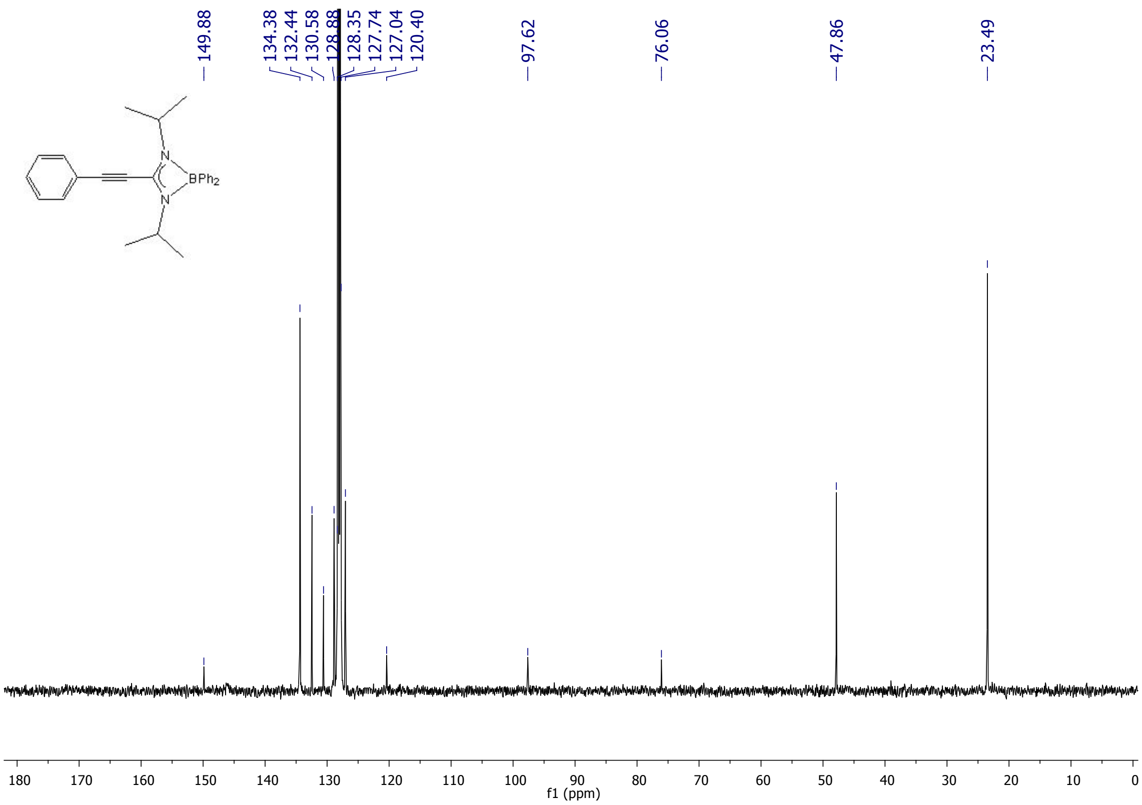

Figure S26. $^{13}C\{^{1}H\}$-NMR full chart for **4a** in C_6D_6.

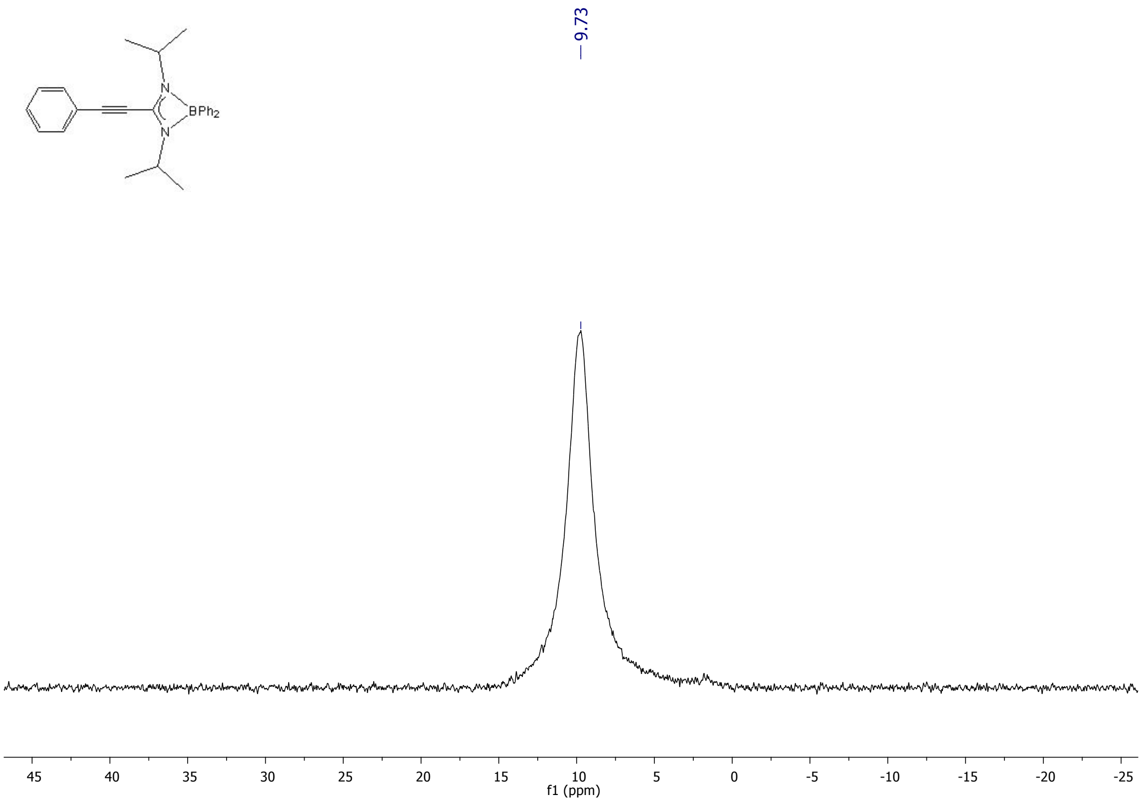

Figure S27. $^{11}B\{^{1}H\}$-NMR full chart for **4a** in C_6D_6.

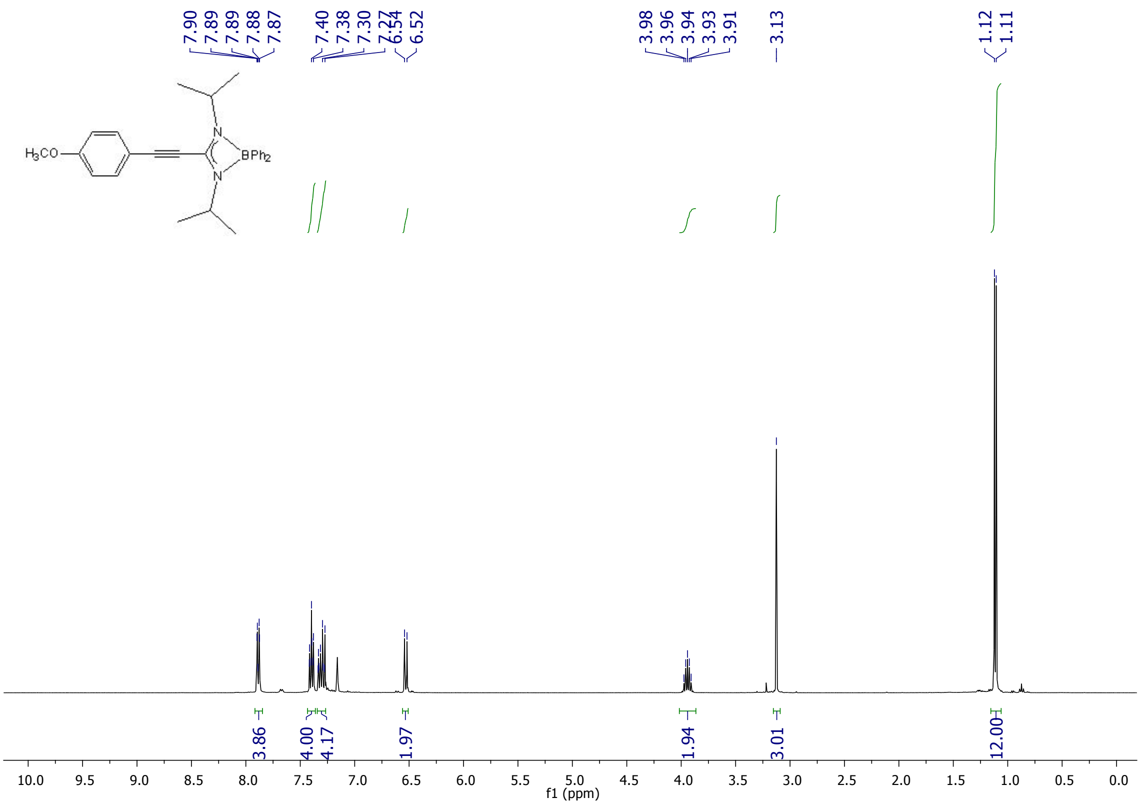

Figure S28. ^{1}H-NMR full chart for **4b** in C_6D_6.

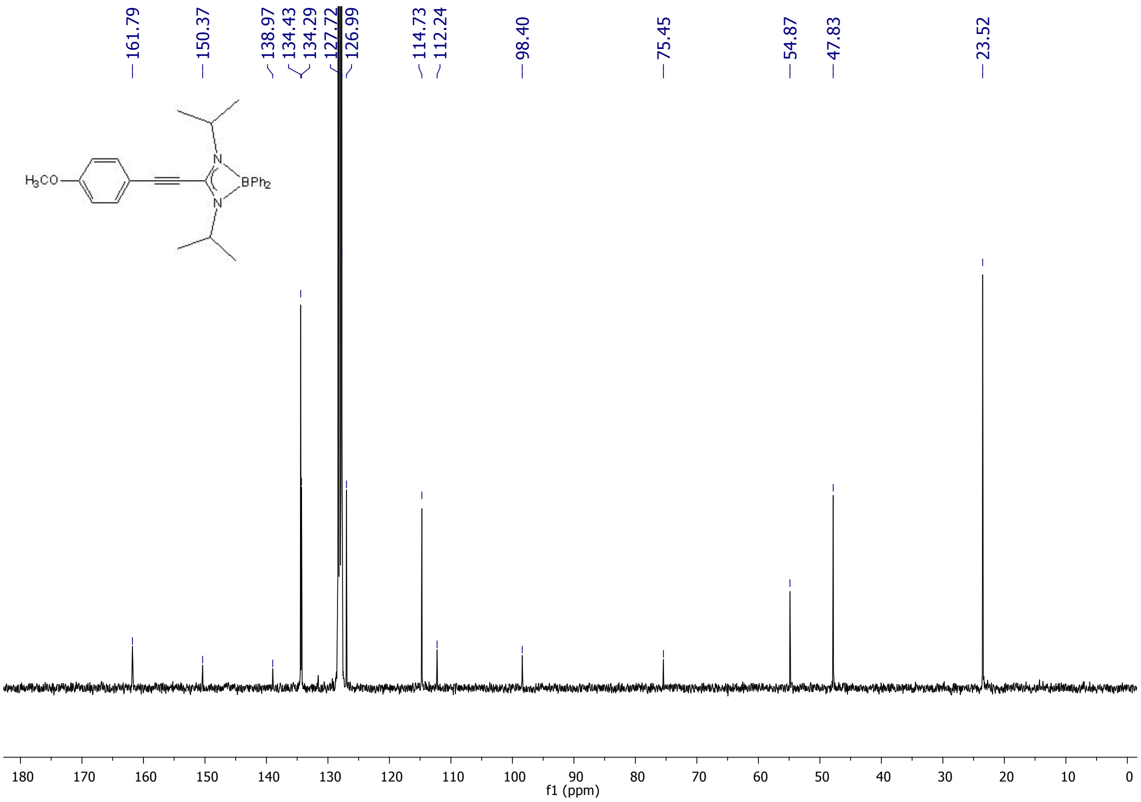

Figure S29. ^{13}C{^{1}H}-NMR full chart for **4b** in C_6D_6.

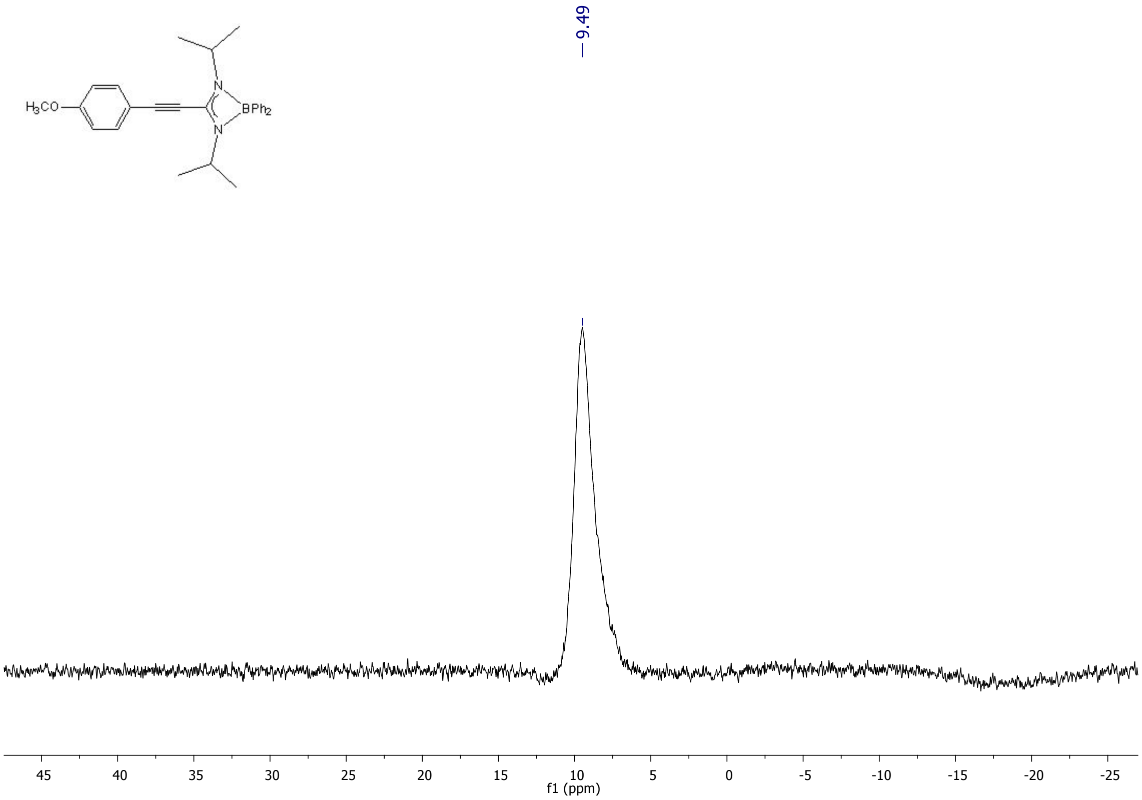

Figure S30. $^{11}B\{^{1}H\}$-NMR full chart for **4b** in C_6D_6.

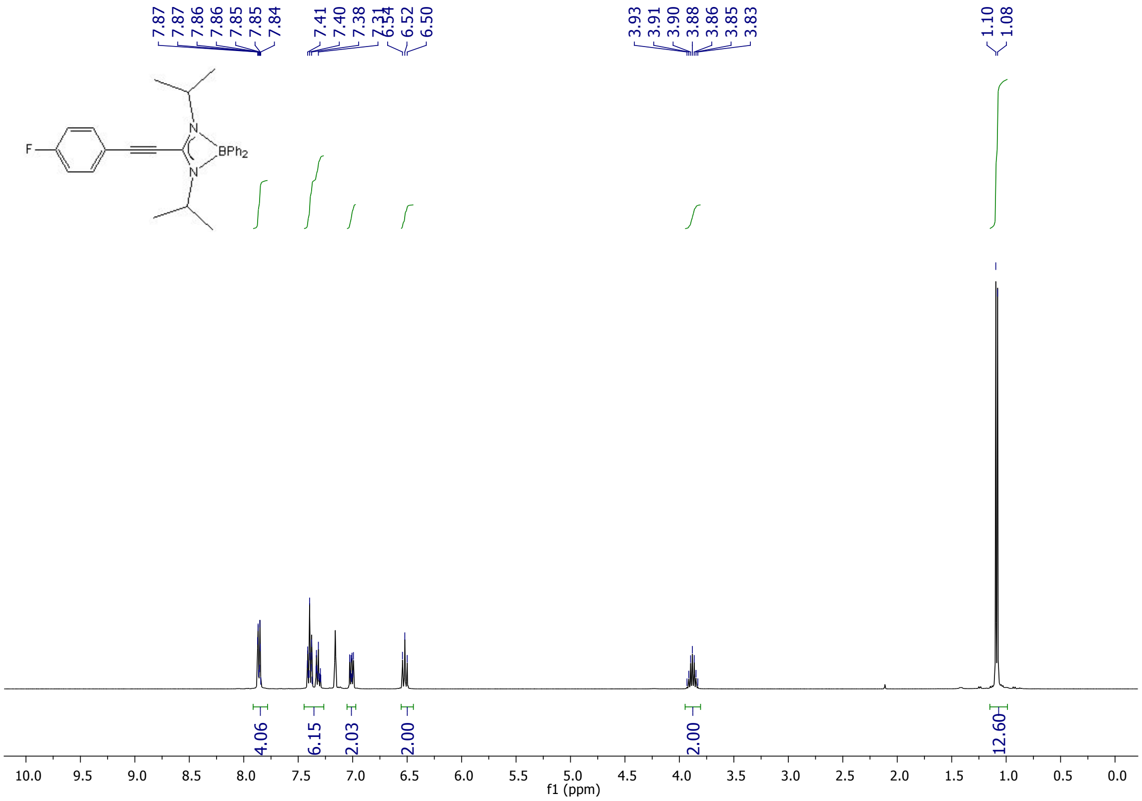

Figure S31. ^{1}H-NMR full chart for **4c** in C_6D_6.

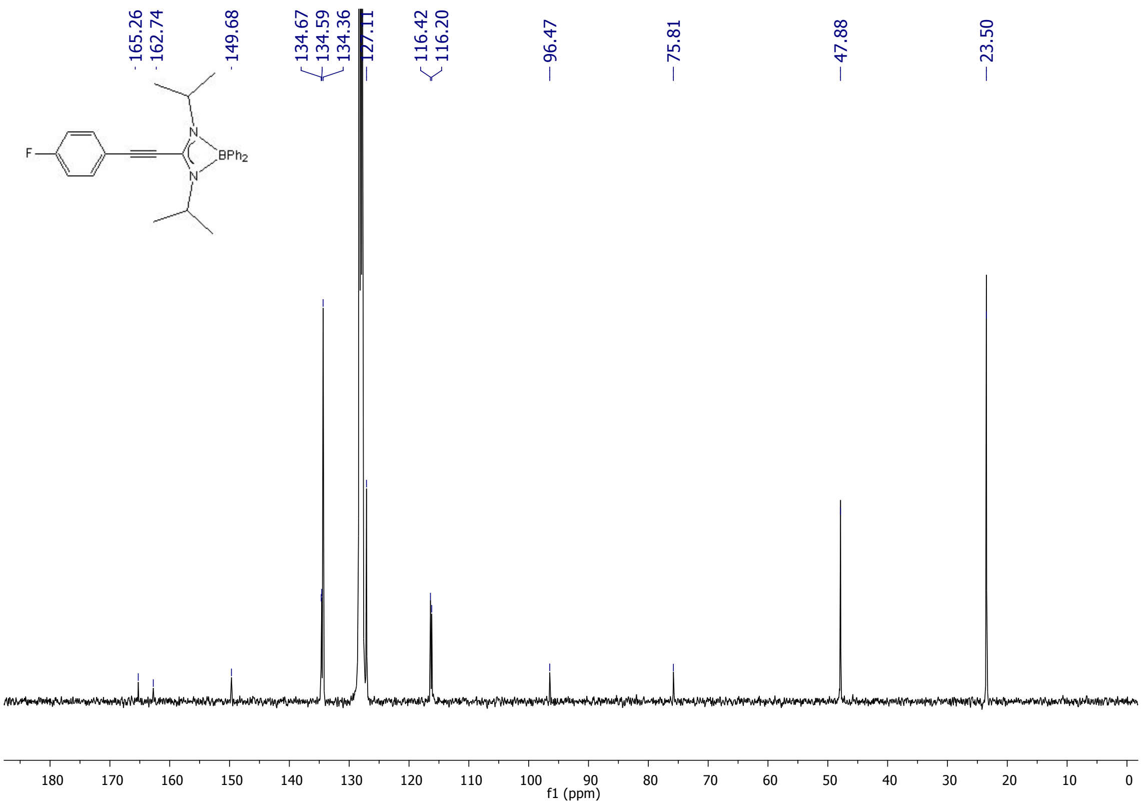

Figure S32. $^{13}C\{^1H\}$-NMR full chart for **4c** in C_6D_6.

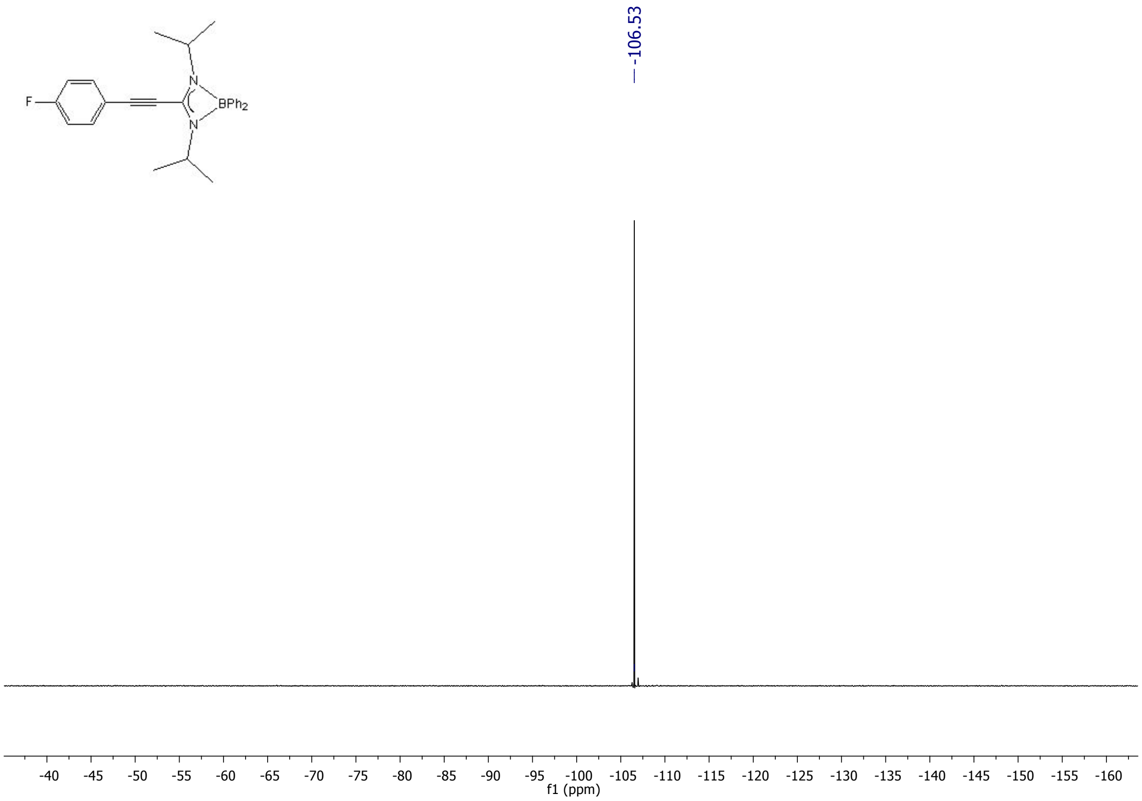

Figure S33. $^{19}F\{^1H\}$-NMR full chart for **4c** in C_6D_6.

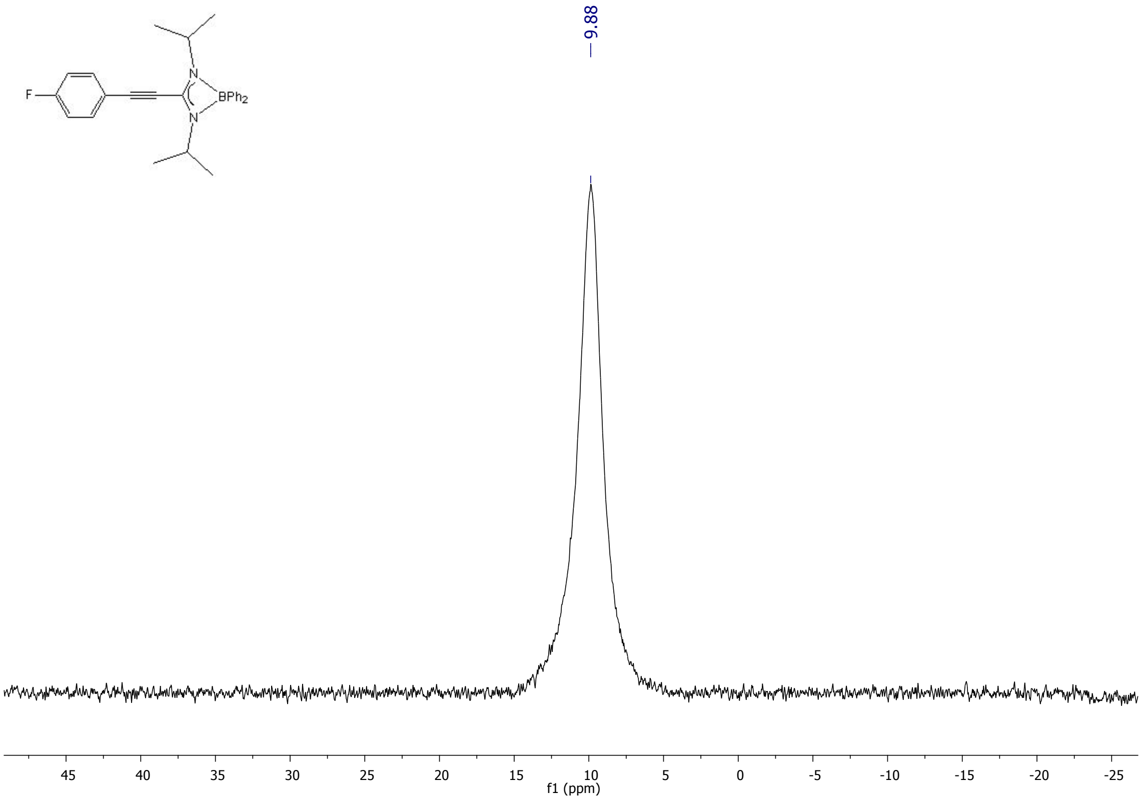

Figure S34. $^{11}B\{^{1}H\}$-NMR full chart for **4c** in C_6D_6.

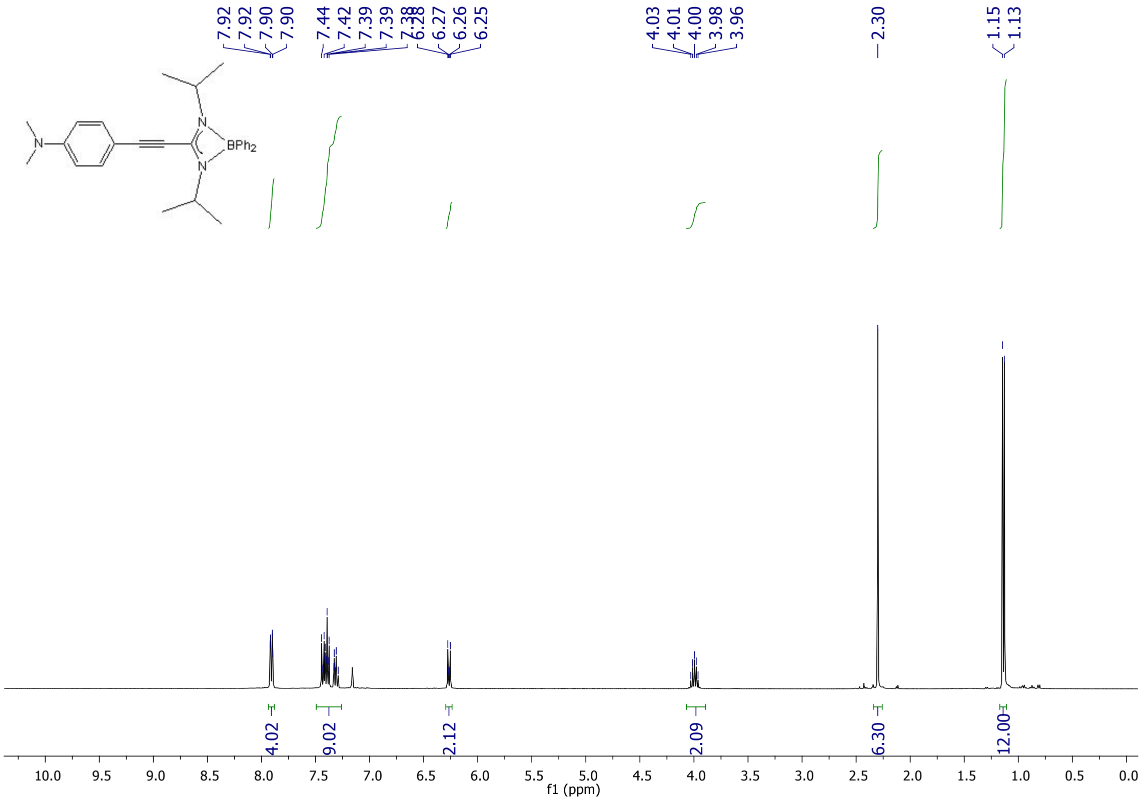

Figure S35. ^{1}H-NMR full chart for **4d** in C_6D_6.

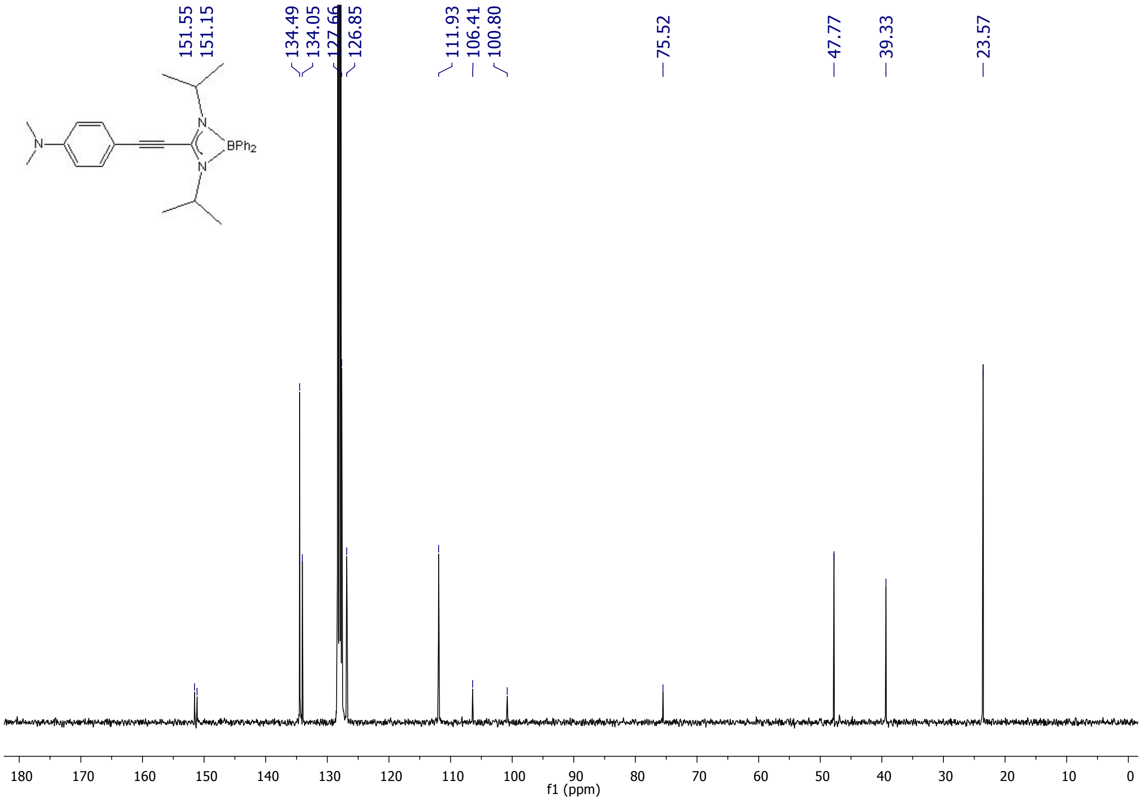

Figure S36. $^{13}C\{^1H\}$-NMR full chart for **4d** in C_6D_6.

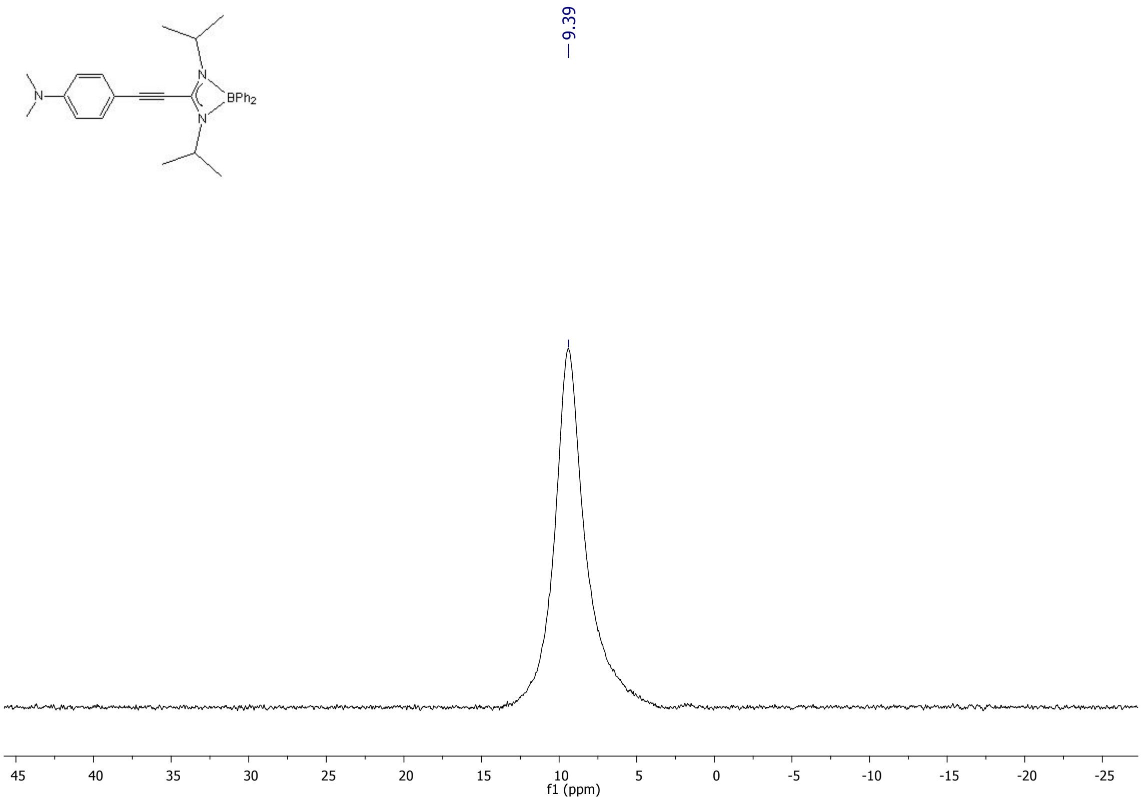

Figure S37. $^{11}B\{^1H\}$-NMR full chart for **4d** in C_6D_6.

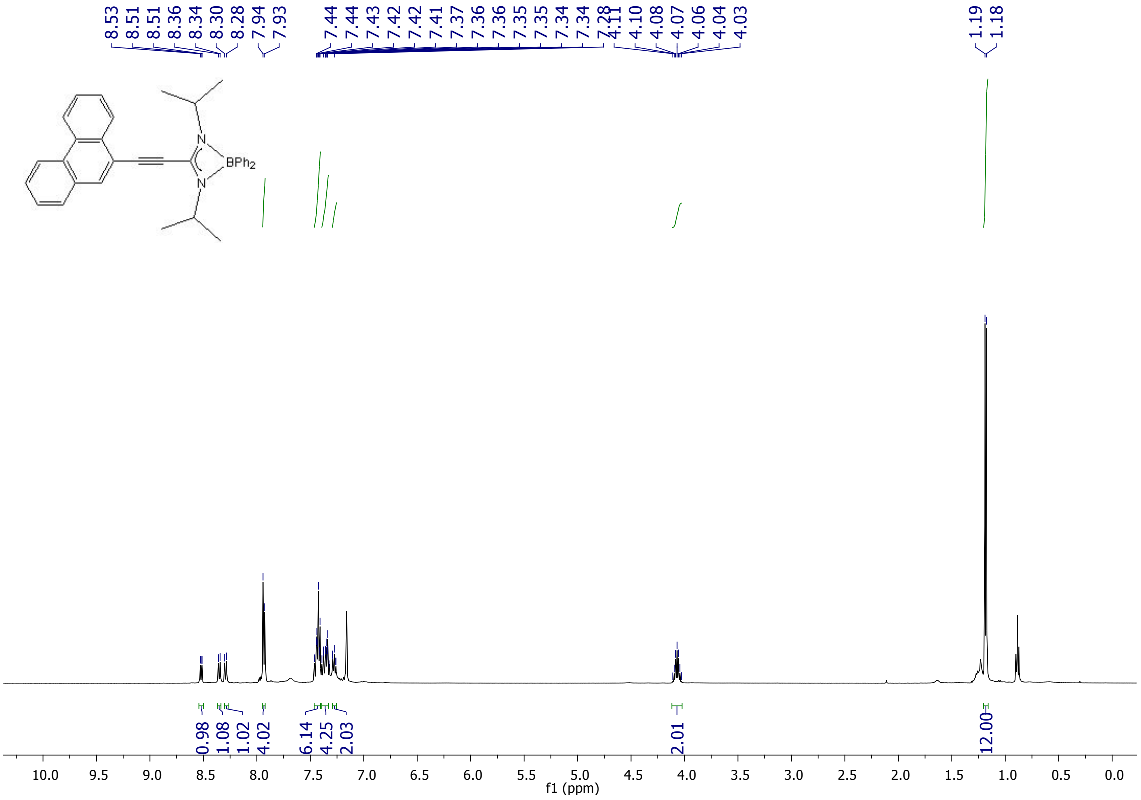

Figure S38. ^{1}H-NMR full chart for **4e** in C_6D_6.

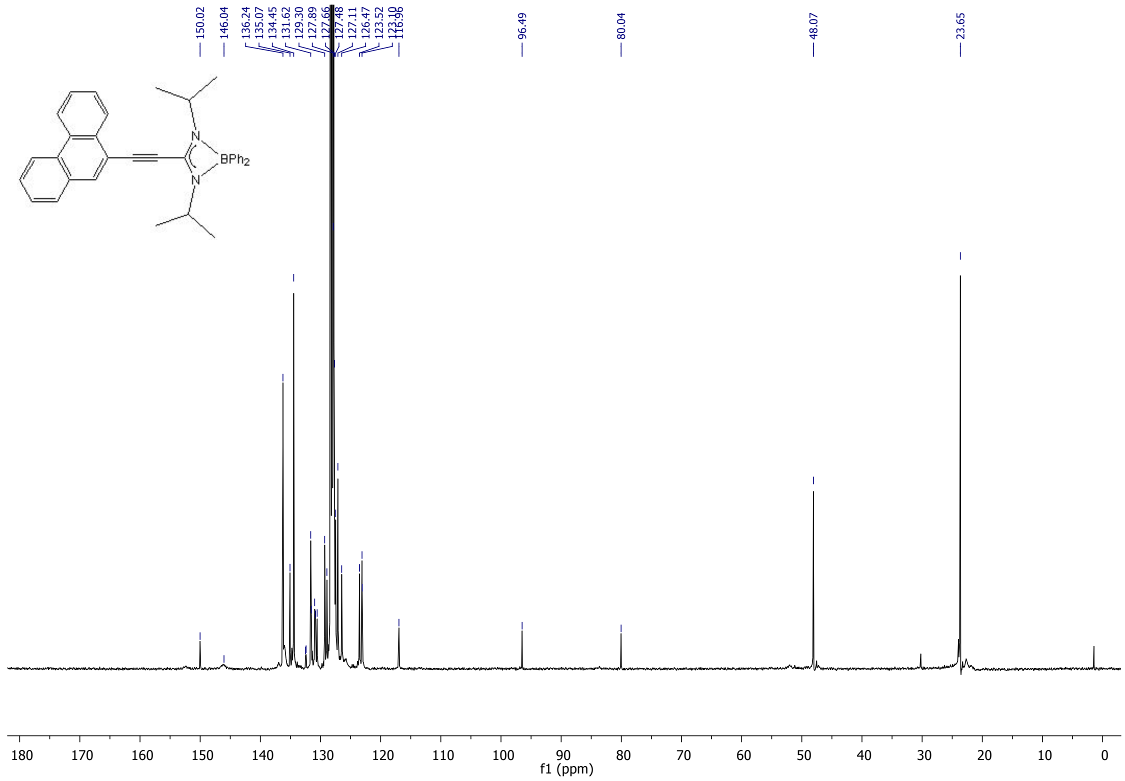

Figure S39. $^{13}C\{^{1}H\}$-NMR full chart for **4e** in C_6D_6.

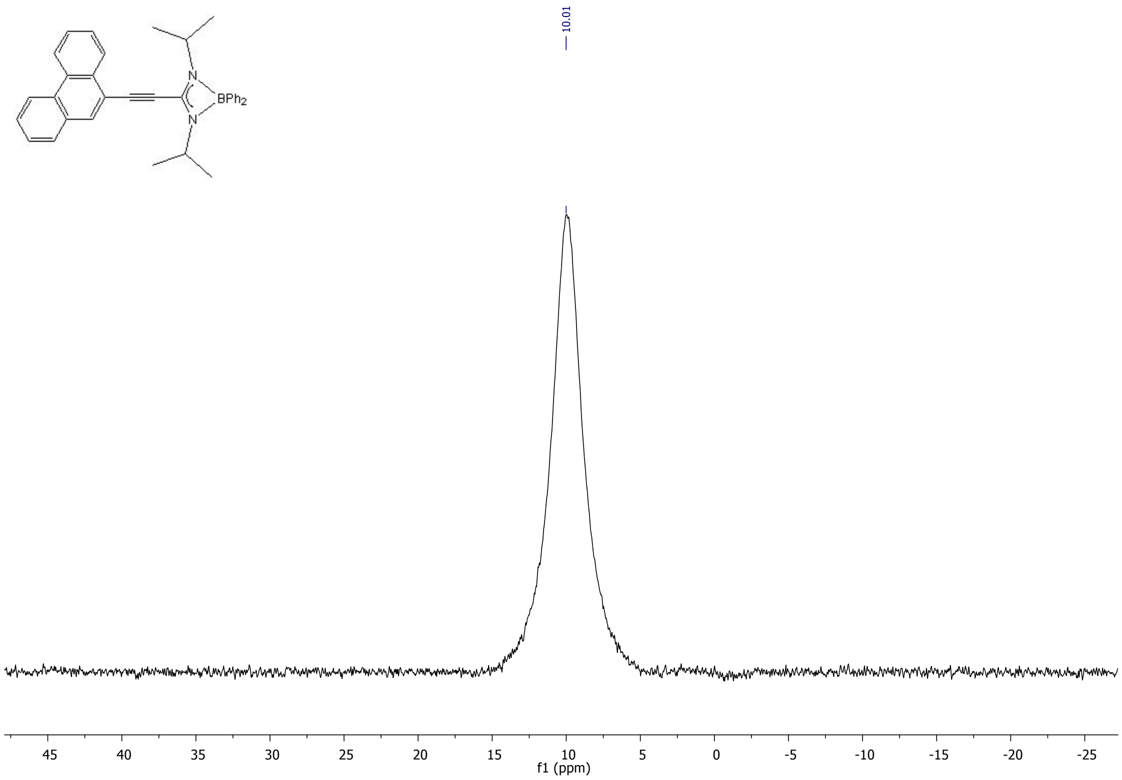

Figure S40. $^{11}B\{^{1}H\}$-NMR full chart for **4e** in C_6D_6.

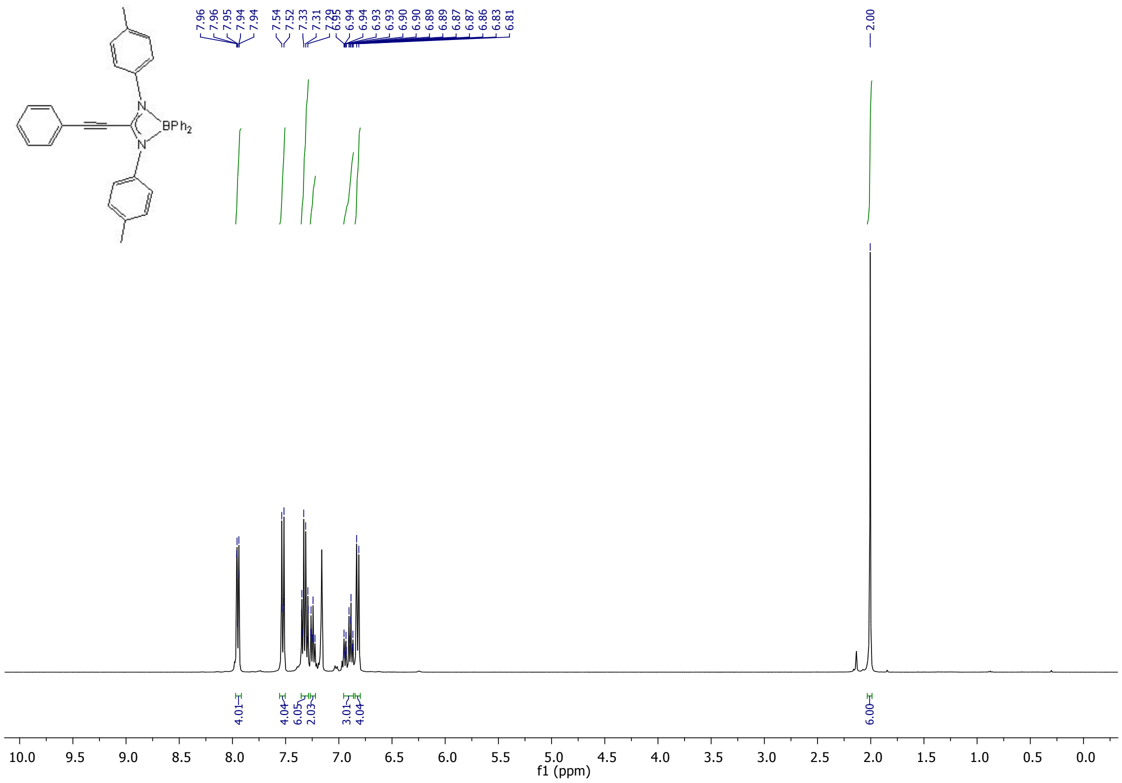

Figure S41. ^{1}H-NMR full chart for **6a** in C_6D_6.

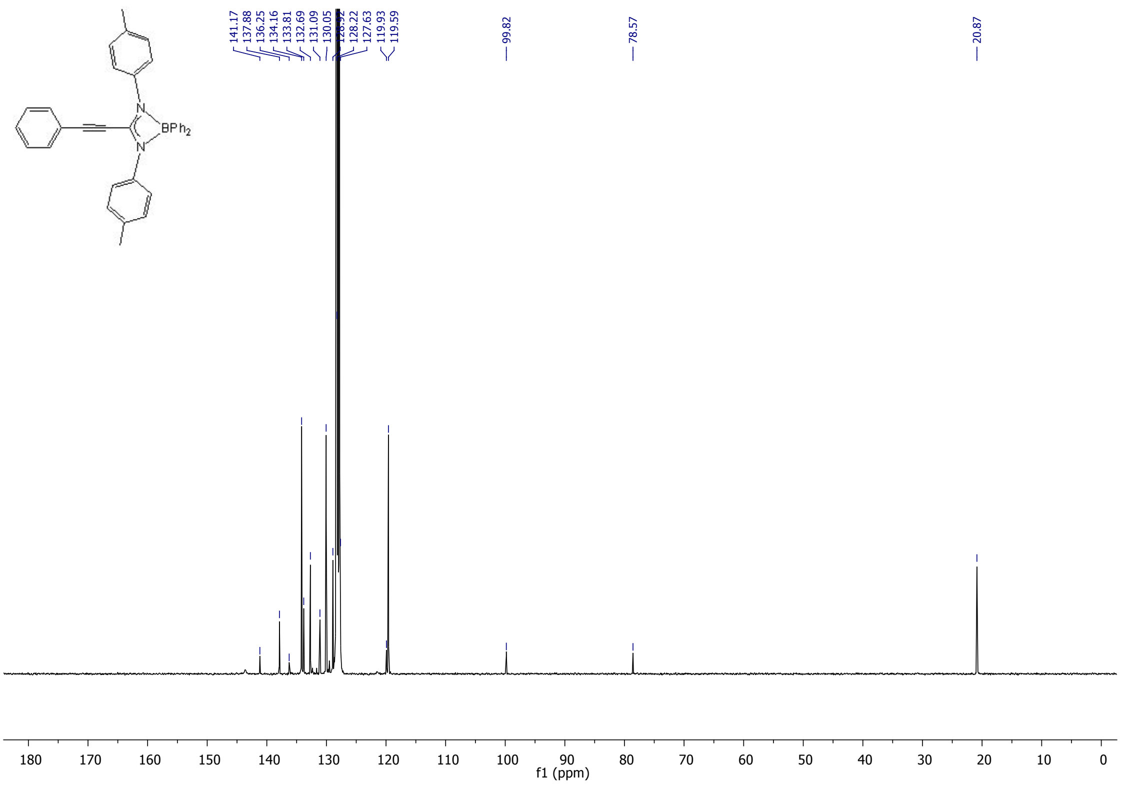

Figure S42. $^{13}C\{^{1}H\}$-NMR full chart for **6a** in C_6D_6.

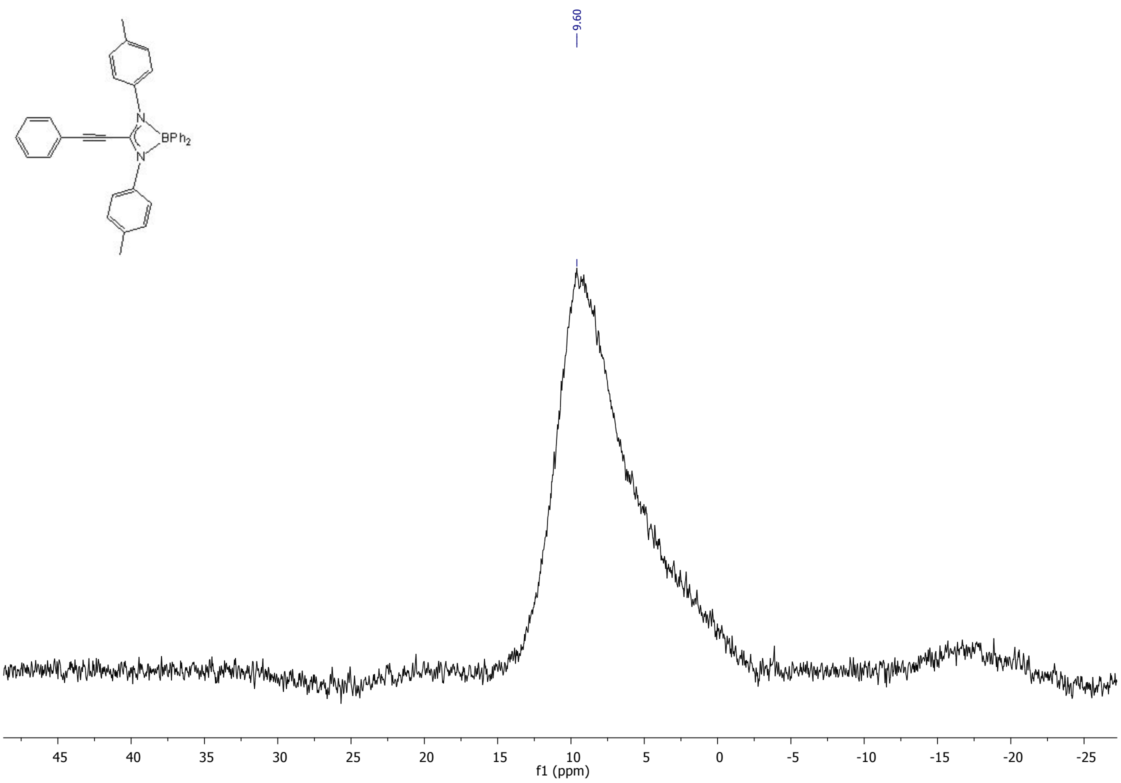

Figure S43. $^{11}B\{^{1}H\}$-NMR full chart for **6a** in C_6D_6.

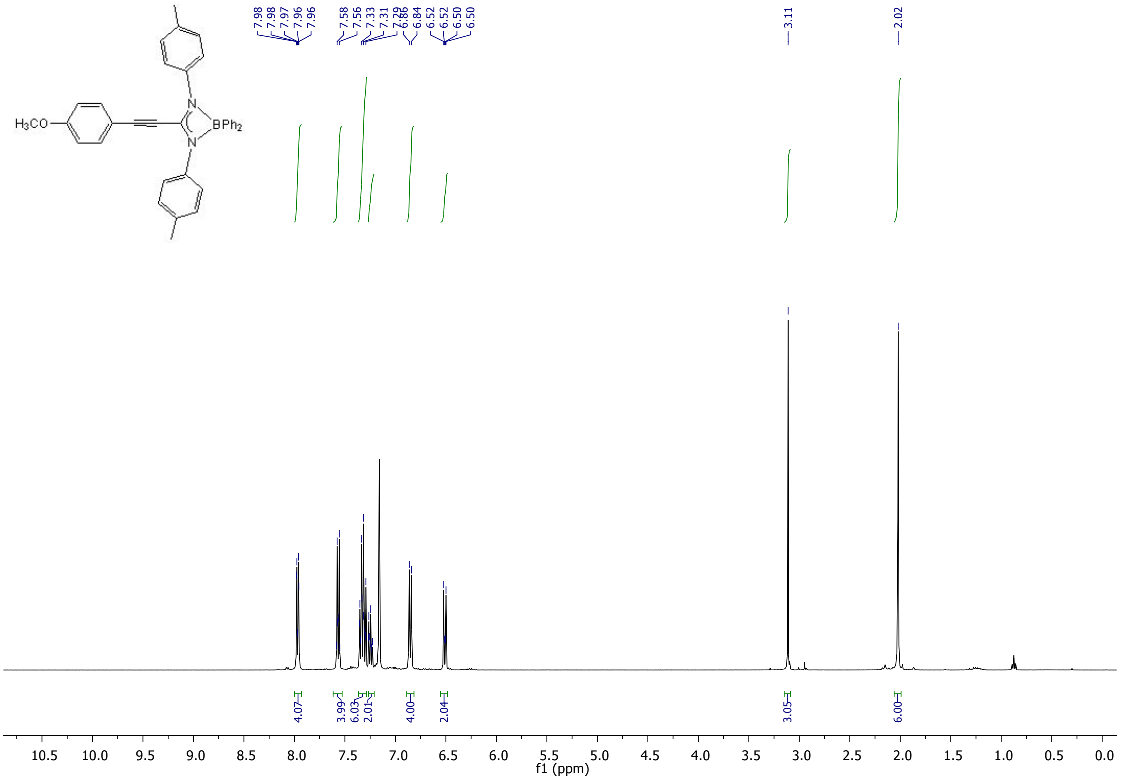

Figure S44. ^{1}H-NMR full chart for **6b** in C_6D_6.

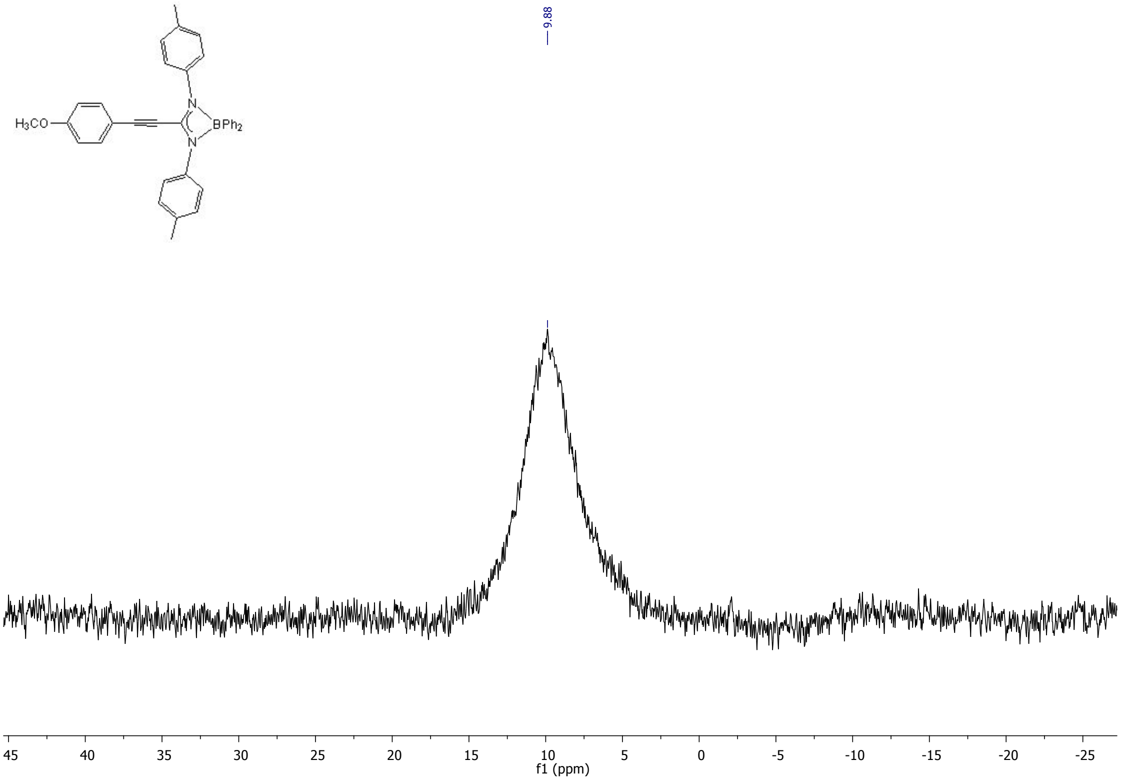

Figure S45. $^{13}C\{^{1}H\}$-NMR full chart for **6b** in C_6D_6.

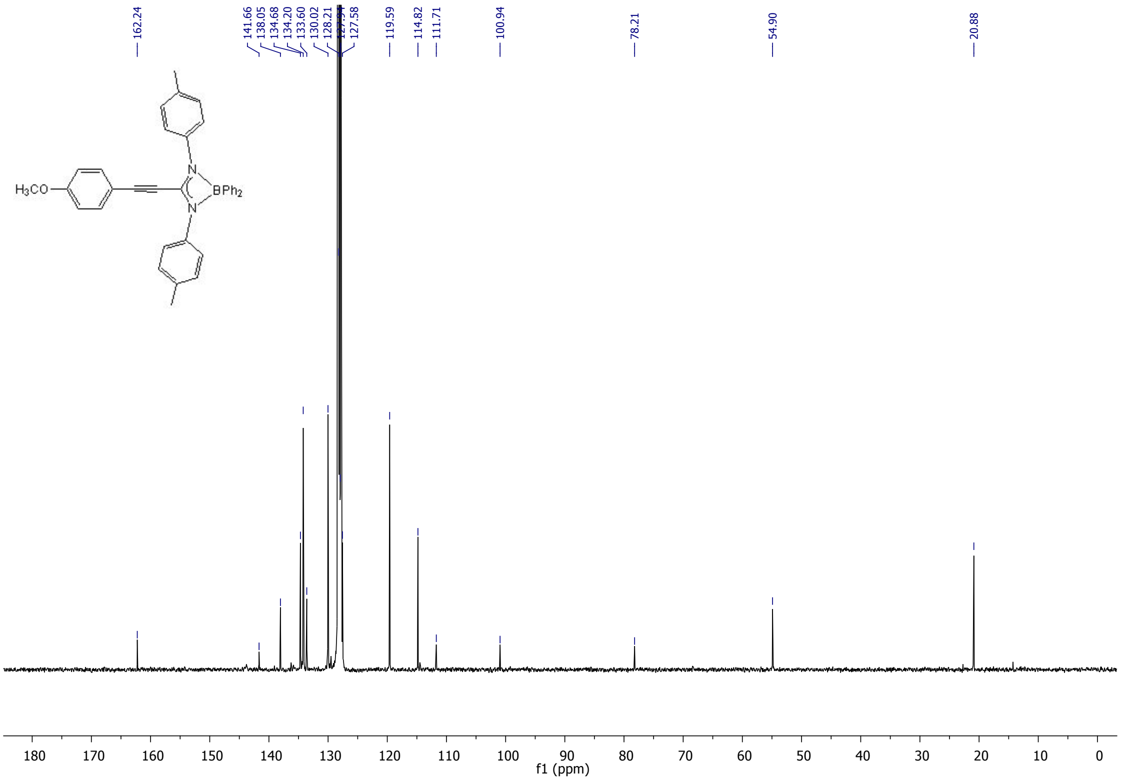

Figure S46. $^{11}B\{^{1}H\}$-NMR full chart for **6b** in C_6D_6.

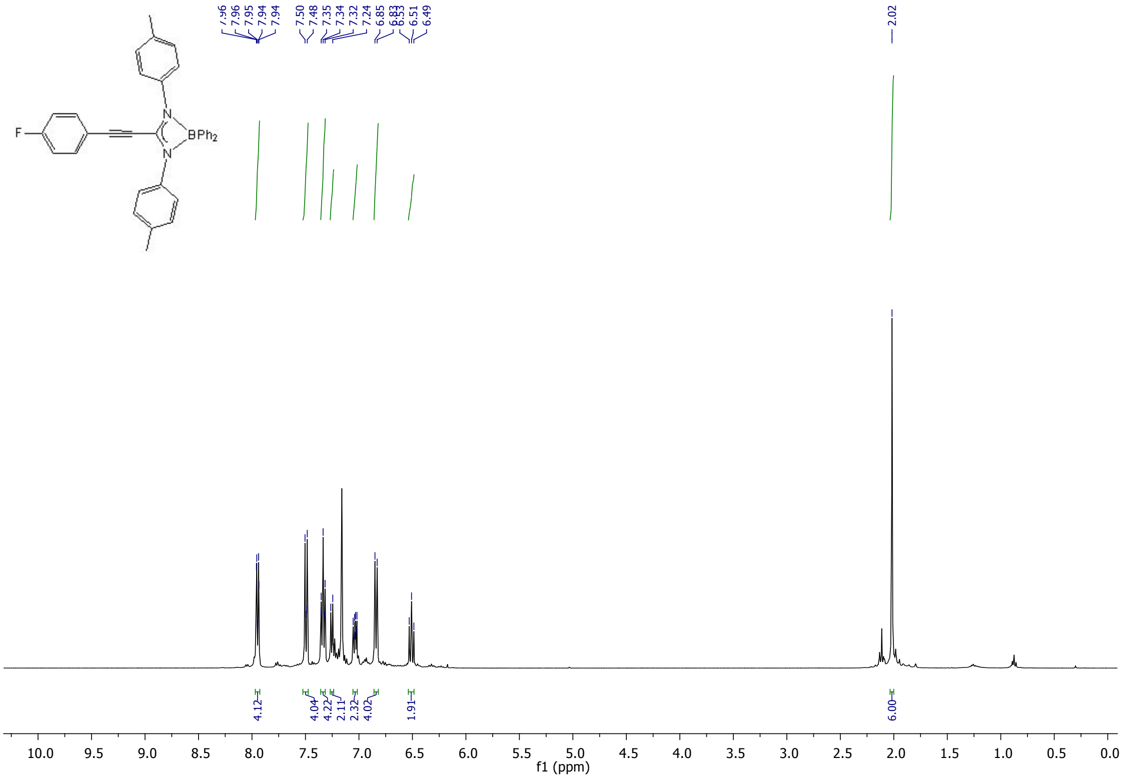

Figure S47. ^{1}H-NMR full chart for **6c** in C_6D_6.

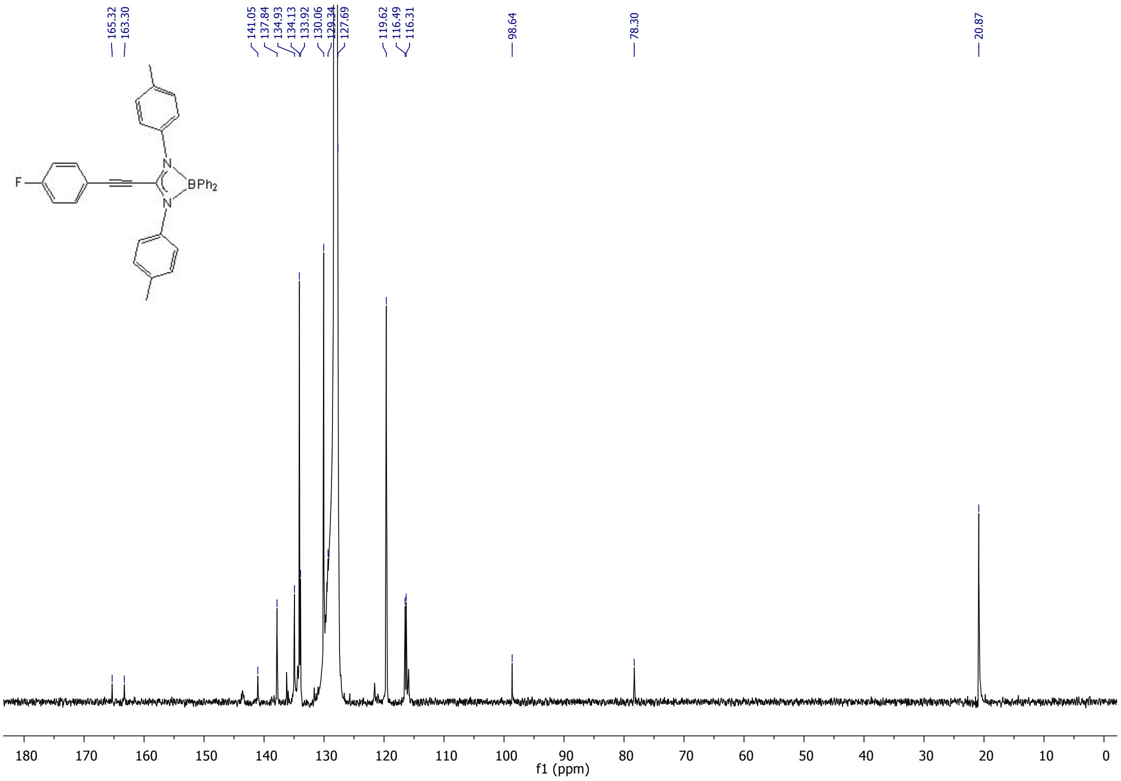

Figure S48. $^{13}C\{^{1}H\}$-NMR full chart for **6c** in C_6D_6.

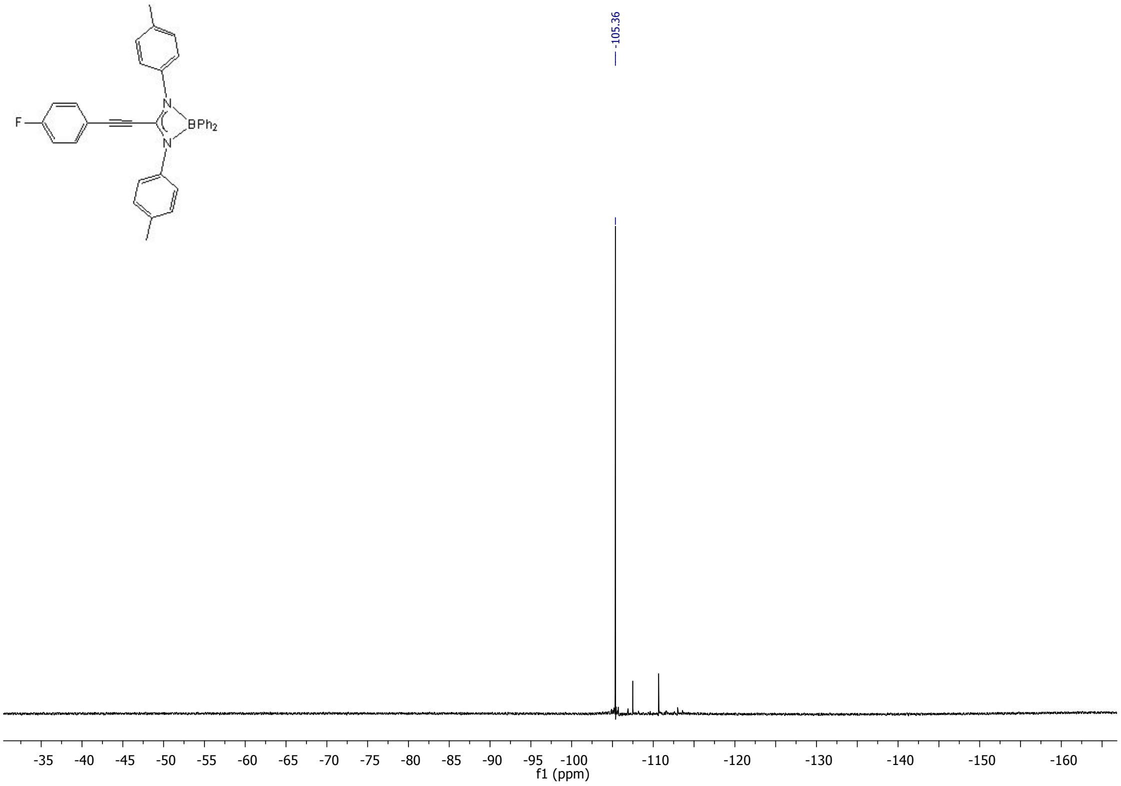

Figure S49. $^{19}F\{^{1}H\}$-NMR full chart for **6c** in C_6D_6.

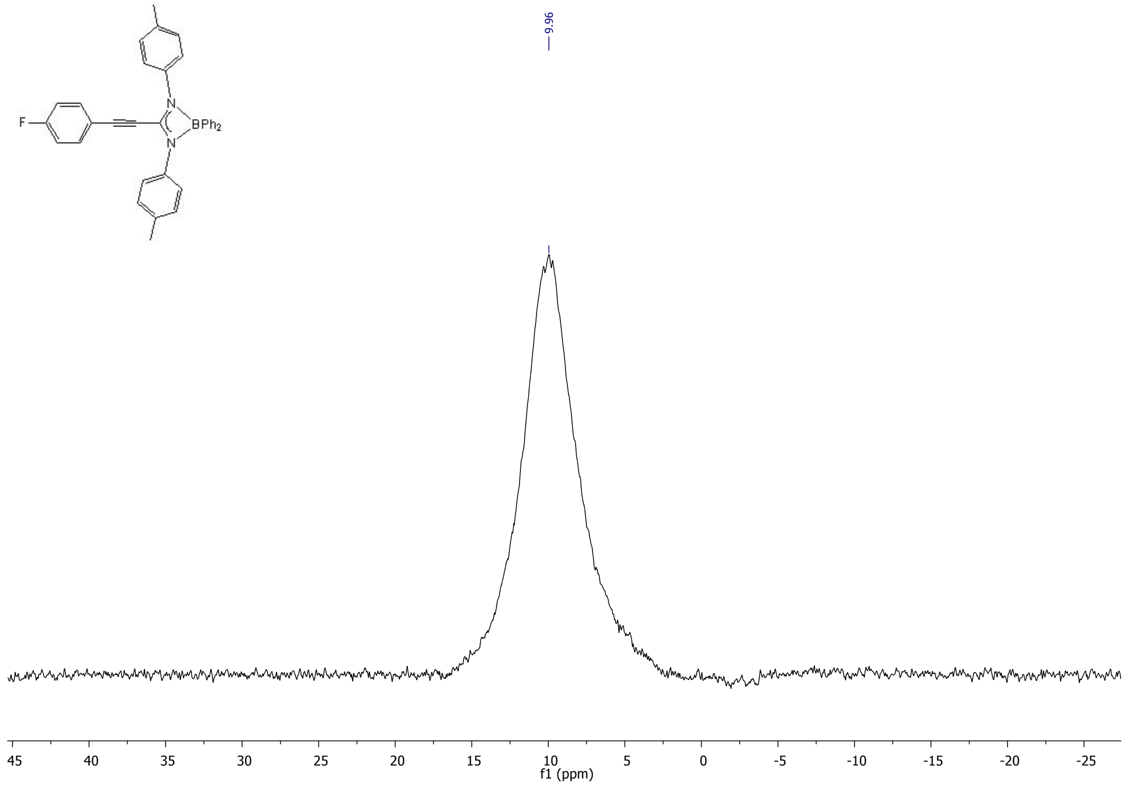

Figure S50. $^{11}B\{^{1}H\}$-NMR full chart for **6c** in C_6D_6.

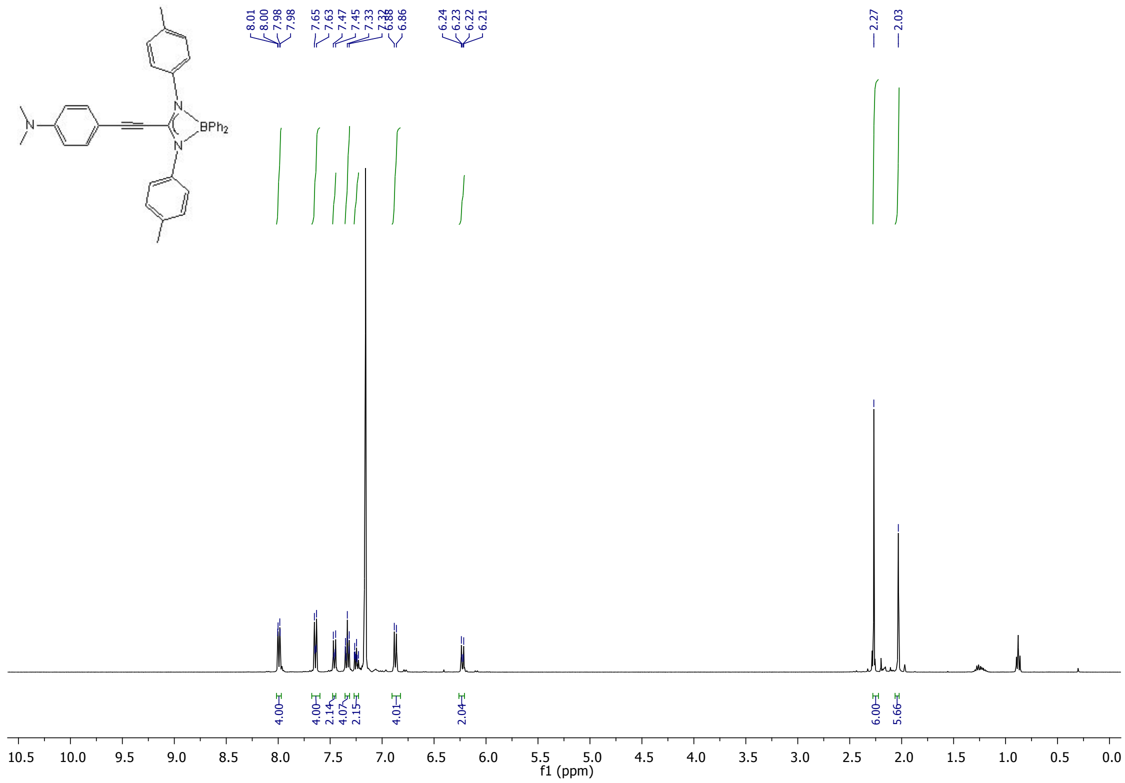

Figure S51. ^{1}H-NMR full chart for **6d** in C_6D_6.

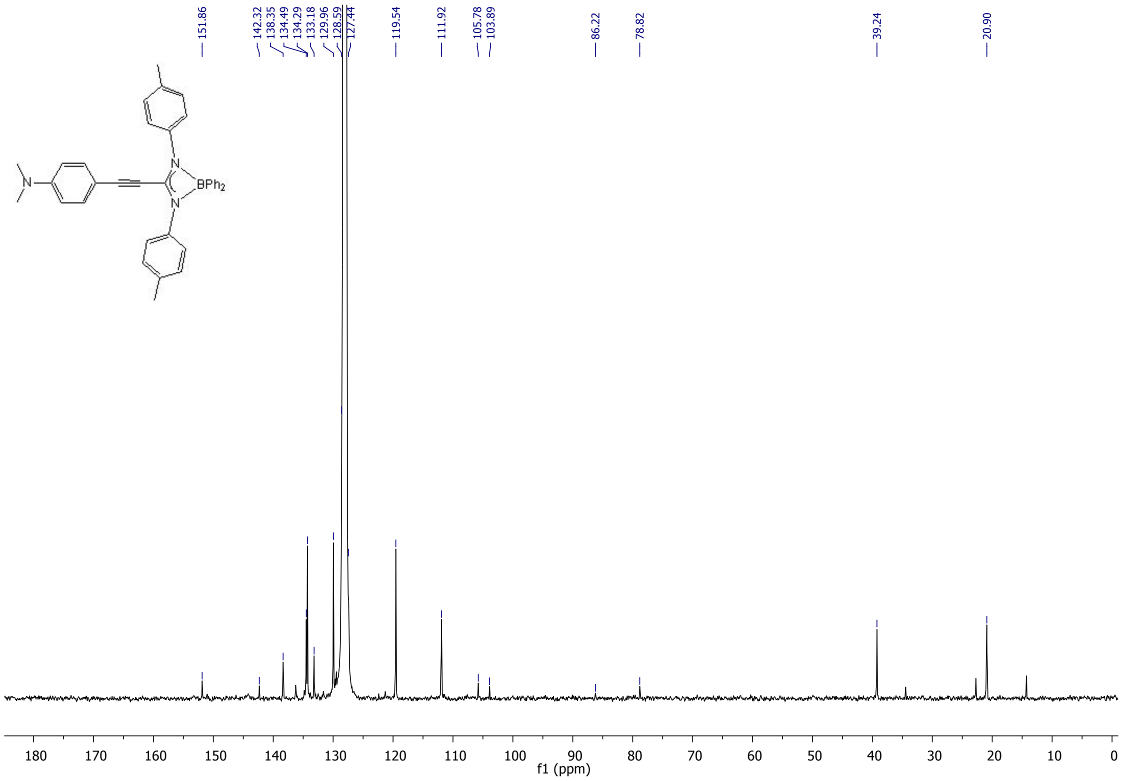

Figure S52. $^{13}C\{^{1}H\}$-NMR full chart for **6d** in C_6D_6.

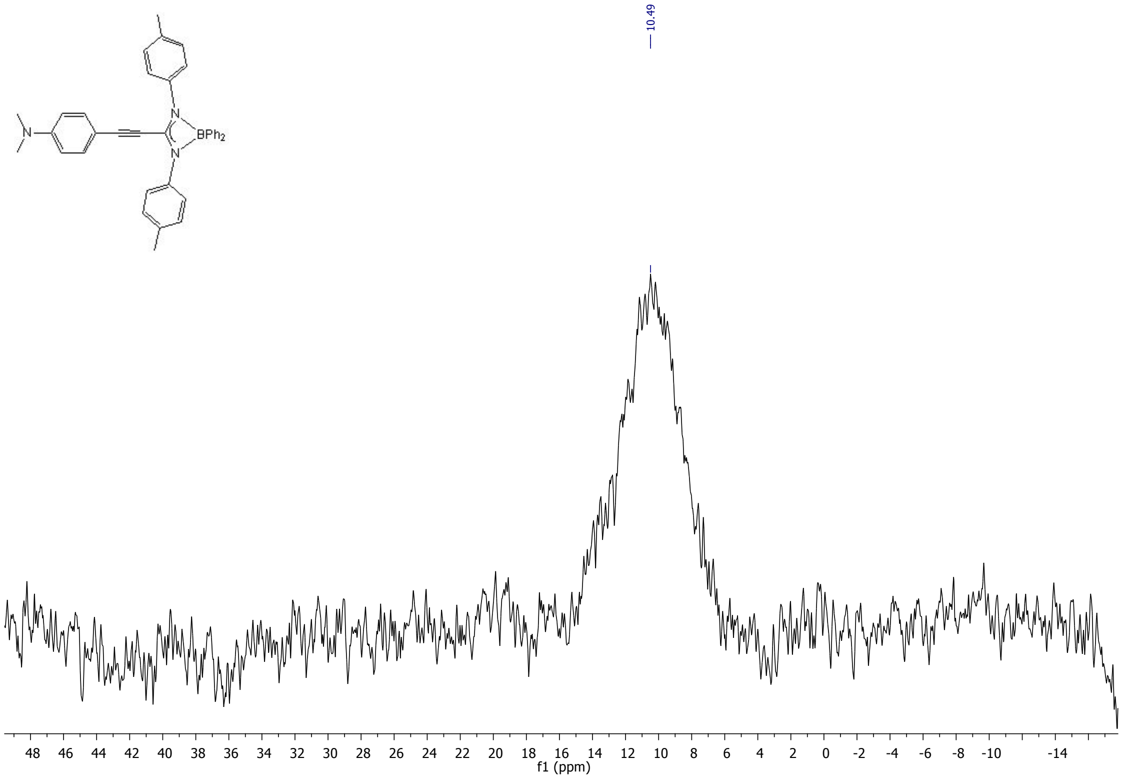

Figure S53. $^{11}B\{^{1}H\}$-NMR full chart for **6d** in C_6D_6.

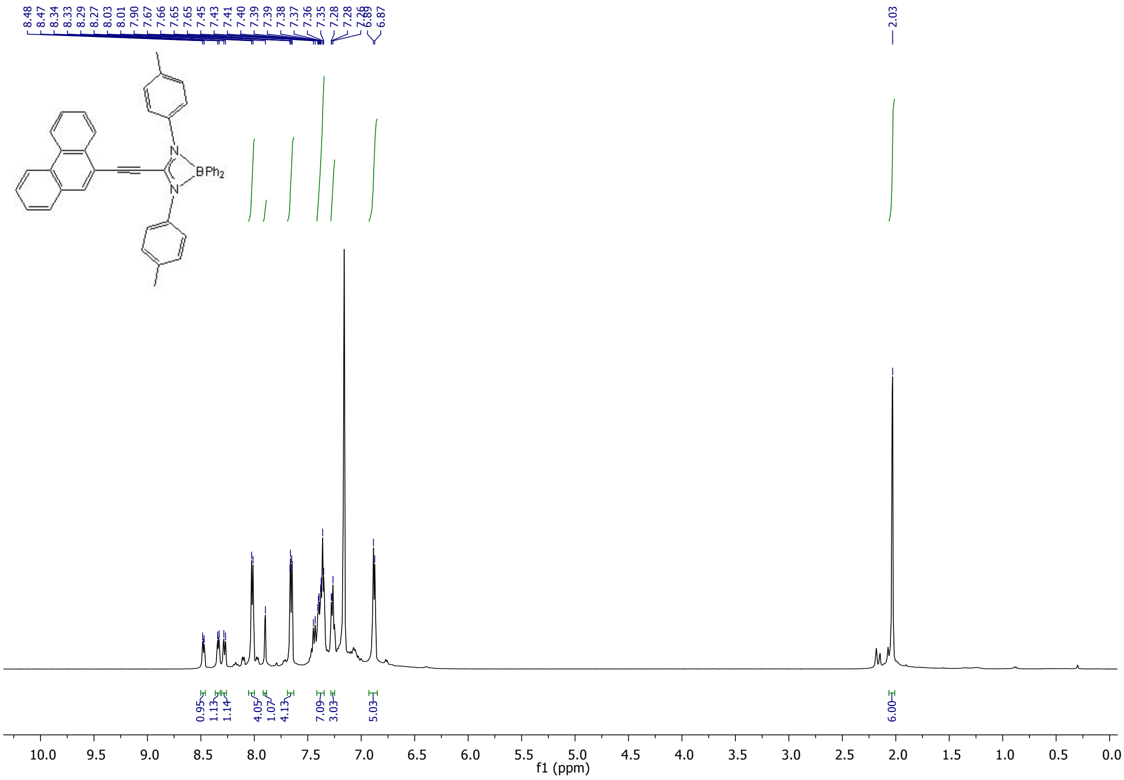

Figure S54. ^{1}H-NMR full chart for **6e** in C_6D_6.

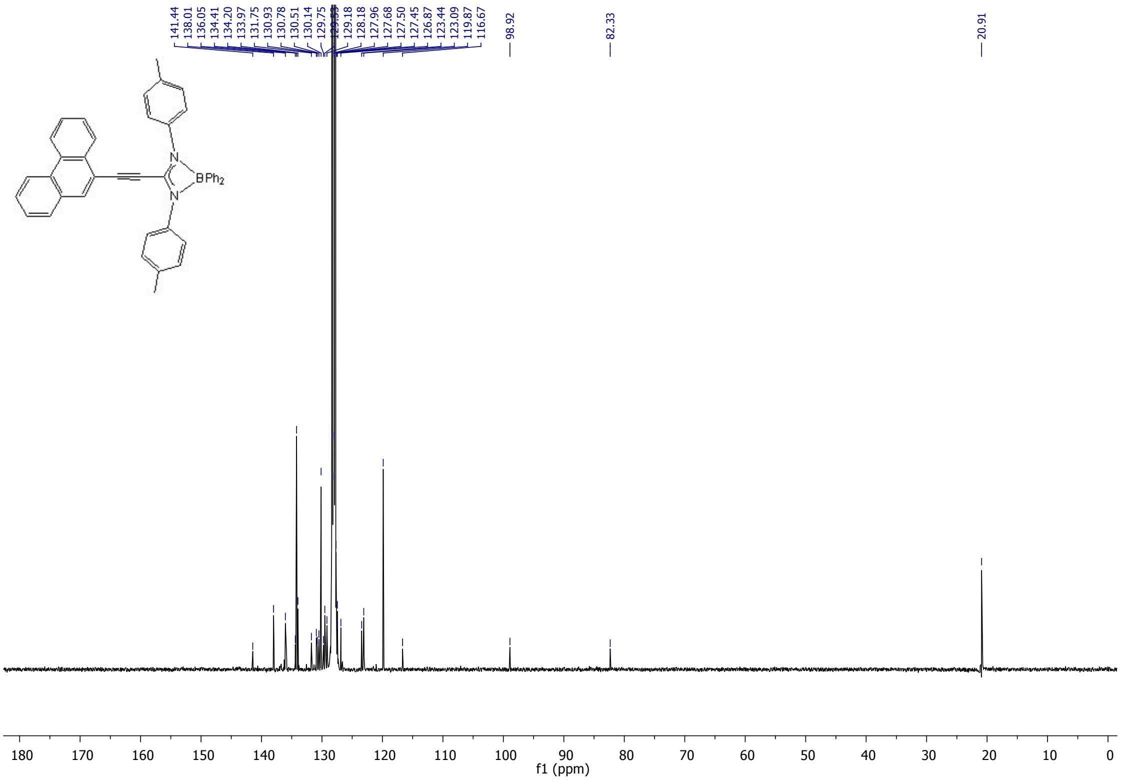

Figure S55. ^{13}C{^{1}H}-NMR full chart for **6e** in C_6D_6.

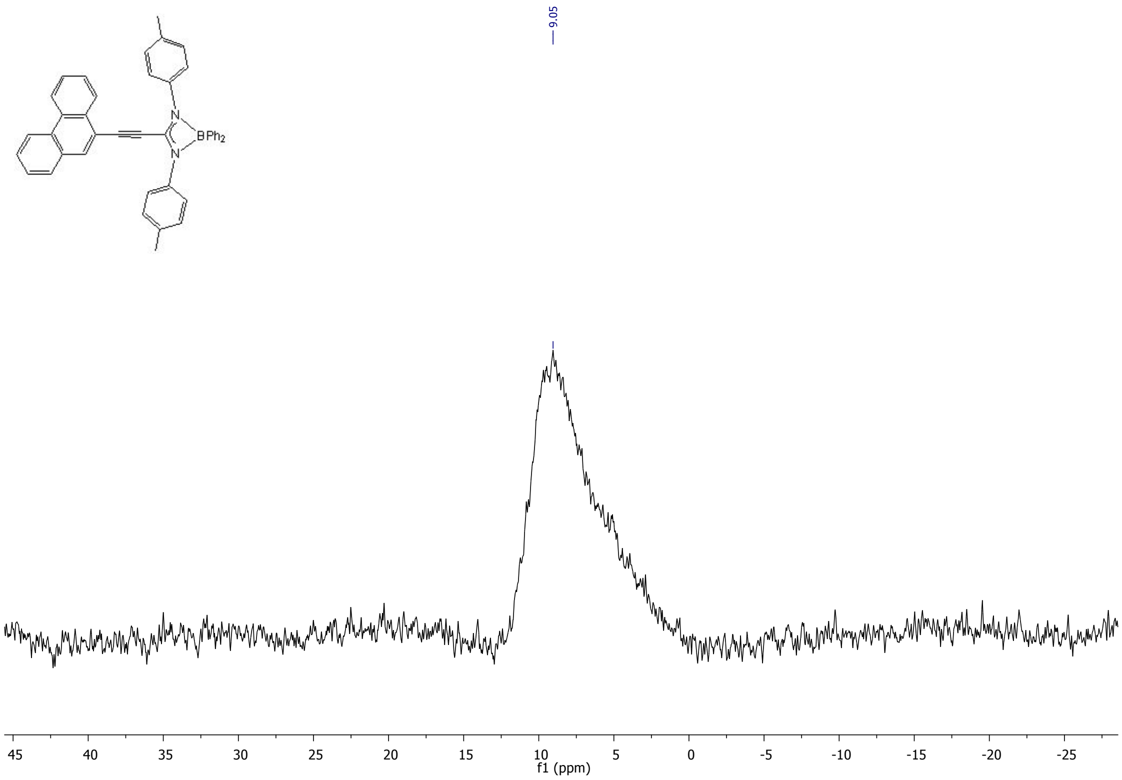

Figure S56. $^{11}B\{^{1}H\}$-NMR full chart for **6e** in C_6D_6.

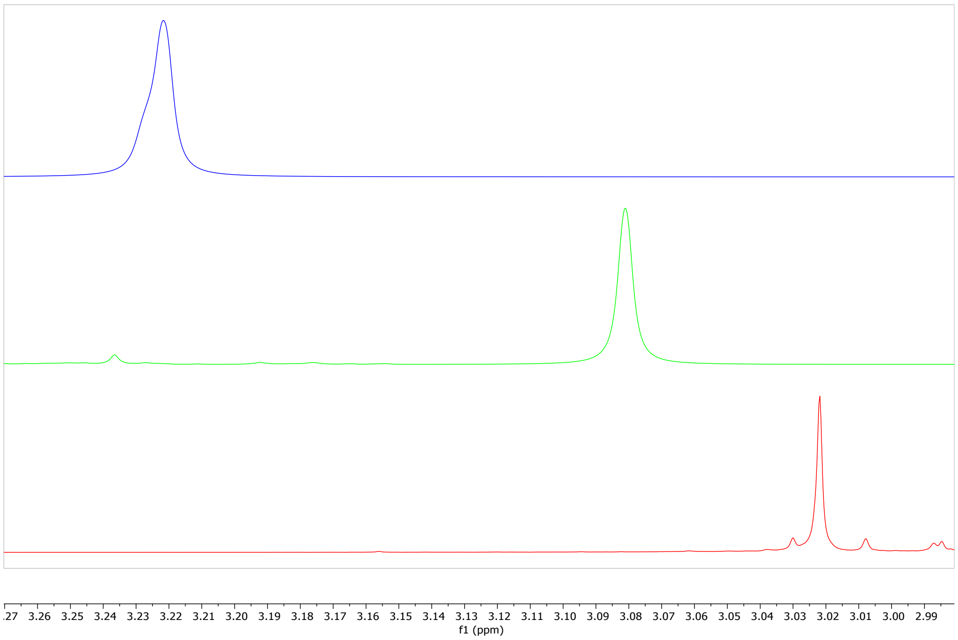

Figure S57. ^{1}H NMR spectra of **3d** after reaction with triflic acid (up), **4d** after reaction with triflic acid (middle) and **4d** (bottom), in CD_3CN, in the region of the NMe_2 group.

4.- X-ray structural analyses

Crystals were mounted on a glass fibre and cooled to the 100 K for compound **4a**, 150K for compounds **4e** and **6a**, and room temperature for compounds **4b**, **4c**, **6e** and **6d**. Data were collected on a Bruker APEX II CCD-based diffractometer equipped with a graphite monochromated MoKα radiation source (λ=0.71073 Å). Intensities were integrated in SAINT [3] and absorption corrections based on equivalent reflections were applied using SADABS. [4] Structures were solved using ShelXT, [5] all of the structures were refined by full matrix least squares against F^2 in ShelXL [6] using Olex2. [7] All of the non-hydrogen atoms were refined anisotropically, while all of the hydrogen atoms were located geometrically and refined using a riding model. Compounds **4a**, **4b**, **4c**, **6e** and **6d** show some disordered fragments and the occupancies of the disordered group were refined with their sum set to equal 1 and subsequently fixed at the refined values. Restraints were applied to maintain sensible thermal and geometric parameters. The structure of compound **6d** was also refined as a two-component twin (-1 0 0 0 -1 0 0 0 1), with twin fraction 0.496(2). Moreover, in compound **4b** due to the quality of the crystals some data was rejected as poor during integration and scaling, being the origin of the alerts B during checkcif test. The X-ray crystallographic coordinates for structures reported in this study have been deposited at the Cambridge Crystallographic Data Centre (CCDC) under deposition numbers 2307608– 2307613 for **4a**, **4b**, **4c**, **4e**, **6a**, **6d** and **6e** respectively. These data can be obtained free of charge via http://www.ccdc.cam.ac.uk/data_request/cif

Table S1. Crystal data and structure refinement for **4a**, **4b**, **4c** and **4e**.

Identification code	**4a**	**4b**	**4c**	**4e**
Empirical formula	$C_{27}H_{29}BN_2$	$C_{28}H_{31}BN_2O$	$C_{27}H_{28}BFN_2$	$C_{75}H_{78}B_2N_4$
Formula weight	392.33	422.36	410.32	1057.03
Temperature/K	100.15	296.15	296.15	150.16
Crystal system	monoclinic	monoclinic	monoclinic	triclinic
Space group	$P2_1/n$	$P2_1/n$	$P2_1/n$	P-1
a/Å	9.5576(3)	9.823(16)	9.716(15)	9.2936(4)
b/Å	18.0239(8)	18.37(3)	18.14(3)	11.0040(6)
c/Å	13.8891(4)	14.50(3)	14.07(2)	15.8337(9)
α/°	90	90	90	98.173(2)
β/°	101.516(3)	101.37(3)	101.32(2)	106.630(2)
γ/°	90	90	90	95.270(2)

Volume/Å^3	2344.44(15)	2566(8)	2432(6)	1520.70(14)
Z	4	4	4	1
ρ_{calc}g/cm^3	1.112	1.093	1.121	1.154
μ/mm^{-1}	0.064	0.065	0.070	0.066
F(000)	840.0	904.0	872.0	566.0
Crystal size/mm^3	0.313 × 0.293 × 0.284	0.614 × 0.360 × 0.201	0.421× 0.205 × 0.234	0.271 × 0.271 × 0.086
Radiation	MoKα (λ = 0.71073)	MoKα (λ = 0.71073)	MoKα (λ = 0.71073)	MoKα (λ = 0.71073)
2Θ range for data collection/°	5.422 to 50.788	4.776 to 54.034	5.204 to 55.476	3.776 to 52.934
Index ranges	-10 ≤ h ≤ 11, -21 ≤ k ≤ 21, -16 ≤ l ≤ 15	-12 ≤ h ≤ 9, -22 ≤ k ≤ 21, -17 ≤ l ≤ 18	-12 ≤ h ≤ 9, -23 ≤ k ≤ 23, -18 ≤ l ≤ 18	-11 ≤ h ≤ 11, -13 ≤ k ≤ 13, -19 ≤ l ≤ 19
Reflections collected	21988	12776	18874	92440
Independent reflections	4298 [R_{int} = 0.0296, R_{sigma} = 0.0251]	5228 [R_{int} = 0.0765, R_{sigma} = 0.0957]	5685 [R_{int} = 0.0547, R_{sigma} = 0.0679]	6244 [R_{int} = 0.0645, R_{sigma} = 0.0305]
Data/restraints/parameters	4298/14/296	5228/1036/516	5685/50/305	6244/14/394
Goodness-of-fit on F^2	1.032	1.247	1.049	1.060
Final R indexes [I>=2σ (I)]	R_1 = 0.0408, wR_2 = 0.1021	R_1 = 0.1722, wR_2 = 0.3835	R_1 = 0.0807, wR_2 = 0.1684	R_1 = 0.0547, wR_2 = 0.1464
Final R indexes [all data]	R_1 = 0.0545, wR_2 = 0.1110	R_1 = 0.2207, wR_2 = 0.4223	R_1 = 0.1529, wR_2 = 0.2001	R_1 = 0.0852, wR_2 = 0.1691
Largest diff. peak/hole / e Å^{-3}	0.24/-0.29	0.62/-0.56	0.18/-0.22	0.37/-0.41

Table S2. Crystal data and structure refinement for **6a**, **6d** and **6e**.

Identification code	**6a**	**6e**	**6d**
Empirical formula	$C_{35}H_{29}BN_2$	$C_{37}H_{34}BN_3$	$C_{44}H_{35}BCl_2N_2$
Formula weight	488.41	531.48	673.45
Temperature/K	150.01	296.15	293
Crystal system	triclinic	triclinic	monoclinic
Space group	P-1	P-1	$P2_1/c$
a/Å	8.4512(5)	11.090(4)	9.689(4)
b/Å	11.5034(5)	11.227(4)	34.583(14)
c/Å	14.4397(8)	13.757(4)	10.707(5)

α/°	83.230(2)	104.370(4)	90
β/°	86.245(3)	92.473(4)	90.005(4)
γ/°	73.563(2)	111.352(5)	90
Volume/Å^3	1336.27(12)	1528.3(8)	3588(3)
Z	2	2	4
ρ_{calc}g/cm^3	1.214	1.155	1.247
μ/mm^{-1}	0.070	0.067	0.215
F(000)	516.0	564.0	1408.0
Crystal size/mm^3	0.201 × 0.084 × 0.038	0.301 × 0.191 × 0.189	0.112 × 0.101 × 0.043
Radiation	MoKα (λ = 0.71073)	MoKα (λ = 0.71073)	MoKα (λ = 0.71073)
2Θ range for data collection/°	7.398 to 50.696	5.092 to 53.852	3.982 to 49.996
Index ranges	$-10 \leq h \leq 10, -13 \leq k \leq 13, -17 \leq l \leq 17$	$-14 \leq h \leq 14, -14 \leq k \leq 13, -17 \leq l \leq 17$	$-11 \leq h \leq 8, -38 \leq k \leq 36, -12 \leq l \leq 8$
Reflections collected	23641	11946	11063
Independent reflections	4827 [R_{int} = 0.0530, R_{sigma} = 0.0464]	6426 [R_{int} = 0.0367, R_{sigma} = 0.0712]	5853 [R_{int} = 0.0927, R_{sigma} = 0.1467]
Data/restraints/parameters	4827/0/345	6426/87/404	5853/144/511
Goodness-of-fit on F^2	1.026	0.967	0.970
Final R indexes [I>=2σ (I)]	R_1 = 0.0457, wR_2 = 0.1043	R_1 = 0.0594, wR_2 = 0.1314	R_1 = 0.0681, wR_2 = 0.1410
Final R indexes [all data]	R_1 = 0.0775, wR_2 = 0.1187	R_1 = 0.1588, wR_2 = 0.1810	R_1 = 0.1577, wR_2 = 0.1864
Largest diff. peak/hole / e Å^{-3}	0.28/-0.24	0.14/-0.20	0.24/-0.24

5.- References

[1] Melchor Bañales, A. J.; Larsen, M. B. Thermal Guanidine Metathesis for Covalent Adaptable Networks. *ACS Macro Lett.,* **2020**, 9, 937–943.

[2] Frisch, M. J.; Trucks, G. W.; Schlegel, H. B.; Scuseria, G. E.; Robb, M. A.; Cheeseman, J. R.; Scalmani, G.; Barone, V.; Petersson, G. A.; Nakatsuji, H.; Li, X.;

Caricato, M.; Marenich, A. V.; Bloino, J.; Janesko, B. G.; Gomperts, R.; Mennucci, B.; Hratchian, H. P.; Ortiz, J. V.; Izmaylov, A. F.; Sonnenberg, J. L.; Williams-Young, D.; Ding, F.; Lipparini, F.; Egidi, F.; Goings, J.; Peng, B.; Petrone, A.; Henderson, T.; Ranasinghe, D.; Zakrzewski, V. G.; Gao, J.; Rega, N.; Zheng, G.; Liang, W.; Hada, M.; Ehara, M.; Toyota, K.; Fukuda, R.; Hasegawa, J.; Ishida, M.; Nakajima, T.; Honda, Y.; Kitao, O.; Nakai, H.; Vreven, T.; Throssell, K.; Montgomery, J. A.; Peralta, J. E.; Ogliaro, F.; Bearpark, M. J.; Heyd, J. J.; Brothers, E. N.; Kudin, K. N.; Staroverov, V. N.; Keith, T. A.; Kobayashi, R.; Normand, J.; Raghavachari, K.; Rendell, A. P.; Burant, J. C.; Iyengar, S. S.; Tomasi, J.; Cossi, M.; Millam, J. M.; Klene, M.; Adamo, C.; Cammi, R.; Ochterski, J. W.; Martin, R. L.; Morokuma, K.; Farkas, O.; Foresman, J. B.; Fox, D. J. Gaussian 16, Revision C.01. 2016.

[3] Bruker, SAINT+ v8.38A Integration Engine, Data Reduction Software, Bruker Analytical X-ray Instruments Inc., Madison, WI, USA, 2015.

[4] Bruker, SADABS 2014/5, Bruker AXS area detector scaling and absorption correction, Bruker Analytical X-ray Instruments Inc., Madison, Wisconsin, USA, 2014/5.

[5] Sheldrick, G. M. Crystal structure refinement with SHELXL. *Acta Crystallogr. Sect. A: Found. Crystallogr.* **2015**, *71*, 3–8.

[6] Sheldrick, G. M. A short history of SHELX. *Acta Crystallogr. Sect. A: Found. Crystallogr.* **2008**, *64*, 112–122.

[7] Dolomanov, O. V.; Bourhis, L. J.; Gildea, R. J.; Howard, J. A. K.; Puschmann, H. OLEX2: a complete structure solution, refinement and analysis program. *J. Appl. Crystallogr.* **2009**, *42*, 339–341.
